# Automatically Detected Microsleep Episodes in the Fitness-to-Drive Assessment

**DOI:** 10.3389/fnins.2020.00008

**Published:** 2020-01-23

**Authors:** Jelena Skorucak, Anneke Hertig-Godeschalk, Peter Achermann, Johannes Mathis, David R. Schreier

**Affiliations:** ^1^Institute of Pharmacology and Toxicology, University of Zurich, Zurich, Switzerland; ^2^Neuroscience Center Zurich, University of Zurich and ETH Zurich, Zurich, Switzerland; ^3^Sleep and Health Zurich, University of Zurich, Zurich, Switzerland; ^4^Children’s Hospital Zurich – Eleonore Foundation, Zurich, Switzerland; ^5^Department of Neurology, Inselspital, Bern University Hospital, University of Bern, Bern, Switzerland; ^6^The KEY Institute for Brain-Mind Research, Department of Psychiatry, Psychotherapy and Psychosomatics, University Hospital of Psychiatry Zurich, Zurich, Switzerland

**Keywords:** microsleep episodes, maintenance of wakefulness test, driving simulator, electroencephalography, sleepiness, wake-sleep transition zone, machine learning, fitness to drive

## Abstract

**Study Objectives:** Microsleep episodes (MSEs) are short fragments of sleep (1–15 s) that can cause dangerous situations with potentially fatal outcomes. In the diagnostic sleep-wake and fitness-to-drive assessment, accurate and early identification of sleepiness is essential. However, in the absence of a standardised definition and a time-efficient scoring method of MSEs, these short fragments are not assessed in clinical routine. Based on data of moderately sleepy patients, we recently developed the Bern continuous and high-resolution wake-sleep (BERN) criteria for visual scoring of MSEs and corresponding machine learning algorithms for automatic MSE detection, both mainly based on the electroencephalogram (EEG). The present study aimed to investigate the relationship between automatically detected MSEs and driving performance in a driving simulator, recorded in parallel with EEG, and to assess algorithm performance for MSE detection in severely sleepy participants.

**Methods:** Maintenance of wakefulness test (MWT) and driving simulator recordings of 18 healthy participants, before and after a full night of sleep deprivation, were retrospectively analysed. Performance of automatic detection was compared with visual MSE scoring, following the BERN criteria, in MWT recordings of 10 participants. Driving performance was measured by the standard deviation of lateral position and the occurrence of off-road events.

**Results:** In comparison to visual scoring, automatic detection of MSEs in participants with severe sleepiness showed good performance (Cohen’s kappa = 0.66). The MSE rate in the MWT correlated with the latency to the first MSE in the driving simulator (*r*_*s*_ = −0.54, *p* < 0.05) and with the cumulative MSE duration in the driving simulator (*r*_*s*_ = 0.62, *p* < 0.01). No correlations between MSE measures in the MWT and driving performance measures were found. In the driving simulator, multiple correlations between MSEs and driving performance variables were observed.

**Conclusion:** Automatic MSE detection worked well, independent of the degree of sleepiness. The rate and the cumulative duration of MSEs could be promising sleepiness measures in both the MWT and the driving simulator. The correlations between MSEs in the driving simulator and driving performance might reflect a close and time-critical relationship between sleepiness and performance, potentially valuable for the fitness-to-drive assessment.

## Introduction

Excessive daytime sleepiness (EDS) is estimated to be prevalent in around 20% of the general population (Pallesen et al., 2007; Ohayon, 2008; Swanson et al., 2011), while the risk of motor vehicle accidents or near-miss accidents is significantly higher among drivers suffering from EDS (Connor et al., 2002; Nabi et al., 2006; Ward et al., 2013; Garbarino et al., 2016; Bioulac et al., 2017; Gottlieb et al., 2018). Sleepiness can negatively affect reaction time and performance, similar or even more severe than driving with an illegal blood alcohol concentration (Dawson and Reid, 1997; Williamson and Feyer, 2000; Arnedt et al., 2001; Thomann et al., 2014). However, unlike the quantification of breath or blood alcohol concentrations, there is no method to accurately quantify sleepiness behind the wheel yet, and therefore, no legal standard exists (Williamson and Feyer, 2000; Schreier et al., 2018). Sleepiness and its impact on fitness to drive are currently assessed in a clinical setting by the use of multiple vigilance tests. The ability to resist falling asleep is most often assessed in the maintenance of wakefulness test (MWT) (Mitler et al., 1982). Although evidence regarding the accuracy of the MWT to predict real-world driving performance is scarce (Philip et al., 2008), evidence for the use of a driving simulator to assess fitness to drive in sleepy individuals is even more limited (Schreier et al., 2018). Nevertheless, existing evidence suggests that the MWT and the driving simulator are currently the best tools at hand for the fitness-to-drive assessment in sleepy patients (Banks et al., 2005; Sagaspe et al., 2007; Pizza et al., 2009).

Besides the discussions on which test(s) to use, a debate on how to define wakefulness and sleep was reactivated a few years ago by studies demonstrating the simultaneous co-existence of wakefulness and sleep, i.e., local sleep (Huber et al., 2004; Nobili et al., 2011; Vyazovskiy et al., 2011). This phenomenon is of particular relevance for the wake-sleep transition zone, which is not yet adequately addressed in the current version of the clinical scoring guidelines of the American Academy of Sleep Medicine (AASM) (Berry et al., 2018), neither from a topographical nor a temporal point of view. According to the AASM scoring criteria (Berry et al., 2018), wakefulness and the various sleep stages are scored in 30-s epochs, even though falling asleep is a process with rapid fluctuations between wakefulness and sleep (Ogilvie, 2001). Doghramji et al. (1997) published normative data using 10-s epochs for scoring MWT recordings. Our recently published Bern continuous and high-resolution wake-sleep (BERN) scoring criteria are even more sensitive, defining continuously scored microsleep episodes (MSEs) with a minimum duration as short as 1 s (Hertig-Godeschalk et al., 2019). Even though the BERN scoring criteria enable a more standardised scoring of MSEs, the very time-consuming process of visual MSE scoring might discourage sleep medicine physicians to consider MSEs in clinical practice. Consequently, we developed BERN-criteria-based algorithms for the automatic detection of MSEs using machine learning (Skorucak et al., 2019). Features derived from electroencephalogram (EEG) and electrooculogram (EOG) data of moderately sleepy patients with suspected disorders of various origin assessed in the MWT were used for training and testing. Since the feature-based algorithm was only tested in moderately sleepy patients, its applicability in individuals with more severe sleepiness remains unknown and needs to be investigated. Independent hereof, the implication of MSEs in the MWT and/or driving simulator on the judgement of fitness to drive needs to be clarified in order to determine the currently unknown clinical relevance.

The primary aim of this study was to evaluate the associations between MSEs in both the MWT and the driving simulator, and driving performance as measured by the standard deviation of lateral position and off-road events. The secondary aim was to test if our previously developed algorithm could also be applied to individuals with more severe sleepiness. For practical reasons, we first tested our algorithm in healthy participants before and after a full night of sleep deprivation. In a second step, assuming the algorithm to be applicable, we investigated the characteristics of automatically detected MSEs with respect to the two different test conditions (MWT and driving simulator) after sleep deprivation and analysed associations between MSEs and driving performance. A close association between MSEs and driving performance would highlight the relevance of MSEs in the fitness-to-drive assessment.

## Materials and Methods

### Study Population

Data of 24 healthy participants of a previous study (Schreier et al., 2015) were analysed. Four participants were excluded due to technical problems with the recording (one participant) or corrupted driving simulator data (three participants). In two out of the 20 participants, a disproportionately high number of automatically MSEs occurred in the driving simulator. The reason for this high number of (false positively) detected MSEs remained unclear. We decided to exclude these two recordings for further analyses, resulting in a final study sample of 18 participants (mean age 23.3 ± 1.3 years, eight females). The study was conducted in accordance with the principles of the Declaration of Helsinki and Swiss Law. The protocol was approved by the local ethics committee (KEK-number 185/06). Written informed consent was obtained from each participant.

### Procedure

Participants were obliged to follow a regular sleep-wake pattern during the 5 days preceding the measurements. Adherence to the protocol was assessed using actigraphy and a sleep diary. Measurements consisted of both a 40-min MWT and a 60-min driving simulator trial between 6 pm and 11 pm, followed by a full night of sleep deprivation and, again, a 40-min MWT and 60-min driving simulator trial between 7 am and 12 pm. Participants were allocated randomly for the test sequence (MWT-driving simulator or driving simulator-MWT) which remained the same before and after sleep deprivation.

### Assessments

Standardised recordings including the EEG (O1-M2, O2-M1, C3-M2, C4-M1, CZ-M1, F3-M2 and F4-M1), EOG (two eye channels), submental electromyogram, electrocardiogram, respiratory flow, and face videography with audio were performed both in the MWT and the driving simulator. Data were recorded with RemLogic^TM^ (Embla Systems LLC). The sampling rate was 200 Hz, and filters for EEG and EOG were set to 0.3 Hz high-pass, 70 Hz low-pass, and 50 Hz power line notch. Impedances were below 5 kΩ at the beginning of the recording.

The MWT was conducted following a standard protocol (Mitler et al., 1982). Participants were sitting on a chair in a semi-darkened room and were instructed to stay awake for as long as possible while keeping their eyes open. Each trial was terminated after either (a) 40 min or (b) the online identification of three consecutive epochs of N1 or one epoch of any other sleep stage according to the AASM scoring criteria (Berry et al., 2018).

The Divided Attention Steering Simulator (DASS, 3D Road Test; Stowood Scientific Instruments Ltd.) was used as a driving simulator. Performance data were recorded with a sampling rate of 20 Hz. The selected driving simulator scenario consisted of a virtual road, depicted by white lines on a black screen, with the position of the car on the road being displayed by its bonnet. To drive in the middle of the road was the only task for participants in this scenario. The driving simulator data were not recorded with the same device as the EEG, leading to a potential desynchronisation between EEG and driving simulator recordings of up to 1–2 s. The trial was terminated after either (a) 60 min, (b) the online identification of three consecutive epochs of N1 or one epoch of any other sleep stage according to AASM scoring criteria (Berry et al., 2018) or (c) in case of an off-road event lasting >15 s.

### Analyses

The previously developed feature-based deep learning algorithm for automatic MSE detection (long short-term memory neural network) (Skorucak et al., 2019) is based on the BERN scoring criteria, which define MSEs as occipital EEG fragments similar to N1 but lasting 1–15 s while eyelids are ≥80% closed in the face videography (Hertig-Godeschalk et al., 2019). Further, MSEs are characterised by a slowing in the EEG with dominant theta activity. Features were derived from occipital EEG derivations and the EOG: power in delta, theta, alpha and beta frequency range, ratio theta/(alpha + beta), eye movements and median EEG frequency. The algorithm was trained on MSEs occurring bilaterally and validated against MSEs occurring both unilaterally and bilaterally (pooled) in patients with suspected EDS (Skorucak et al., 2019). Since the algorithm was trained on moderately sleepy patients only, its performance on severely sleepy healthy participants had to be determined before further application. For this MWT data were used, as algorithm performance was previously assessed based on visual scoring of MWT data and more MSEs were expected to occur in the MWT than in the driving simulator. Due to the rather time-consuming process of visual MSE scoring, only MWT recordings of a randomly selected subgroup of 10 participants were visually scored by an expert (AHG) following the BERN criteria. Both MWT trials, before and after sleep deprivation, were analysed from the start (“lights off”) until the end (“lights on”). Overall (pooled across participants) and average performance measures were calculated: sensitivity, specificity, precision, accuracy and Cohen’s kappa coefficient. Sensitivity represents the proportion of MSEs that are correctly identified (true positives, algorithm MSEs corresponding to human scorer’s MSEs, divided by the sum of the true positives and false negatives), and specificity stands for the proportion of wakefulness that was correctly identified (true negatives divided by the sum of the true negatives and false positives). Accuracy is a measure combining sensitivity and specificity (correctly identified MSEs and wakefulness divided by the sum of the correctly and incorrectly identified ones). Precision represents proportion of correctly identified MSEs (algorithm corresponding to human scoring) out of the sum of true positives and false positives. Since our data consisted of a rather large number of negatives (wakefulness) compared to positives (MSEs), specificity and accuracy are biased measures since they rely on negatives. Sensitivity and precision provide information about true positives, and they are more informative about how well MSEs were detected. Furthermore, Cohen’s kappa coefficient is more robust than accuracy since it takes into account the possibility of agreement occurring by chance. The definitions of the different measures are described in more detail in Skorucak et al. (2019).

In the driving simulator, the lateral position of the vehicle was defined as the deviation of the vehicle’s centre to the centre of the road. Deviations larger than ± 1000 (arbitrary unit defined by the manufacturer) from the centre of the road were considered off-road events. For driving performance, the rate and the duration of off-road events as well as the standard deviation of lateral position (10-s moving window and overall) were calculated.

Except for the evaluation of algorithm performance, where the entire recordings were analysed, MWT and driving simulator trials were analysed from “lights off” until the first epoch of AASM-defined sleep (Berry et al., 2018). For all trials, the latencies from “lights off” to the onset of the first MSE, the first epoch of AASM-defined sleep (sleep latency), and the first off-road event were determined. In the absence of a MSE, sleep, or an off-road event, the corresponding latency was set to the end of the trial (i.e., to 40 min or 60 min). The rate (#/min) and cumulative duration (% of time) of MSEs and off-road events were calculated relative to the duration from “lights off” until sleep occurred, as well as in 1- and 10-min bins. Standard deviation of lateral position was calculated from “lights off” until sleep occurred, as well as in 1-min bins.

Analyses were performed using MATLAB (R2018a, MathWorks Inc., Natick, MA, United States) and Stata (StataCorp. 2017, Stata Statistical Software: Release 15.1. College Station, TX: StataCorp LLC). Spearman’s rank coefficients are reported for correlations. The Kruskal–Wallis and the Wilcoxon signed-rank test were used for comparisons. A significance level of *p* < 0.05 (two-tailed) was applied in all tests.

## Results

### Visual Scoring and Automatic Detection of MSEs

**FIGURE 1:**
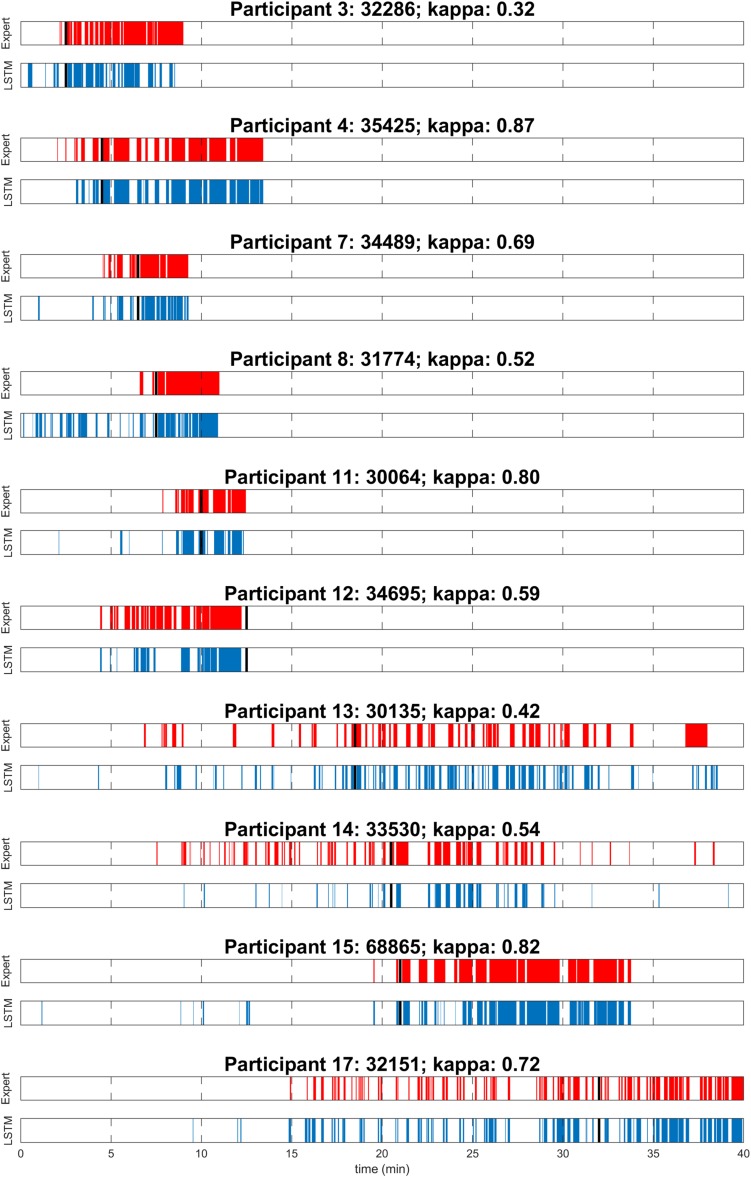
Visual expert scoring (red) and automatic detection with long short-term memory neural network (LSTM; blue) of microsleep episodes (MSEs) for each of the 10 participants assessed in the maintenance of wakefulness test (MWT) after sleep deprivation. Sleep onset is indicated (black). Participants are numbered according to Figure 3A.

**FIGURE 2:**
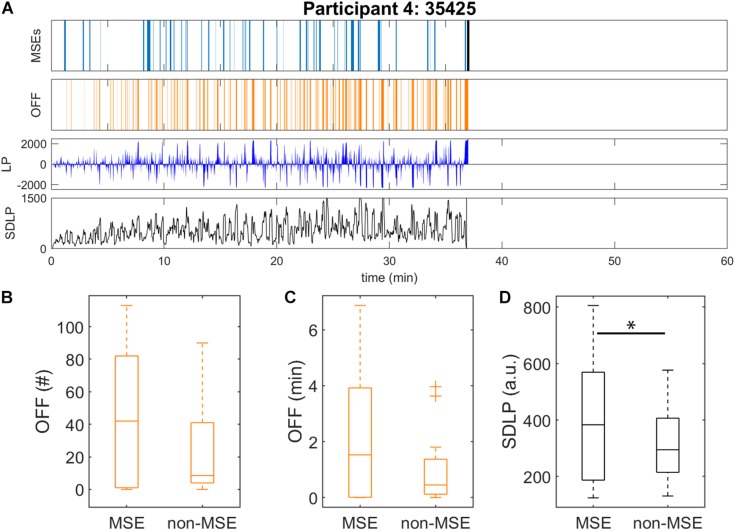
Automatic detection of microsleep episodes (MSEs) and driving performance in the driving simulator after sleep deprivation. Data were analysed until sleep onset. **(A)** Displayed for one representative participant: automatically detected MSEs (light blue) with sleep onset (black bar), off-road events (OFF, orange), lateral car position (LP, dark blue; 0 = centre of the road, off-road > ± 1000) and standard deviation of lateral position (SDLP, black) calculated in a 10-s moving window. Driving performance and EEG derived MSEs might be 1–2 s out of synchronisation. Data of all participants are provided in Supplementary Figure S2. Driving performance of all participants as measured by **(B)** number and **(C)** duration of off-road events (OFF), and **(D)** SDLP (10-s moving window), were calculated for the subsequent minute after each MSE onset (“MSE”), and the rest of the recording (“non-MSE”). ^∗^Significant difference (*p* < 0.05).

To evaluate the performance of the algorithm, both in the near absence as well as in the abundant presence of MSEs, MWT trials before and after sleep deprivation were included for overall performance calculations (Table 1). As expected, the number of MSEs in the MWT before sleep deprivation was low. In fact, MSEs were only observed in one participant. Further, hardly any false positives (MSEs) were automatically detected. In the MWT after sleep deprivation, many MSEs were present in all participants, which allowed for an assessment of performance on an individual level (Figure 1 and Table 1). Visual inspection of the recordings illustrated in Figure 1 revealed that false-positive MSEs in participant 8 at the beginning of the recording were mostly due to the eye closure criteria (MSEs that were detected by the algorithm but could not be scored following BERN criteria due to open eyes), and false negatives in participant 12 were probably related to a noisy EEG. The algorithm identified MSEs with good sensitivity (70.9 ± 5.0%) and precision (82.7 ± 4.5%), and high specificity (90.7 ± 3.0%) and accuracy (84.7 ± 2.9%). Specificity and accuracy were high as they rely on the predominant state (wakefulness). Cohen’s kappa revealed substantial identification (0.63 ± 0.06). Based on these results, the algorithm was applied to MWT but also driving simulator recordings of all 18 participants after sleep deprivation, without performing additional visual scoring (Figure 2A and Supplementary Figures S1, S2).

**TABLE 1:** Performance of automatic microsleep episode (MSE) detection compared to visual scoring in the maintenance of wakefulness test (MWT) trials of 10 participants before and after sleep deprivation.

	Sensitivity (%)	Specificity (%)	Precision (%)	Accuracy (%)	Cohen’s Kappa
Before and after sleep deprivation (overall)	70.0	95.7	66.2	93.0	0.64
After sleep deprivation (overall)	70.0	93.3	81.1	86.6	0.66
After sleep deprivation (mean ± standard error of the mean)	70.9 ± 5.0	90.7 ± 3.0	82.7 ± 4.5	84.7 ± 2.9	0.63 ± 0.06

*Overall performance (pooled for all 10 participants, either across both trials or across the trial after sleep deprivation) and mean performance (across each participant in the trial after sleep deprivation) was calculated based on a 200-ms resolution. Assessing the performance of the first trial (before sleep deprivation) separately was not meaningful, as MSEs were only present in one participant.*

### MSEs in the MWT and the Driving Simulator

**FIGURE 3:**
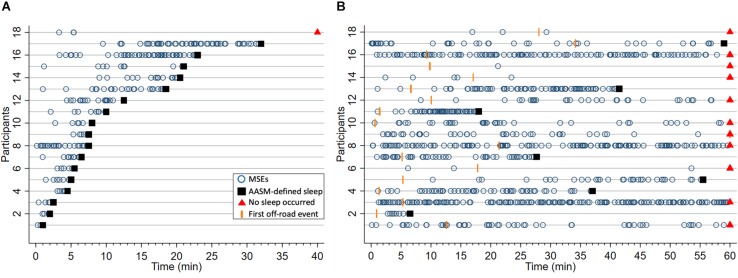
Occurrence of microsleep episodes (MSEs, o) and latencies to the first off-road event (OFF, I) until sleep onset (■), or if no sleep occurred the end of trial (▲), in **(A)** the maintenance of wakefulness test (MWT) and **(B)** the driving simulator after sleep deprivation. In case no MSE, sleep or OFF occurred, latencies were set to either sleep onset or the end of the test, whichever came first. Each horizontal line represents one of the 18 participants, ordered by the latency to sleep onset in the MWT. This participant numbering was also applied to the other figures.

**FIGURE 4:**
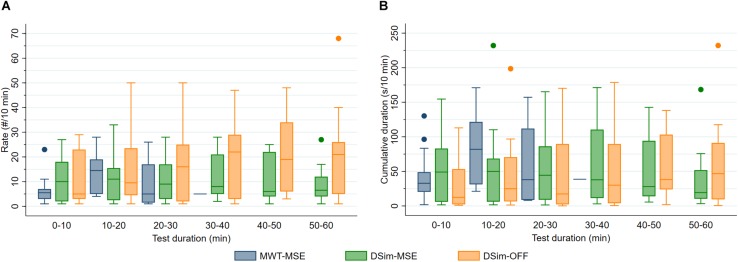
**(A)** Rate (#/10 min) and **(B)** cumulative duration (s/10 min) of microsleep episodes (MSEs) and off-road events (OFF) during the maintenance of wakefulness test (MWT) and driving simulator (DSim) after sleep deprivation. Data were analysed until sleep onset and displayed in 10-min bins (4 boxplots for the MWT and 6 for the DSim).

After sleep deprivation, the latency to the first MSE was significantly shorter compared to the sleep latency, in both the MWT and the driving simulator (*p* < 0.01, Table 2). While the latencies to the first MSE in the MWT and the driving simulator were comparable (*p* = 0.81), the sleep latency in the MWT was much shorter than the sleep latency in the driving simulator (*p* < 0.01). Sleep in the MWT usually appeared within 10 min after the first MSE (12 participants), whereas mostly no sleep but many MSEs occurred in the driving simulator (11 participants, Figure 3). A 34.4% share of the MSEs were shorter than 3 s in the MWT and 38.5% in the driving simulator. The MSE rate was higher and the cumulative MSE duration longer in the MWT than in the driving simulator (*p* < 0.05, Table 2). Analysing trials in 10-min intervals (Figure 4), the cumulative MSE duration increased when comparing the 0–10 min to the 10–20 min bin in the MWT (median [IQR]: 32.8 [20.4–48.8]% versus 81.9 [31.2–121.6]%, *p* < 0.05) while no such increase could be observed in the driving simulator (49.0 [6.0–83.0]% versus 49.9 [6.2–68.5]%, *p* = 0.75).

**TABLE 2:** Overview of sleepiness and driving performance measures in the maintenance of wakefulness test (MWT) and the driving simulator (DSim) after sleep deprivation.

	MWT	DSim
MSE-L (min)	2.43 [0.97–3.36]	1.20 [0.94–6.16]
AASM-L (min)	7.75 [5.00–20.50]	60.00 [41.50–60.00]
Difference between MSE-L and AASM-L (min)	6.44 [2.17–17.51]	52.77 [38.80–58.92]
Median MSE duration (s)	4.50 [4.20–6.40]	3.40 [2.90–4.00]
MSE rate (#/min)	1.29 [0.73–1.83]	0.74 [0.48–1.59]
Cumulative MSE duration (%)	13.80 [9.35–22.53]	5.58 [3.09–12.62]
OFF-L (min)		9.57 [5.19–17.82]
Median OFF duration (s)		1.54 [1.15–2.52]
OFF rate (#/min)		1.63 [0.10–2.85]
Cumulative OFF duration (%)		4.78 [0.21–14.19]
SDLP		1463.90 [493.04–3034.65]

*Data were analysed until sleep onset. Median and interquartile range are reported: latency to the first microsleep episode (MSE-L), latency to AASM-defined sleep (AASM-L), latency to the first off-road event (OFF-L), rate and duration of MSEs and off-road events (OFF), and the standard deviation of lateral position (SDLP). The cumulative duration of MSEs and OFF were calculated relative to the duration from “lights off” until AASM-L of each trial (%). Median values were first determined for each MSE or OFF per participant, and then across participants.*

In both tests, the latency to the first MSE correlated with the MSE rate, and the MSE rate with the cumulative MSE duration (Tables 3A,B). In the MWT, the sleep latency correlated with the latency to the first MSE, the MSE rate and the cumulative MSE duration (Table 3A). In the driving simulator, associations were found between the sleep latency and the difference between the latency to the first MSE and sleep latency (Table 3B). Comparing across tests (MWT versus driving simulator), correlations between MSE rates and between cumulative MSE durations were observed (Table 3C). Scatterplots of relevant associations are illustrated in Supplementary Figure S3.

**TABLE 3:** Correlation coefficients (Spearman’s rank) for sleepiness and driving performance measures after sleep deprivation are reported: (A) maintenance of wakefulness test (MWT), (B) driving simulator (DSim), and (C) between the MWT and the DSim.

	(A) MWT										

			MSE-L (min)	AASM-L (min)	Difference between MSE-L and AASM-L (min)		Median MSE duration (s)		MSE rate (#/min)	Cumulative MSE duration (%)	
MSE-L (min)											
AASM-L (min)			0.60**								
Difference between MSE-L and AASM-L (min)			0.45	0.97**							
Median MSE duration (s)			0.11	−0.45	−0.57*							
MSE rate (#/min)			−0.53*	−0.52*	−0.41		0.11						
Cumulative MSE duration (%)			−0.27	−0.50*	−0.48*		0.55*		0.77**				

	**(B) DSim**										

	**MSE-L** **(min)**	**AASM-L** **(min)**	**Difference** **between MSE-L** **and AASM-L (min)**	**Median** **MSE** **duration (s)**	**MSE rate** **(#/min)**	**Cumulative** **MSE** **duration (%)**	**OFF-L** **(min)**	**Median** **OFF** **duration (s)**	**OFF rate** **(#/min)**	**Cumulative** **OFF** **duration (%)**	**SDLP**

MSE-L (min)											
AASM-L (min)	0.09										
Difference between MSE-L and AASM-L (min)	−0.52*	0.73**									
Median MSE duration (s)	−0.08	−0.47	−0.27								
MSE rate (#/min)	−0.62**	−0.42	0.02	0.72**							
Cumulative MSE duration (%)	−0.57*	−0.39	−0.00	0.76**	0.99**						
OFF-L (min)	0.03	0.58**	0.45	−0.38	−0.35	−0.32					
Median OFF duration (s)	−0.03	−0.59*	−0.44	0.49*	0.44	0.42	−0.67**				
OFF rate (#/min)	−0.15	−0.66**	−0.39	0.47*	0.46	0.43	−0.83**	0.81**			
Cumulative OFF duration (%)	−0.14	−0.73**	−0.46	0.53*	0.51*	0.49*	−0.81**	0.91**	0.97**		
SDLP	−0.11	−0.72**	−0.53*	0.43	0.45	0.42	−0.84**	0.83**	0.83**	0.90**	
											

	**(C) DSim and MWT**										

**MWT**			**MSE-L** **(min)**	**AASM-L** **(min)**	**Difference between MSE-L** **and AASM-L (min)**		**Median MSE** **duration (s)**		**MSE rate** **(#/min)**	**Cumulative MSE** **duration (%)**
									
**DSim**											

MSE-L (min)			0.16	0.07	0.09		−0.06		−0.54*	−0.44
AASM-L (min)			0.19	0.25	0.17		0.14		−0.21	−0.04
Difference between MSE-L and AASM-L (min)			0.05	0.04	−0.03		0.19		0.28	0.28
Median MSE duration (s)			−0.36	−0.46	−0.45		0.33		0.36	0.29
MSE rate (#/min)			−0.44	−0.28	−0.23		0.14		0.63**	0.52*
Cumulative MSE duration (%)			−0.44	−0.30	−0.26		0.22		0.62**	0.58*
OFF-L (min)			0.38	0.41	0.36		−0.12		−0.10	−0.05
Median OFF duration (s)			−0.35	−0.26	−0.17		0.10		0.35	0.21
OFF rate (#/min)			−0.38	−0.43	−0.38		0.28		0.32	0.30
Cumulative OFF duration (%)			−0.38	−0.39	−0.32		0.19		0.36	0.27
SDLP			−0.44	−0.36	−0.26		−0.03		0.31	0.12

Significant correlations are indicated in bold (*p < 0.05, **p < 0.01). Our correlational analyses were of exploratory character and thus no corrections to *p*-values were performed. The latency to the first microsleep episode (MSE-L), latency to AASM-defined sleep (AASM-L), latency to the first off-road event (OFF-L), rate and duration of MSEs and off-road events (OFF), and standard deviation of lateral position (SDLP) were analysed until AASM-defined sleep onset.

In both the MWT and driving simulator, the MSE rate and the cumulative MSE duration widely varied (Table 2 and Figure 3). In the MWT, the relationship between the occurrence of MSEs and the sleep latency revealed two distinct groups. In case of a sleep latency <15 min, multiple MSEs occurred already from the very beginning of the trial and most MSEs occurred within the first 10 min of the trial; thus, participants were labelled to be “very sleepy” (Figure 3A and Supplementary Figures S1, S4, participants 1–12). In case of a sleep latency ≥15 min, MSEs did not occur from the very start of the trial but rather between 10 and 20 min; thus, participants were labelled as “less sleepy” (Figure 3A, Supplementary Figures S1, S4, participants 13–18). When comparing these two groups, significant differences were found for the difference between the latency to the first MSE and the sleep latency (3.01 [1.77–6.44] min versus 19.74 [17.51–22.47] min, *p* < 0.01), the median MSE duration (5.45 [4.30–7.50] s versus 4.20 [2.80–4.20] s, *p* < 0.05), the cumulative MSE duration (15.5 [11.2–26.7]% versus 6.4 [2.8–15.1]%, *p* < 0.05), and (by definition) the sleep latency (6.00 [3.50–7.75] min versus 22.00 [20.50–32.00] min, *p* < 0.01). In the driving simulator, no such differences between the two groups occurred.

### Sleepiness and Driving Performance

The test sequence, MWT-driving simulator or driving simulator-MWT, had no significant impact on MSEs or driving performance. In the driving simulator, the median MSE duration, the MSE rate, and the cumulative MSE duration all moderately correlated with the cumulative duration of off-road events (Table 3B). Furthermore, the median MSE duration moderately correlated with the median off-road duration (Table 3B). All driving performance measures correlated with the sleep latency but not with the latency to the first MSE (Table 3B). No correlations between MSEs or the sleep latency in the MWT and driving performance were observed.

Comparing “less sleepy” and “very sleepy” participants, driving performance did not differ significantly. However, in “very sleepy” participants the latency to the first off-road event was somewhat shorter (5.31 [1.38–15.21] min versus 13.45 [9.33–28.05] min, *p* = 0.13) and longer off-road events tended to occur toward the end of the trial (cumulative off-road duration in the 50–60 min bin: 58.10 [24.30–91.05]% versus 25.33 [5.03–79.30]%, *p* = 0.20; Supplementary Figure S4).

Driving performance, as measured by the rate (*p* = 0.19) and cumulative duration (*p* = 0.11) of off-road events and standard deviation of the lateral position (*p* < 0.05), tended to be slightly worse during the 1-min segments after a MSE was detected, compared to all segments without MSEs (Figures 2B–D). For the relationship between MSEs and driving performance, mainly three different patterns were observed. In a first pattern, the MSE rate increased over time while driving performance, measured by an increased rate of off-road events and increased standard deviation of the lateral position, gradually deteriorated in parallel (Supplementary Figure S2, e.g., participants 11 and 13, and Supplementary Figure S5A). In a second pattern, participants were able to successfully compensate for their sleepiness, resulting in only a few MSEs and the maintenance of good driving performance (Supplementary Figure S2, e.g., participants 6 and 18, and Supplementary Figure S5B). In the last pattern, no obvious association between MSEs and driving performance could be observed (Supplementary Figure S2, e.g., participants 8 and 15, and Supplementary Figure S5C).

## Discussion

Independent of the test, automatically detected MSEs provide a valuable measure for sleepiness with potential relevance for the fitness-to-drive assessment. Even though the latency to the first MSE was comparable between the MWT and the driving simulator, sleep latency differed substantially. Both the rate and cumulative duration of automatically detected MSEs correlated between MWT and driving simulator. Driving performance correlated with MSEs in the driving simulator, but not with MSEs in the MWT.

The feature-based neural network algorithm for the automatic MSE detection showed good performance when applied to MWT data of participants with a broad range of sleepiness levels as performance in this study was assessed in conditions with high level of alertness (before sleep deprivation) and a high level of sleepiness (after sleep deprivation), and previously in moderately sleepy patients (Skorucak et al., 2019). Performance was better than the one reported for the automatic detection of N1 sleep (Sriraam et al., 2016; Malafeev et al., 2018; Stephansen et al., 2018). The algorithm showed substantial identification, similar to the performance on data from moderately sleepy patients which were used for training of the algorithm, with a slightly lower Cohen’s kappa coefficient (0.64 versus 0.75) (Skorucak et al., 2019). As expected, specificity (i.e., the ability to correctly detect negatives or wakefulness) and accuracy had large values due to the rather large amount of wakefulness in the data irrespective of the actual MSE detection performance. More importantly, sensitivity and precision showed good performance, providing a fairer indication of the ability of the algorithm to correctly detect positives or MSEs. The current approach for automatic MSE detection is based on deep learning with engineered features. The advantage of having engineered features as inputs for the neural network is having more control over the input data. However, working with raw data brings faster preprocessing as no features need to be calculated. Another MSE detection algorithm using deep learning based on raw data is currently under development [Malafeev et al., Automatic detection of microsleep episodes with deep learning (unpublished)].

Similar to moderately sleepy patients in the MWT (Hertig-Godeschalk et al., 2019), the latency to the first MSE among severely sleepy participants (after sleep deprivation) was shorter than the sleep latency in both the MWT and the driving simulator. Both the latency to the first MSE and the sleep latency were approximately 14 min shorter in sleep-deprived participants compared to moderately sleepy patients (Hertig-Godeschalk et al., 2019), reflecting the extreme sleep pressure after a full night of sleep deprivation. Most participants did not fall asleep in the driving simulator (sleep latency was set to 60 min) which could have confounded the results but also shows the possible limitation of this measure. Corresponding to previous research showing the value of EEG-based MSEs to objectively assess sleepiness (Harrison and Horne, 1996; Tirunahari et al., 2003; Bougard et al., 2018), we argue that MSEs could provide a more accurate and stable measure for sleepiness, compared to the sleep latency. Applying a minimum MSE duration of 3 s, the latency to the first MSE has been reported to facilitate differentiation among sleep apnoea patients with a sleep latency between 12.8 and 32.6 min in the MWT, into those who are likely to be either more susceptible or more resistant to sleepiness (Morrone et al., 2019). The latency to consolidated sleep is known to be affected by motivation (Bonnet and Arand, 2005). We speculate that the latency to the first MSE is less affected by motivation and thereby represents a more objective marker of sleepiness severity. Hence, the difference between the latency to the first MSE and that to sleep might be a measure for the compensational capacity while resisting the onset of consolidated sleep. Maintaining wakefulness without any interaction in the rather passive MWT condition is much more difficult compared to the active condition in the driving simulator.

In the current study, the latency to the first MSE correlated with the MSE rate in both test conditions, which emphasizes the importance of the first MSE as a first objective sign for sleepiness. Both MSE rate and the cumulative MSE duration might reflect sleepiness severity, as correlations between the two measures were observed not only within the MWT and driving simulator but also between the two tests. In addition, both the MSE rate and the cumulative MSE duration negatively correlated with the sleep latency in the MWT. This is contradictory to the results of another study among sleep-restricted healthy individuals in the MWT, defining EEG-based MSEs with a minimum duration of 3 s, where no such correlations were observed (Bougard et al., 2018). In our study, a substantial number of MSEs were shorter than 3 s in both the MWT and the driving simulator. This might explain why our findings differ from those of Bougard et al. (2018), while simultaneously underlining the need of broadly accepted MSE scoring criteria with a shorter minimum duration (Hertig-Godeschalk et al., 2019) than previously used (Harrison and Horne, 1996; Tirunahari et al., 2003; Bougard et al., 2018).

The MSE rate and cumulative MSE duration were higher in the MWT compared to the driving simulator. In the MWT, a series of MSEs occurred rather shortly after the first MSE and was soon followed by sleep, a finding that was also observed in moderately sleepy patients (Hertig-Godeschalk et al., 2019). In the driving simulator, MSEs occurred less frequently than in the MWT and in most participants no sleep appeared until the end of the trial. These differences between the test conditions are most probably explained by the different unmasking effects of sleepiness: the MWT is a monotonous test in a non-stimulating and almost entirely dark environment, whereas the driving simulator represents a more active situation taking place with increased ambient light due to the simulator screen, requiring continuous cognitive attention and steering reactions. Compared to the MWT, the condition of the driving simulator enables participants to fight their sleepiness more successfully and therefore no sleep appeared until the end of the test in most driving simulator trials, explaining the different MSE rates between the two test conditions.

Reliable clinical and on-road tools to precisely assess sleepiness and its impact on performance are urgently sought. Combining neurophysiological measures with performance data might be a valid methodological approach (Moller et al., 2005). The positive correlations between (rate and duration of) MSEs in the driving simulator and driving performance (off-road events and standard deviation of lateral position) in our study support the assumption that appearance of MSEs and their distribution over time are relevant measures of sleepiness in simulated driving.

Among participants with high rates of both MSEs and off-road events, we observed an increasing MSE rate in parallel with both an increasing rate of off-road events and increasing standard deviation of lateral position values over the course of the driving test. These results corroborate previous studies, where the standard deviation of lateral position, speed variability, and rate of off-road events correlated with (increasing) sleepiness (Arnedt et al., 2001; Philip et al., 2005; Baulk et al., 2008; Sandberg et al., 2011; Akerstedt et al., 2013; Forsman et al., 2013; Hallvig et al., 2014). Moreover, driving performance, including for example the standard deviation of lateral position, tended to be worse in 1-min segments following a MSE compared to the rest of the recording. This might point to a causal relationship between MSEs and driving performance.

No correlations between MSE measures or sleep latency in the MWT and driving performance were found, also not in “very sleepy” participants. In contrast, other studies found an association between the sleep latency in the MWT (0–19 min) and the rate of off-road events as well as standard deviation of lateral position in a driving simulator (Sagaspe et al., 2007; Philip et al., 2013). The different findings might be explained by the less severe sleepiness, but potentially affected compensational capacities among the patients in these studies, compared to the more severe sleepiness but intact compensational capacity of our healthy sleep-deprived participants. Another reason could be that MSEs as short as 1 s might be less often accompanied by a similar severity in driving impairment that is associated with longer sleep fragments of at least half a 30-s epoch. Further studies are needed to clarify whether high MSE rates and/or shorter MSE durations will impose a relevant real-road driving risk.

In the MWT, we could differentiate between two groups of “very” or “less” sleepy participants based on the sleep latency and the occurrence of MSEs. In the driving simulator, three different patterns could be differentiated based on the relation between sleepiness, as measured by cumulative MSE duration, and driving performance: (A) increasing sleepiness combined with impairment of driving performance, (B) hardly sleepy and good driving performance, and (C) no obvious association between sleepiness and driving performance. Interestingly, not all participants belonging to one group in the MWT could be categorised into the same pattern in the driving simulator, and vice versa. This confirms the notion that sleepiness should not be assessed by a single test (Mathis and Hess, 2009). The differences between participants regarding MSEs and driving performance could be explained by various intra-individual compensation mechanisms and susceptibility to sleep deprivation (Leproult et al., 2003; Van Dongen et al., 2004; Ingre et al., 2006), and both could influence the (strength) of the relations investigated in this study. Even though we found correlations between MSEs in the driving simulator and driving performance when analysing data on an overall level, correlations on an individual level were not present in some participants. This is contradictory to other studies where intra-individual analyses, as compared to inter-individual analysis, provided more consistent correlations between changes in the EEG and performance (Torsvall and Akerstedt, 1987; Makeig and Inlow, 1993). Again, we cannot exclude the possibility that isolated or even series of short-lasting MSEs have a different impact on driving performance compared to longer-lasting sleep fragments. At the same time, the rather monotonous driving scenario that was chosen in our study in order to unmask sleepiness efficiently, including rare and only wide curves, might lead to less frequent steering corrections and off-road events.

### Limitations and Outlook

The extreme sleepiness of participants in this study made visual classification of MSEs even more challenging and time-consuming compared to the scoring in moderately sleepy patients (Hertig-Godeschalk et al., 2019). The higher level of sleepiness and the application to the driving simulator data, compared to training on data of moderately sleepy patients in the MWT only, might have affected the performance of the algorithm. We hypothesise that among sleep-deprived participants MSEs with open eyes might occur in the MWT and maybe even more frequently in the driving simulator, since sleep and performance lapses with open eyes are possible (Johns, 2003; Jiřina et al., 2010). During higher levels of sleepiness, eye-opening could affect EEG activity differently than in a condition of full alertness, as alpha activity during drowsiness varies when the eyes are opened (decreased alpha) compared to when the eyes are closed (increased alpha) (Oken et al., 2006; Gorgoni et al., 2014). Without considering eye closure, automatically detected MSEs based on EEG and EOG might correspond to “real MSEs” since the automatic MSE detection is likely to identify EEG patterns that are not always visible to a human scorer (Schulz, 2008). Even though these events are defined as “microsleep episode candidates” in the BERN criteria (Hertig-Godeschalk et al., 2019), they do not fulfil the criteria to be visually scored as MSEs (≥80% eye closure criterion) and are consequently detected as false positives by the algorithm. As discussed earlier, the driving simulator differs from the MWT not only regarding the testing environment (e.g., light conditions) but also the performed task (e.g., merely sitting in a chair or driving a simulator). It is known that alpha activity can be blocked by light influx due to eye-opening, other afferent stimuli, and mental activities (Berger, 1929; Niedermeyer, 1993). The limitation of this paper is the lacking validation of the algorithm in an active rather than the passive setting in which it was developed. The BERN criteria would need to be re-evaluated for the different test condition, particularly the eye closure criterion, and the MSE detection algorithm might need to be retrained on driving simulator data in future. Unfortunately, in the scope of this study it was not possible to perform these steps for the active wake validation. Further, much more data than currently available would be needed for the training and testing of the algorithm in the driving simulator condition.

Although the assessment of MSEs leads to a more detailed characterisation of the borderland between wakefulness and sleep, ambiguous EEG-fragments not attributable to clear wakefulness or sleep (for example reflecting drowsiness) remain (Hertig-Godeschalk et al., 2019). Further studies are needed to determine the potential relevance of such EEG-fragments in relation to the level of sleepiness and driving performance.

It has been postulated that frontal regions are more susceptible to sleep deprivation than other brain regions (Horne, 1993; Cajochen et al., 1999). Therefore, investigating the topographical distribution of MSEs across the scalp might reveal new insights into the phenomenon of local sleep (Huber et al., 2004; Vyazovskiy et al., 2011; D’Ambrosio et al., 2019) and its influence on driving performance (Ahlstrom et al., 2017). The inclusion of other brain regions in the automatic detection of MSEs as well as their relationship with driving performance is planned for future studies.

As there is hardly any knowledge about possible relationships between MSEs and driving performance, our correlational analyses were of exploratory character and thus no corrections of the *p*-values were performed. In view of the multiplicity of risk factors, aside from the sleepiness level itself, such as sleepiness perception and risk-taking behaviour, future work has to reveal which relations are most relevant for safe real road driving in combination with which additional risk factors.

## Conclusion

Automatically detected MSEs as short as 1 s in the driving simulator, which would be missed by classical sleep scoring, revealed a significant relationship with driving performance. As MSEs are currently not routinely scored in clinical practice, future integration of a more refined MSE detection into the clinical assessment of sleepiness might improve both the diagnostic value of EEG-based vigilance tests as well as the judgment of fitness to drive.

## Data Availability Statement

The datasets generated for this study are available on request to the corresponding author.

## Ethics Statement

The studies involving human participants were reviewed and approved by the Kantonale Ethikkommission für die Forschung, Bern, Switzerland. The patients/participants provided their written informed consent to participate in this study.

## Author Contributions

All authors conceived the study, contributed to the revision of the manuscript, and read and approved the submitted version. DS collected the data. JS and AH-G performed the data analysis and wrote the first draft of the manuscript.

## Conflict of Interest

The authors declare that the research was conducted in the absence of any commercial or financial relationships that could be construed as a potential conflict of interest.
