# Supplementary material for: Automatically Detected Microsleep Episodes in the Fitness-to-Drive Assessment
Source: Front Neurosci. 2020 Jan 23;14:8. doi: 10.3389/fnins.2020.00008 (PMC6990913; doi:10.3389/fnins.2020.00008)
Supplement: Supplementary file 1 [file Table_1.DOCX]

Supplementary Material

## **
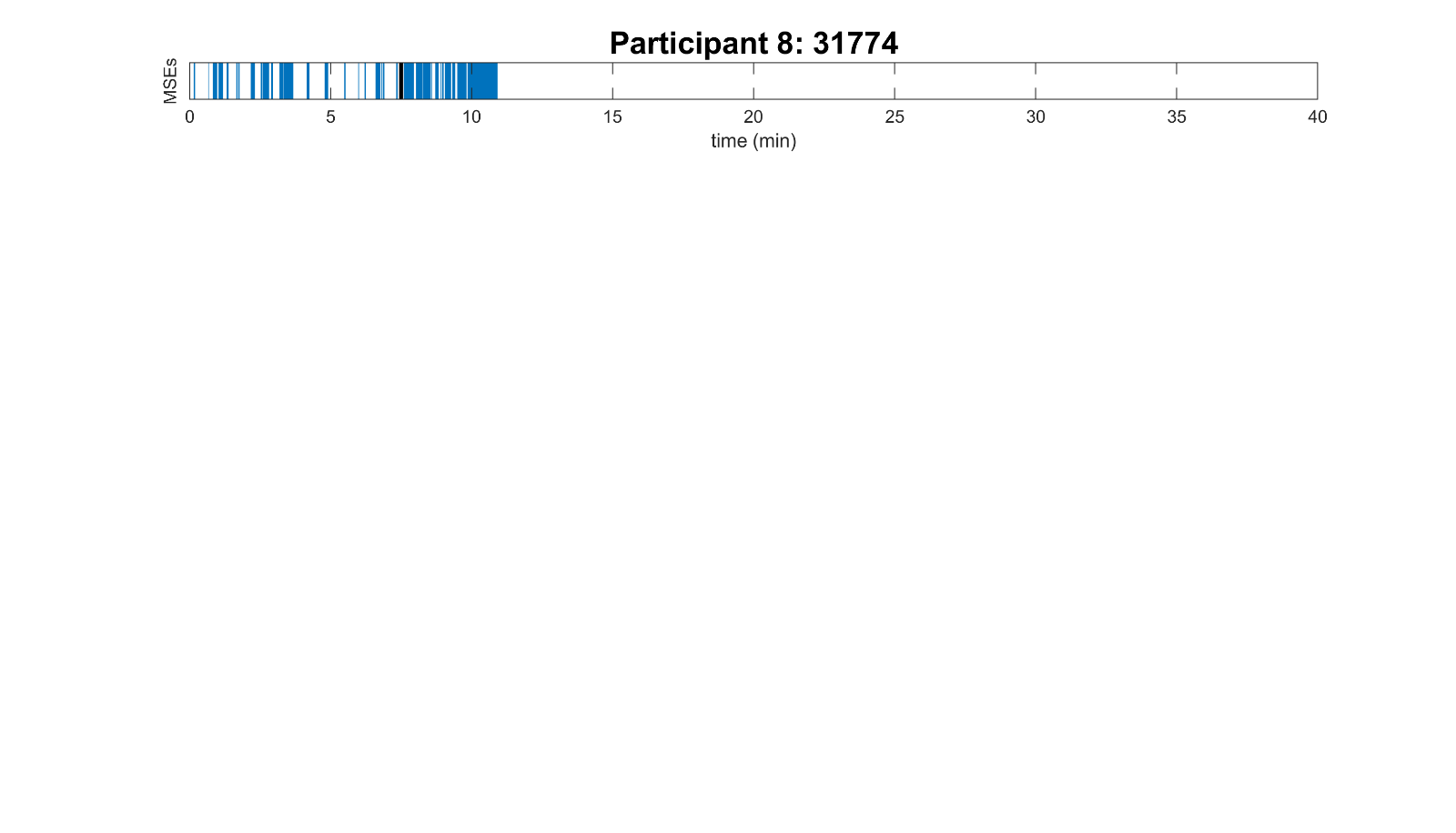
**
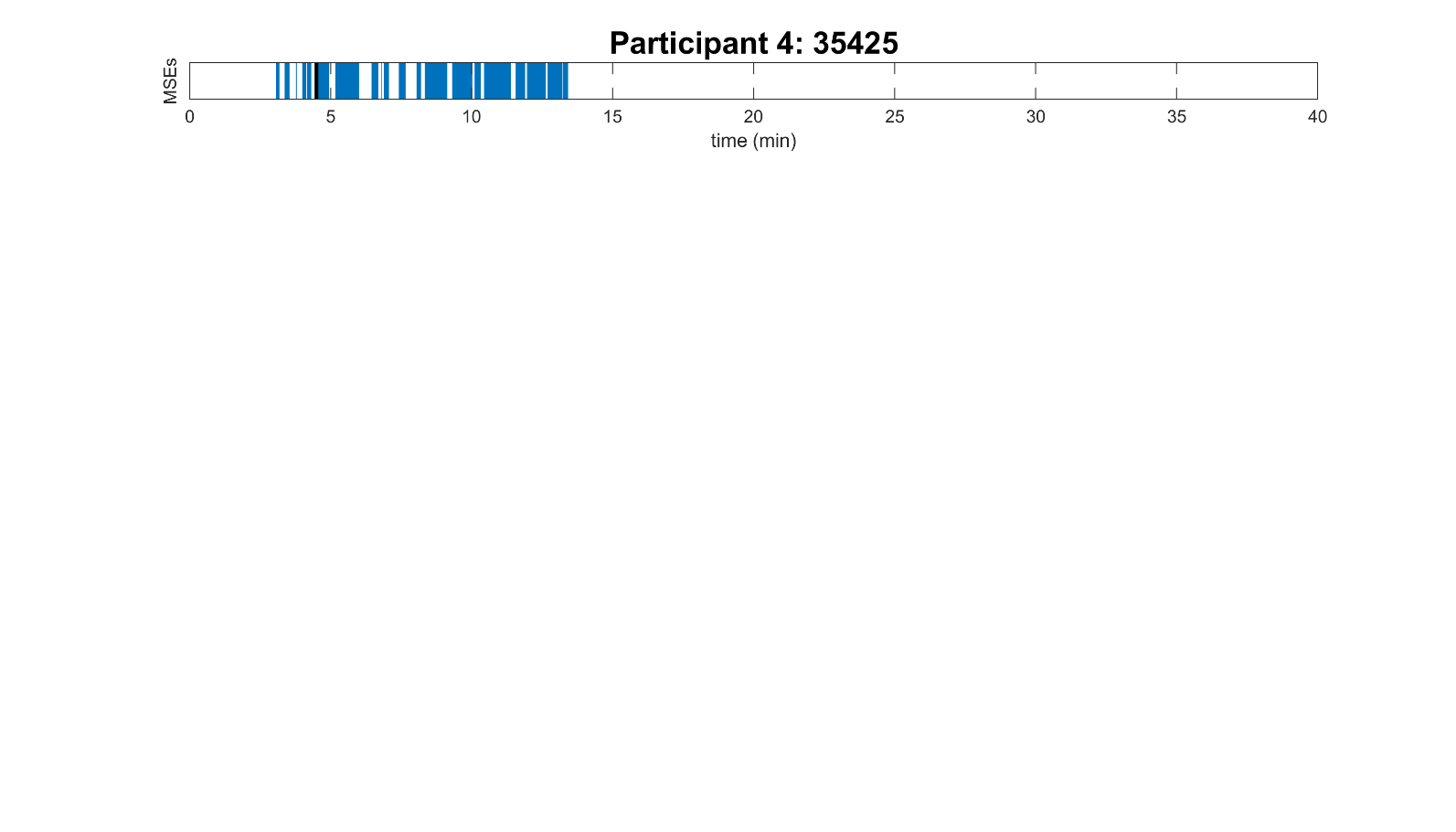

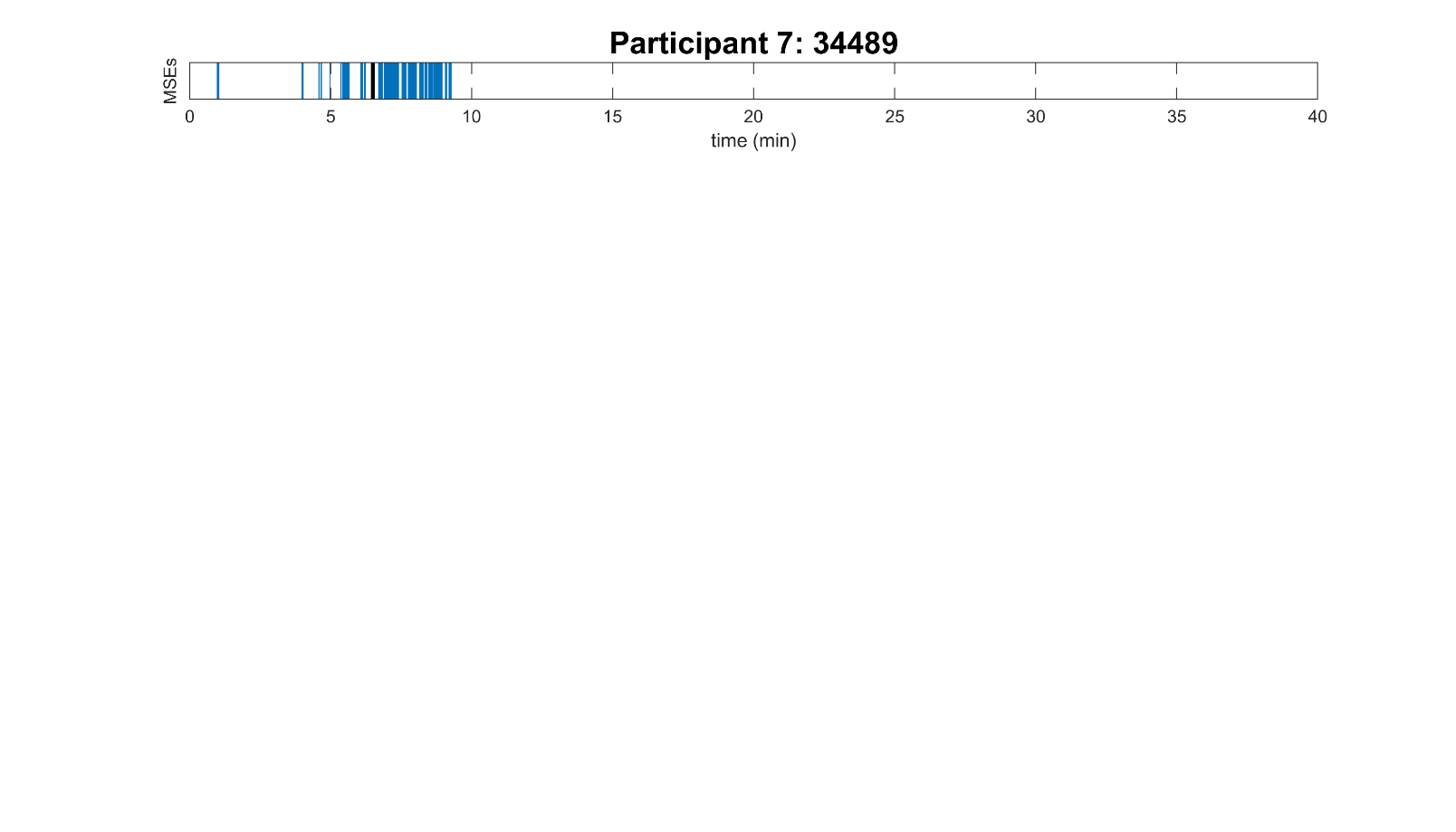

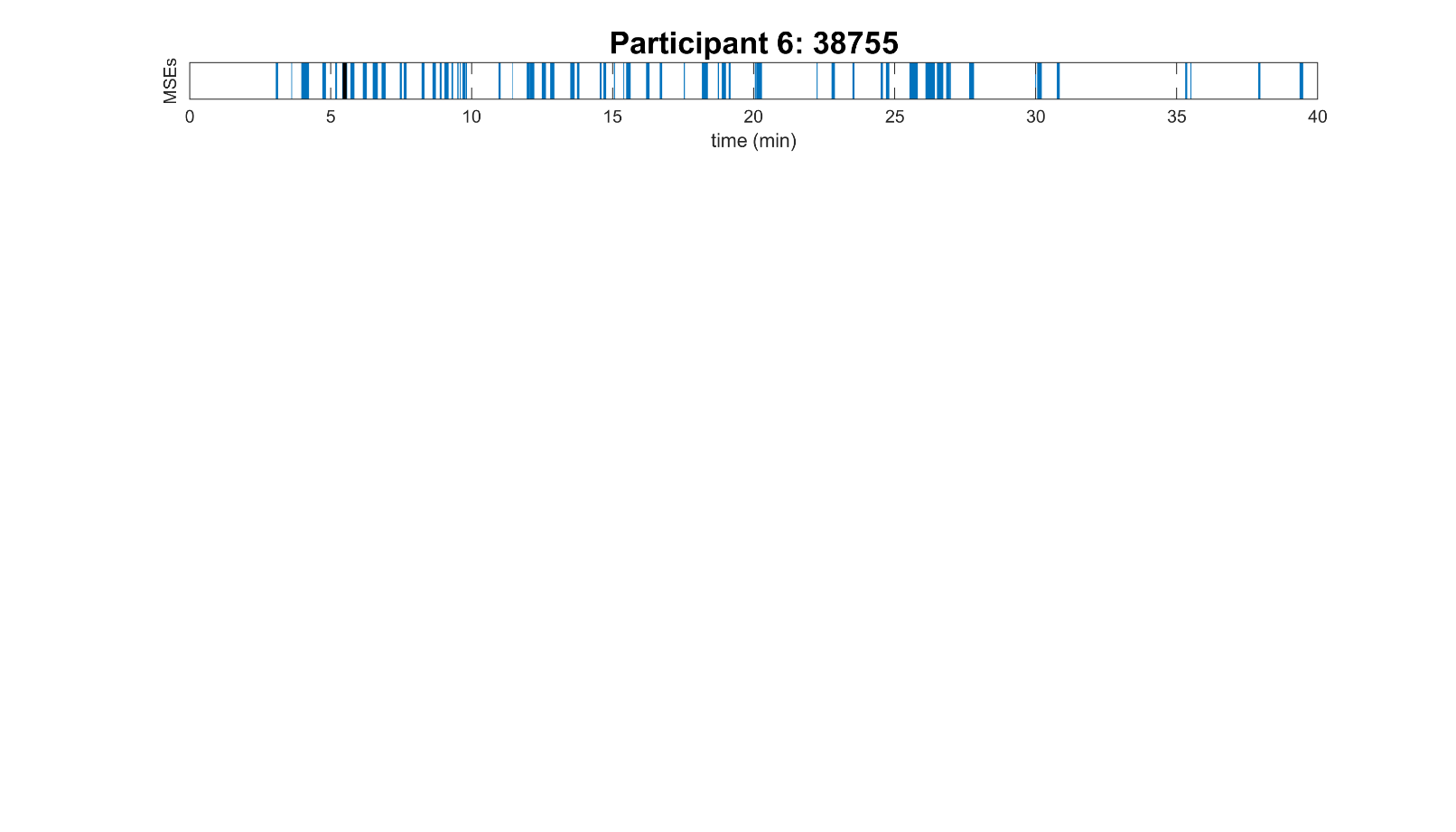

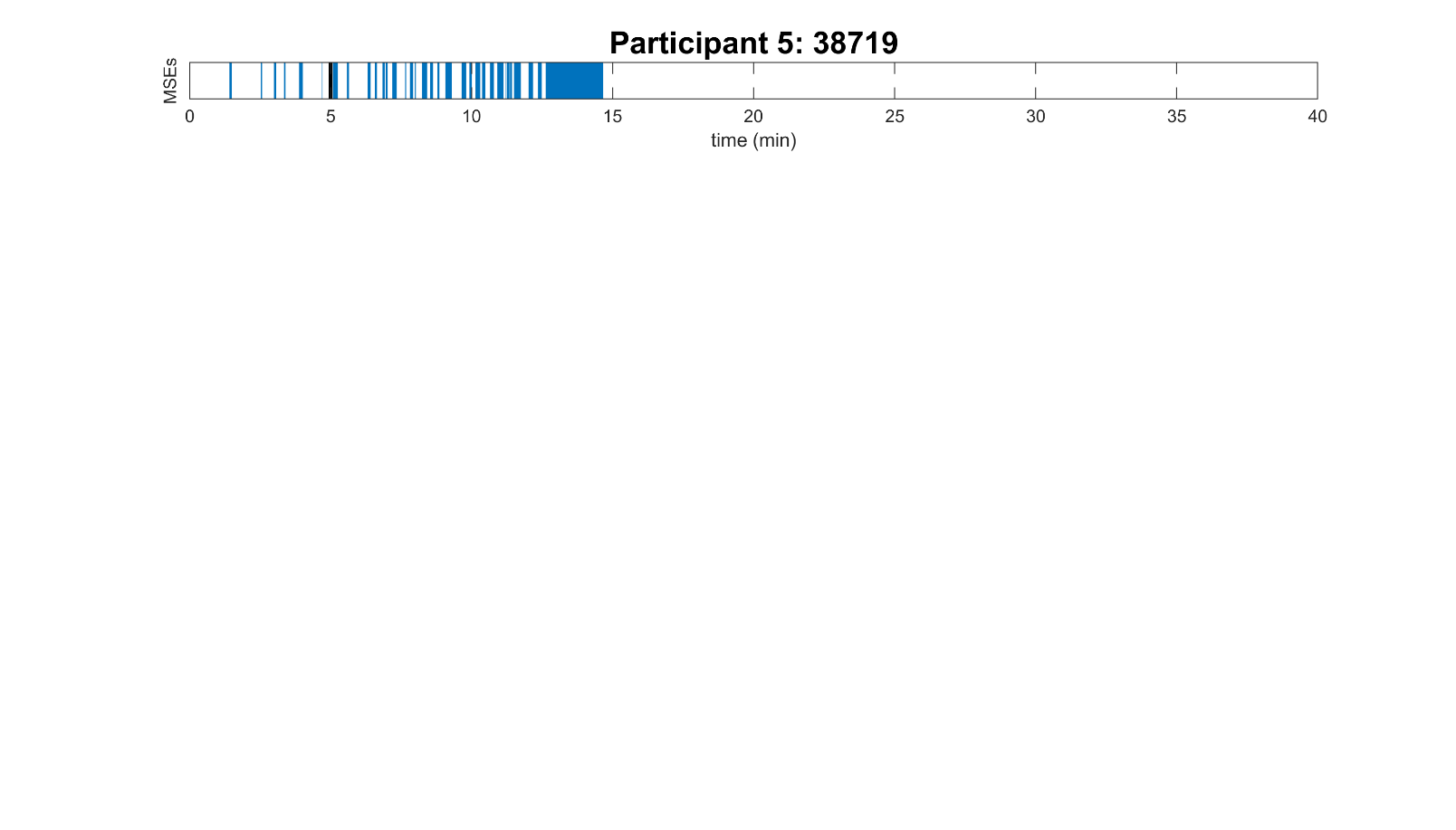

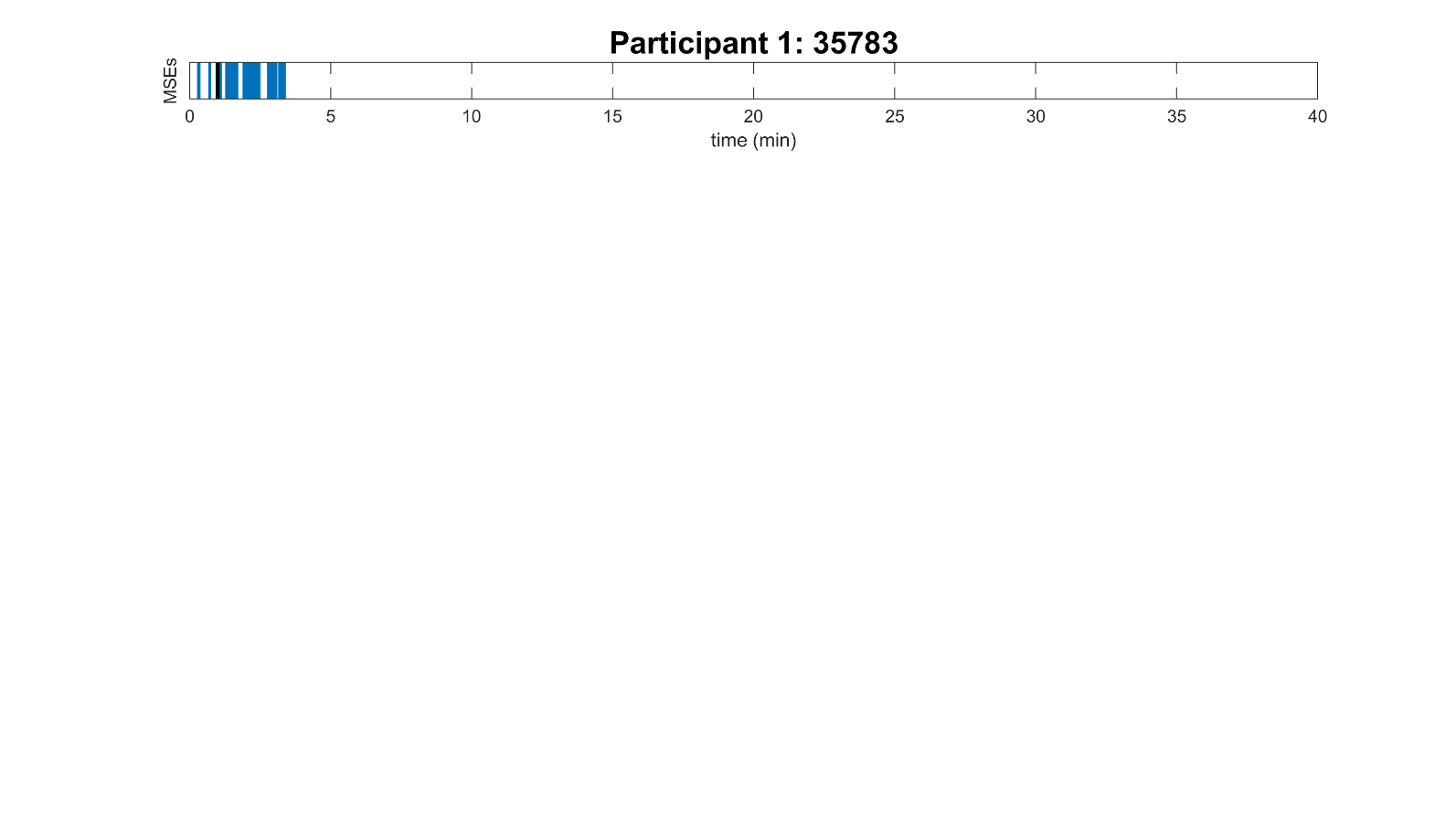

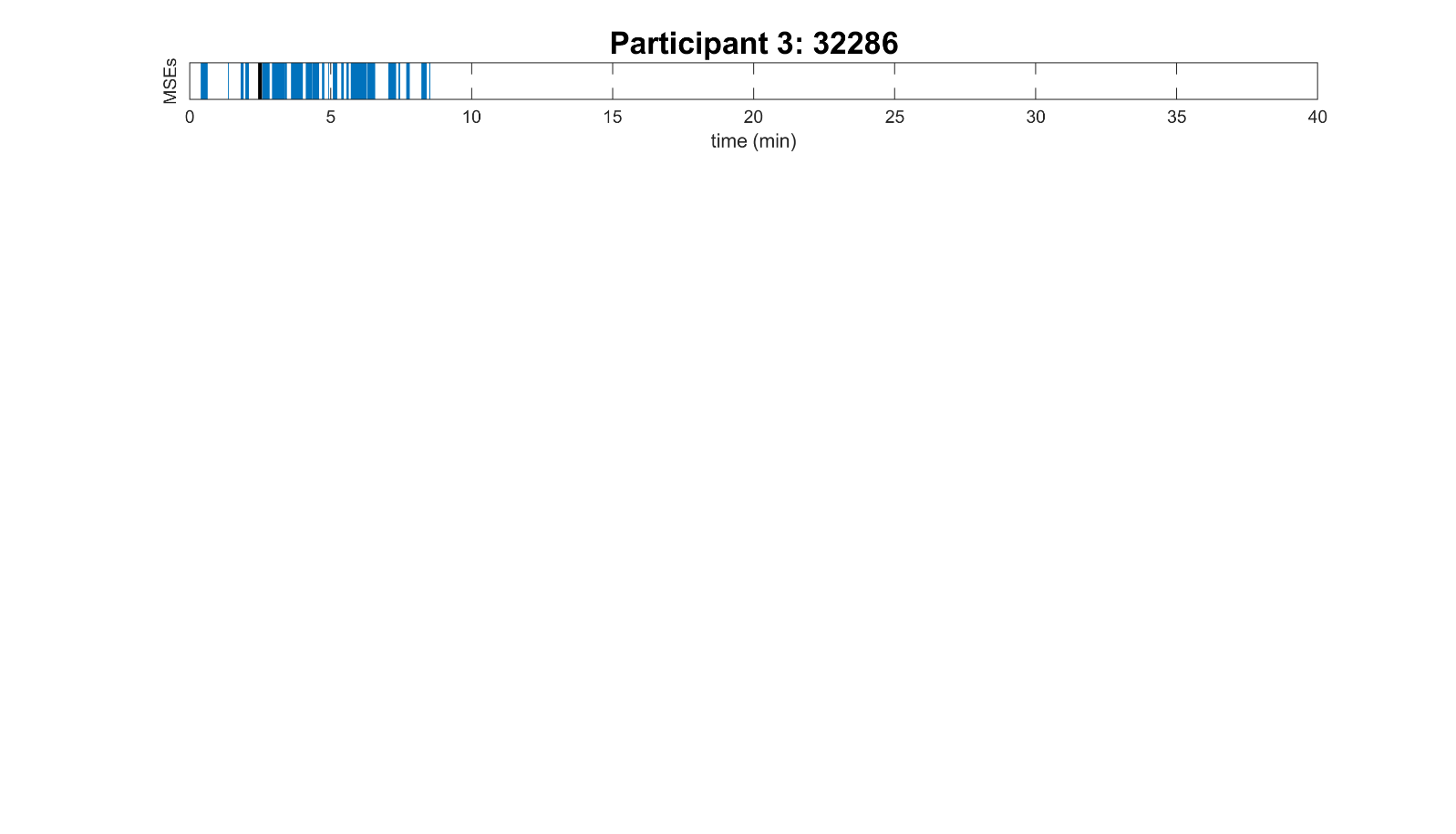

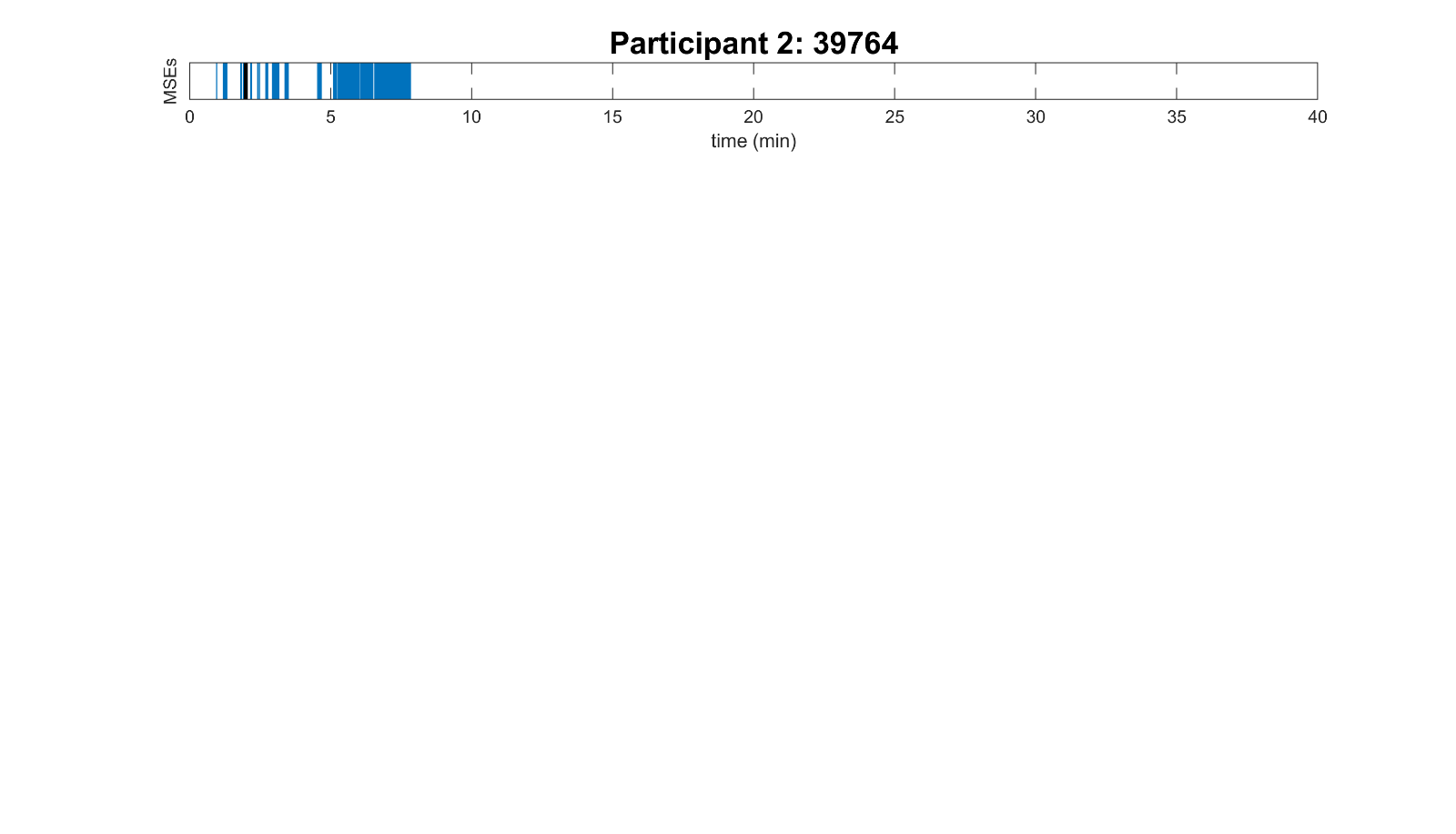
**Figure S1:** Automatic detection of microsleep episodes (MSEs, blue) in the maintenance of wakefulness test of all 18 participants after sleep deprivation. AASM-defined sleep onset is indicated (black). Participants are numbered according to Figure 3A.

**
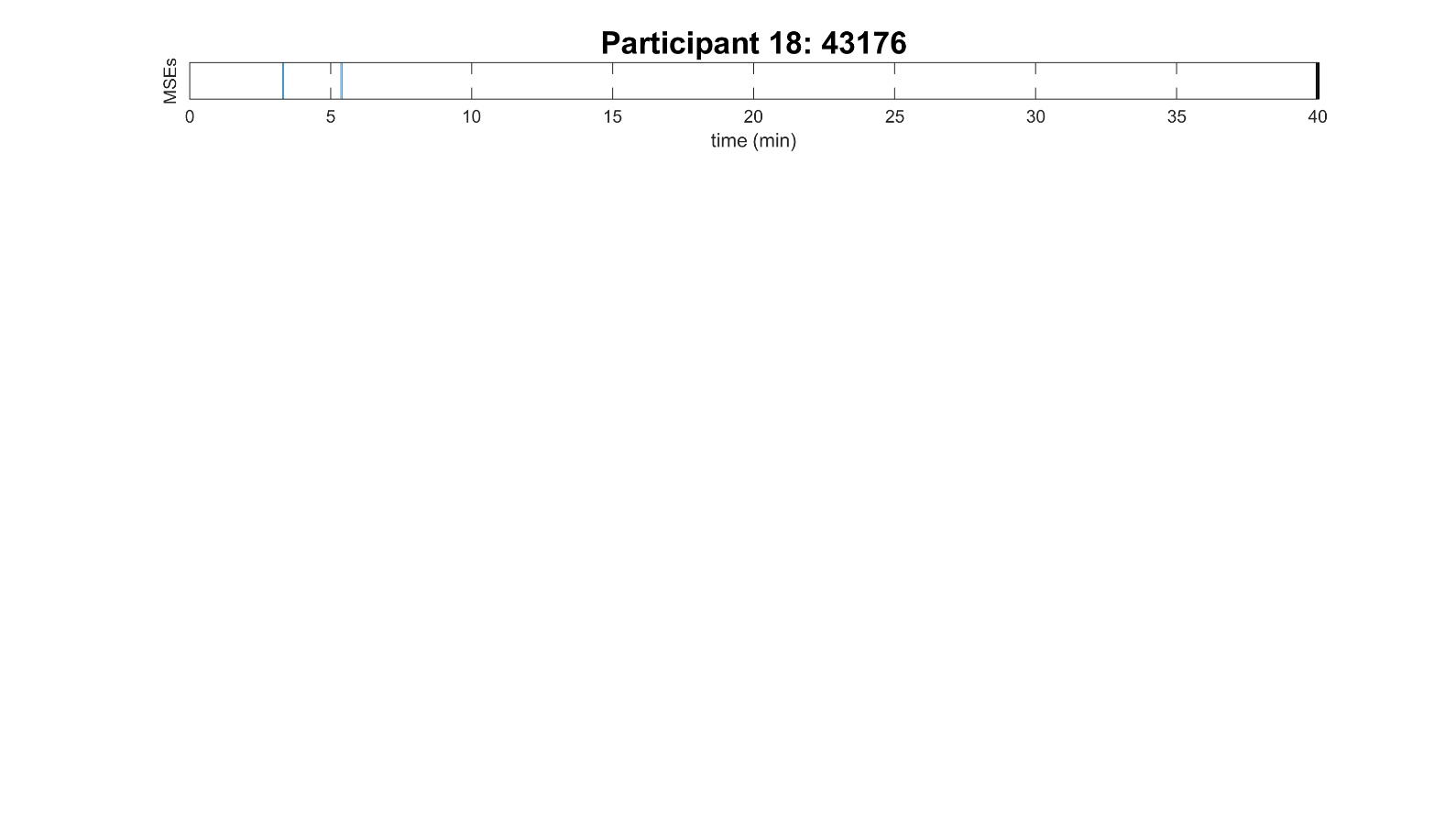

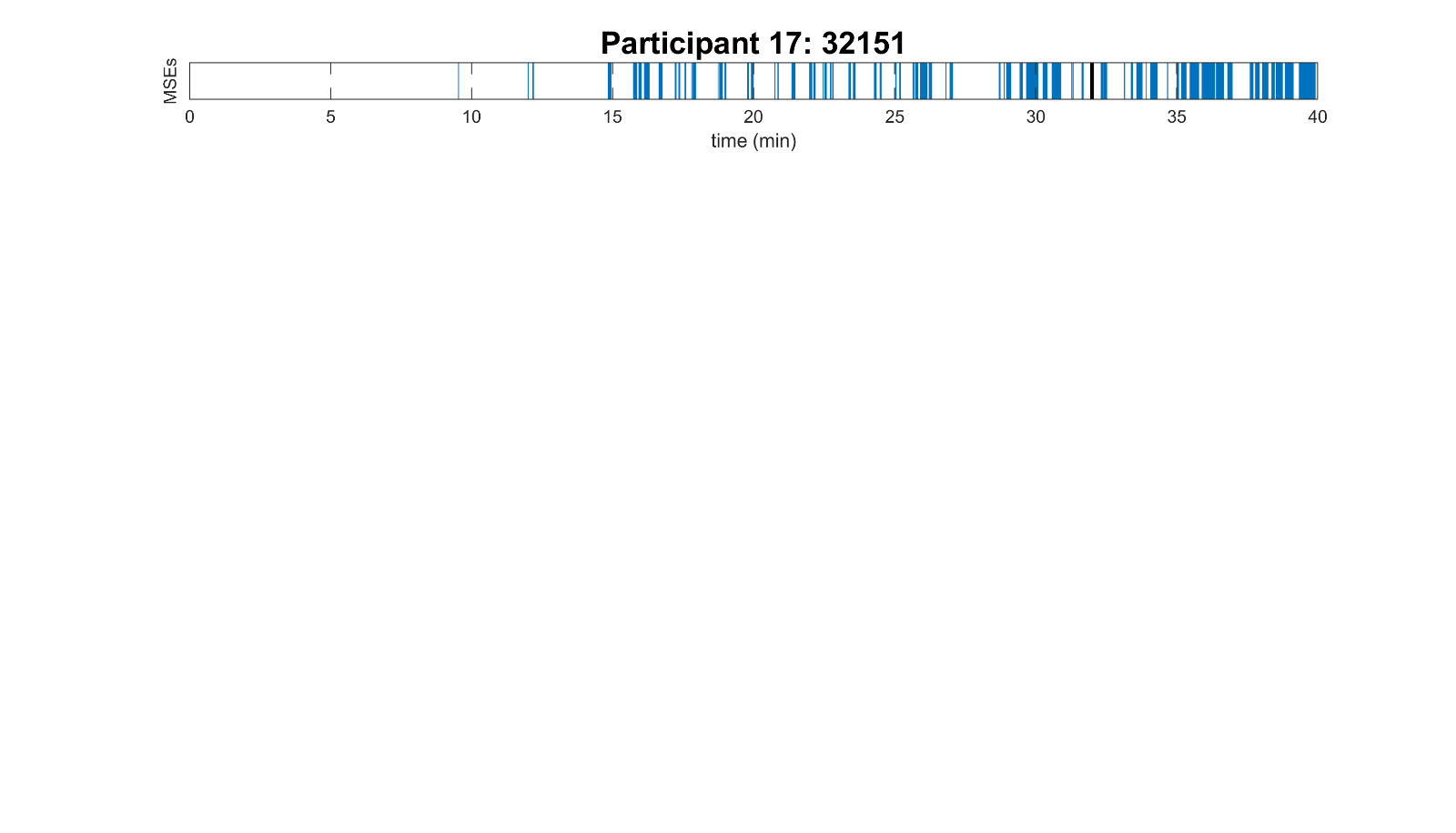

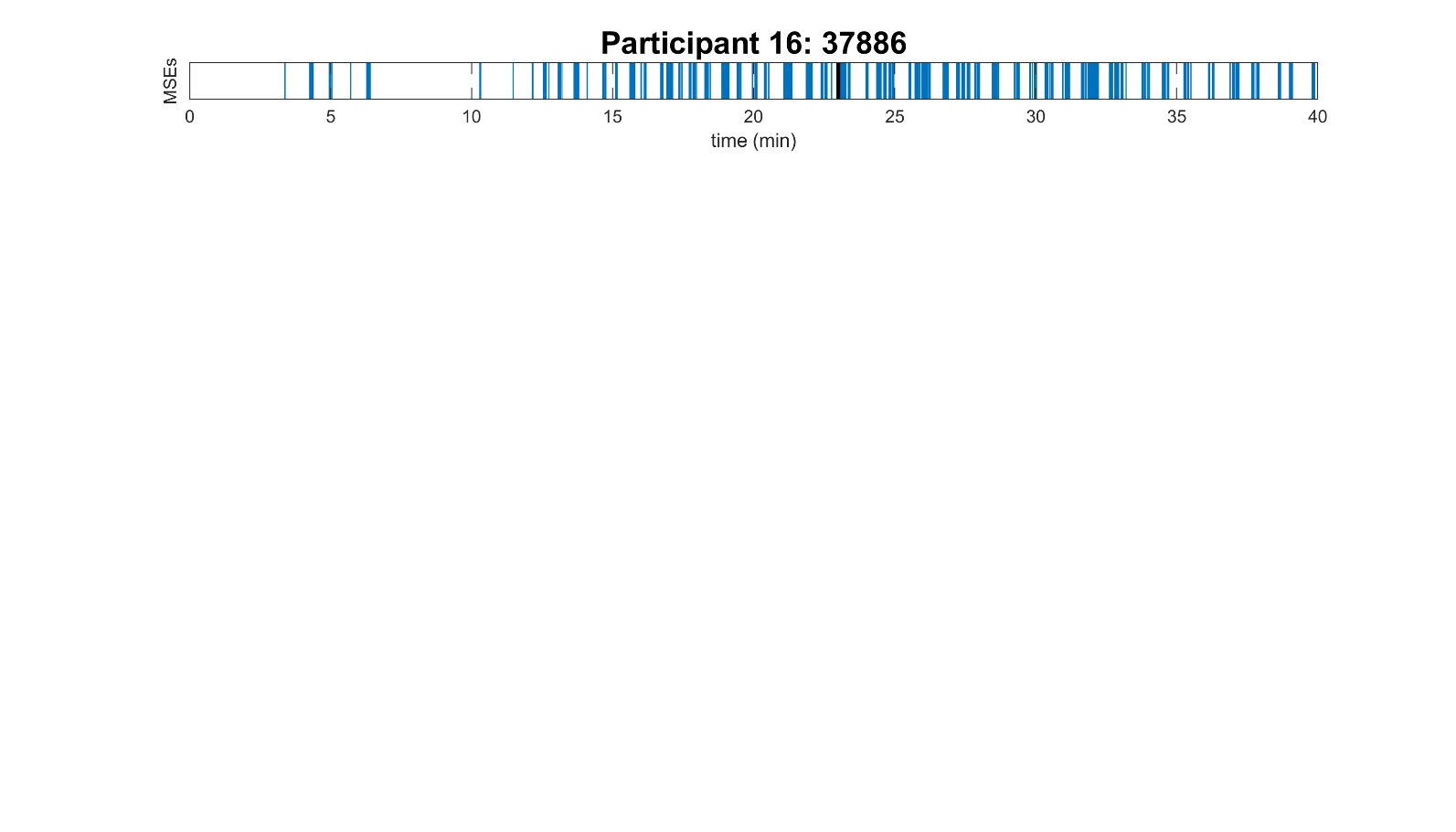

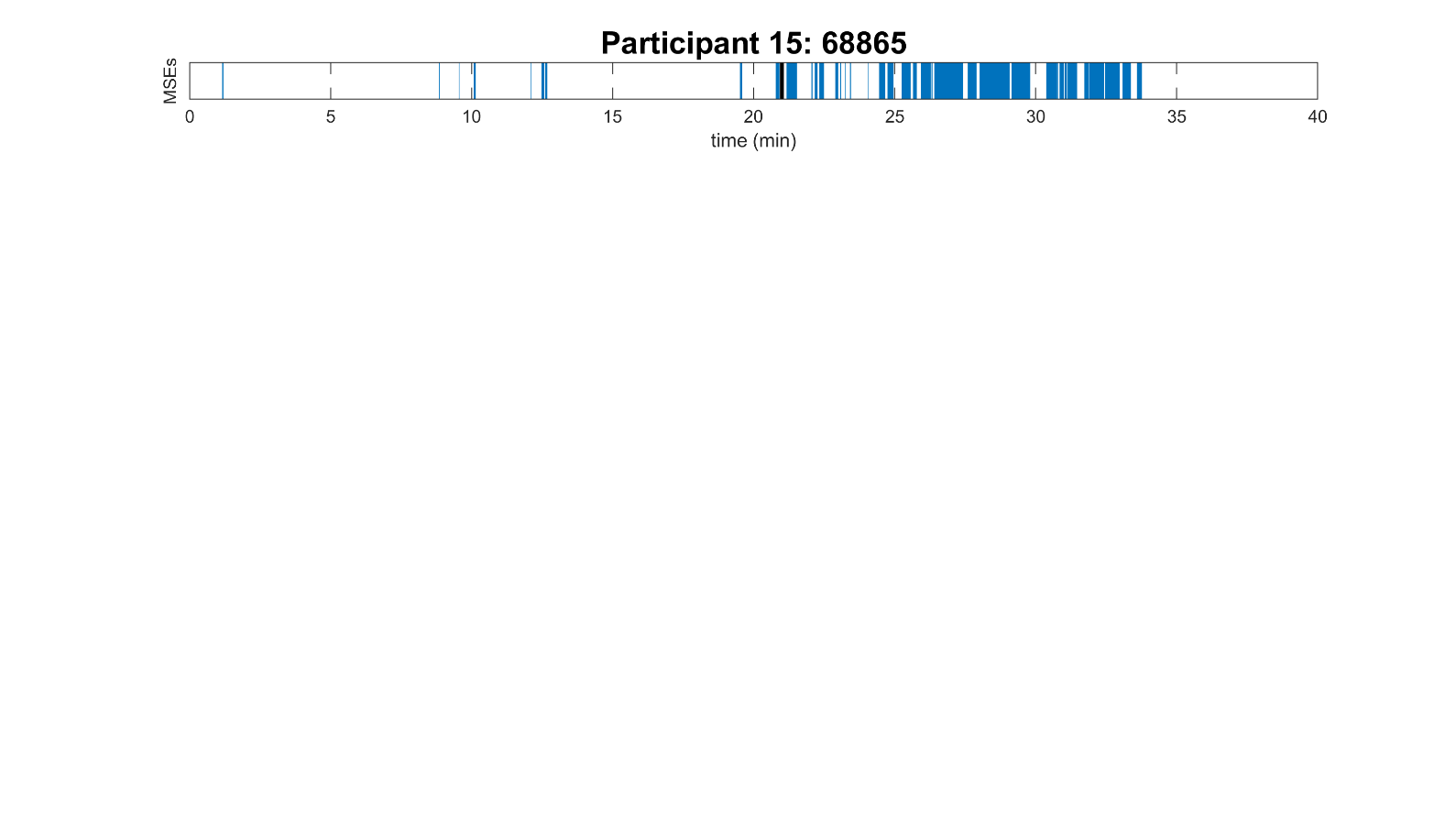

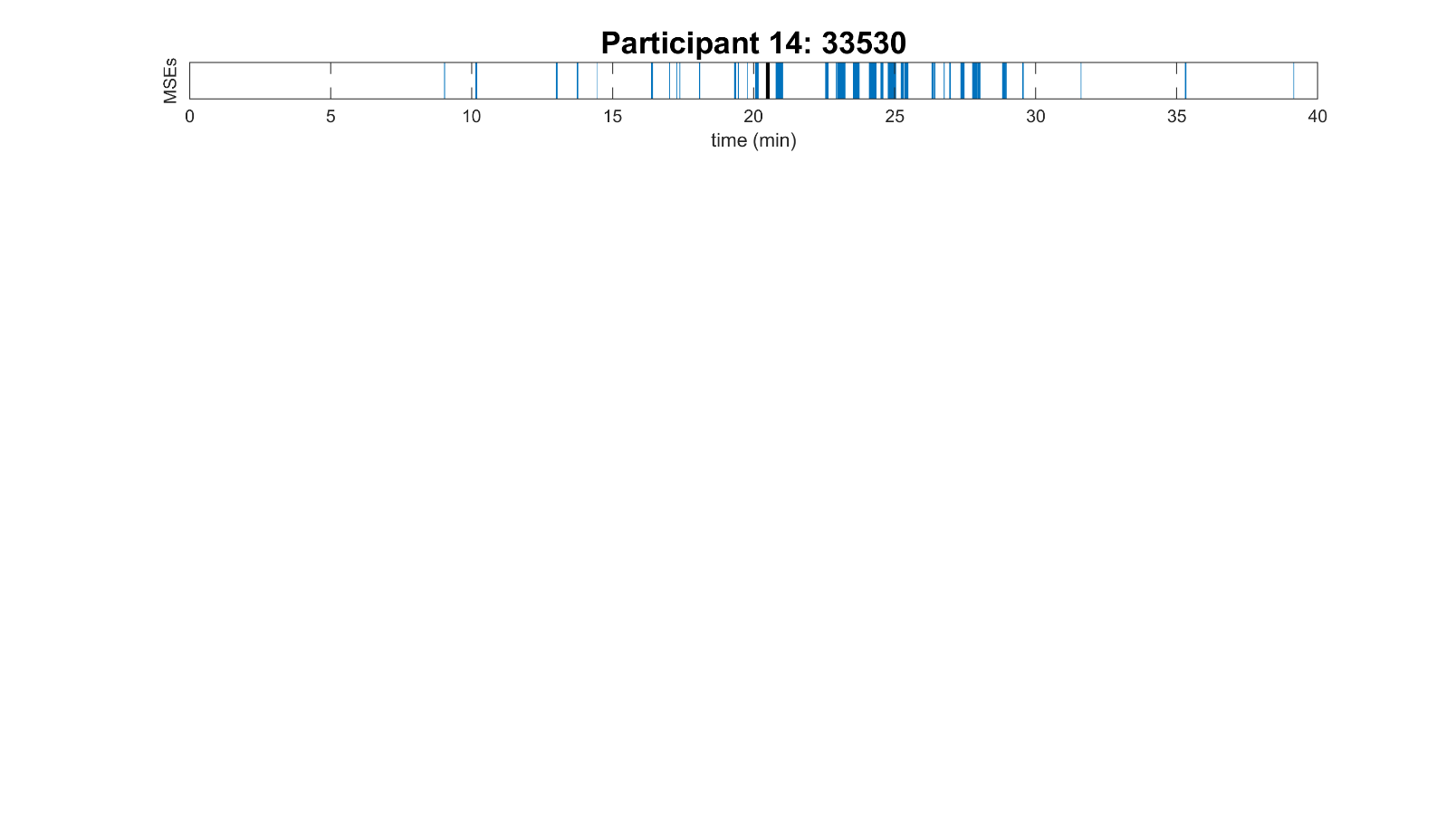

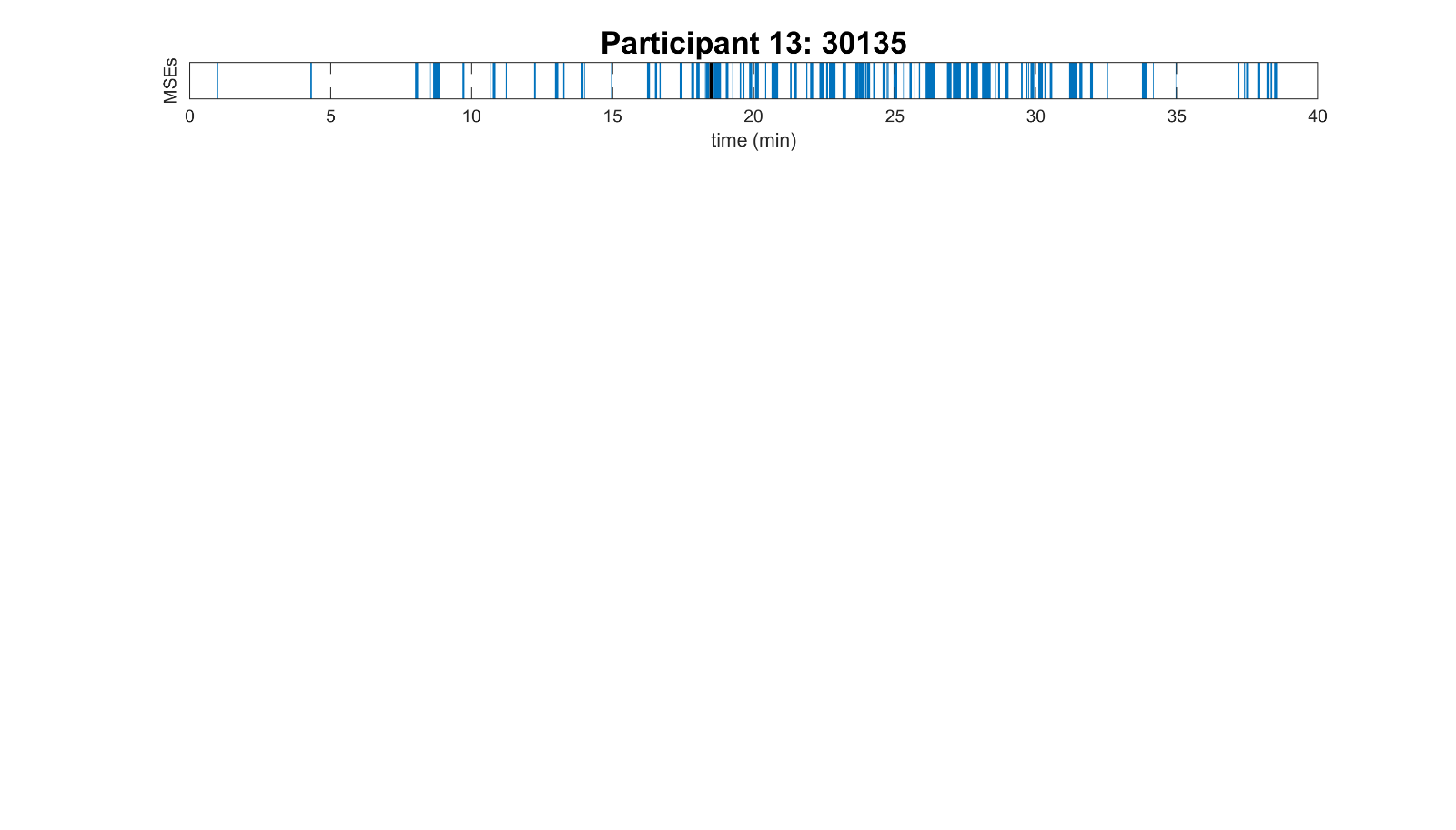

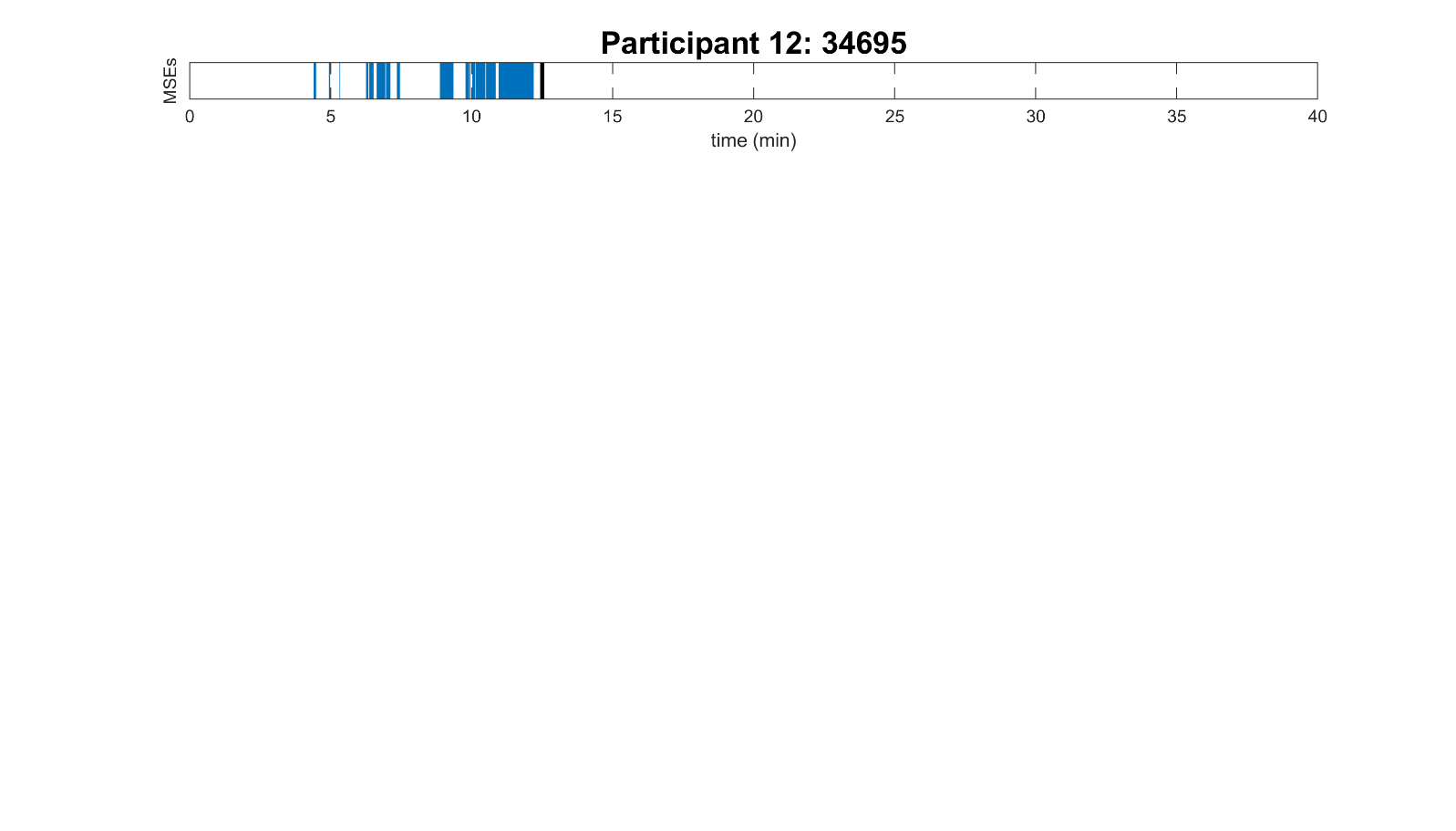

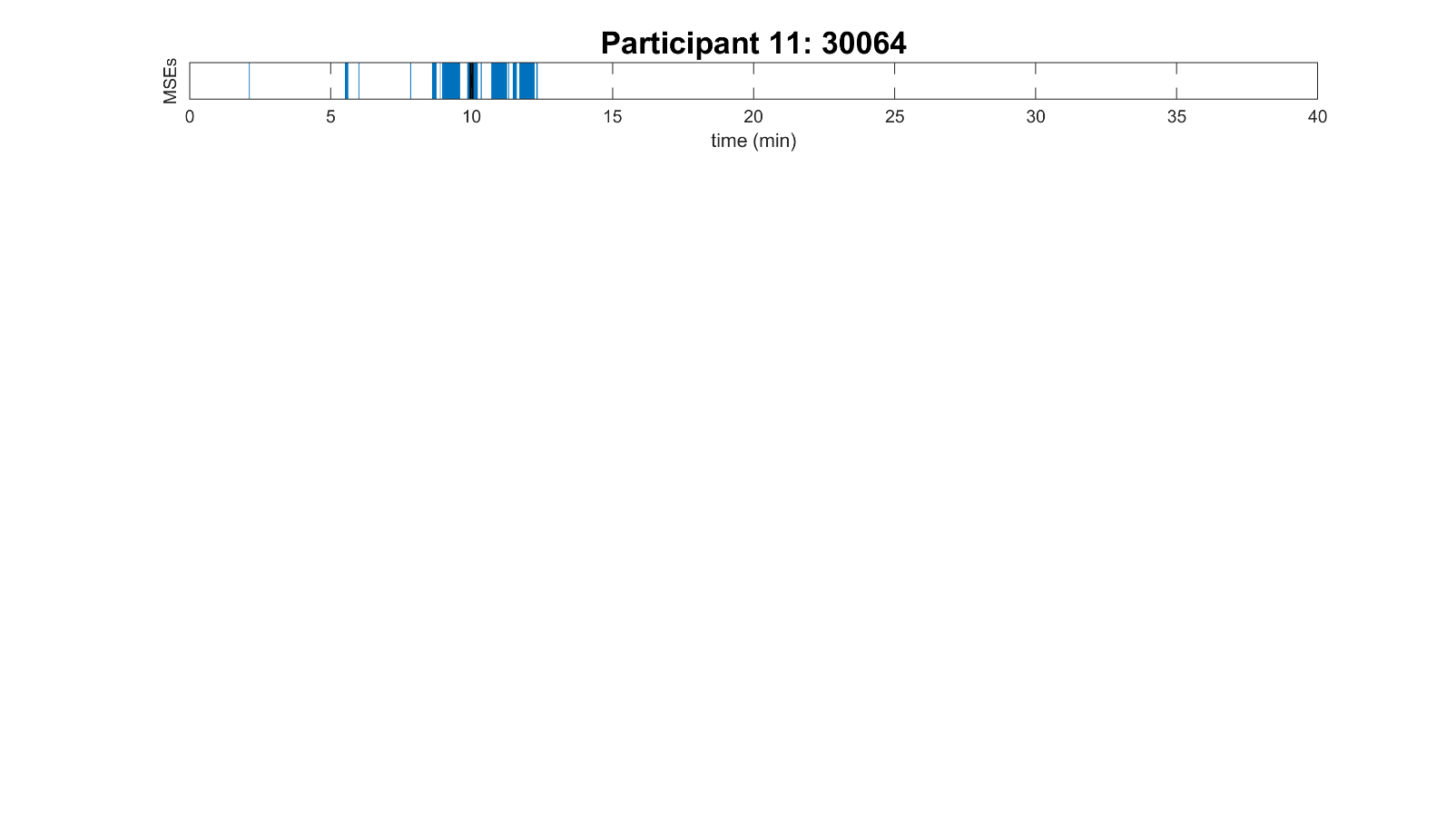
**
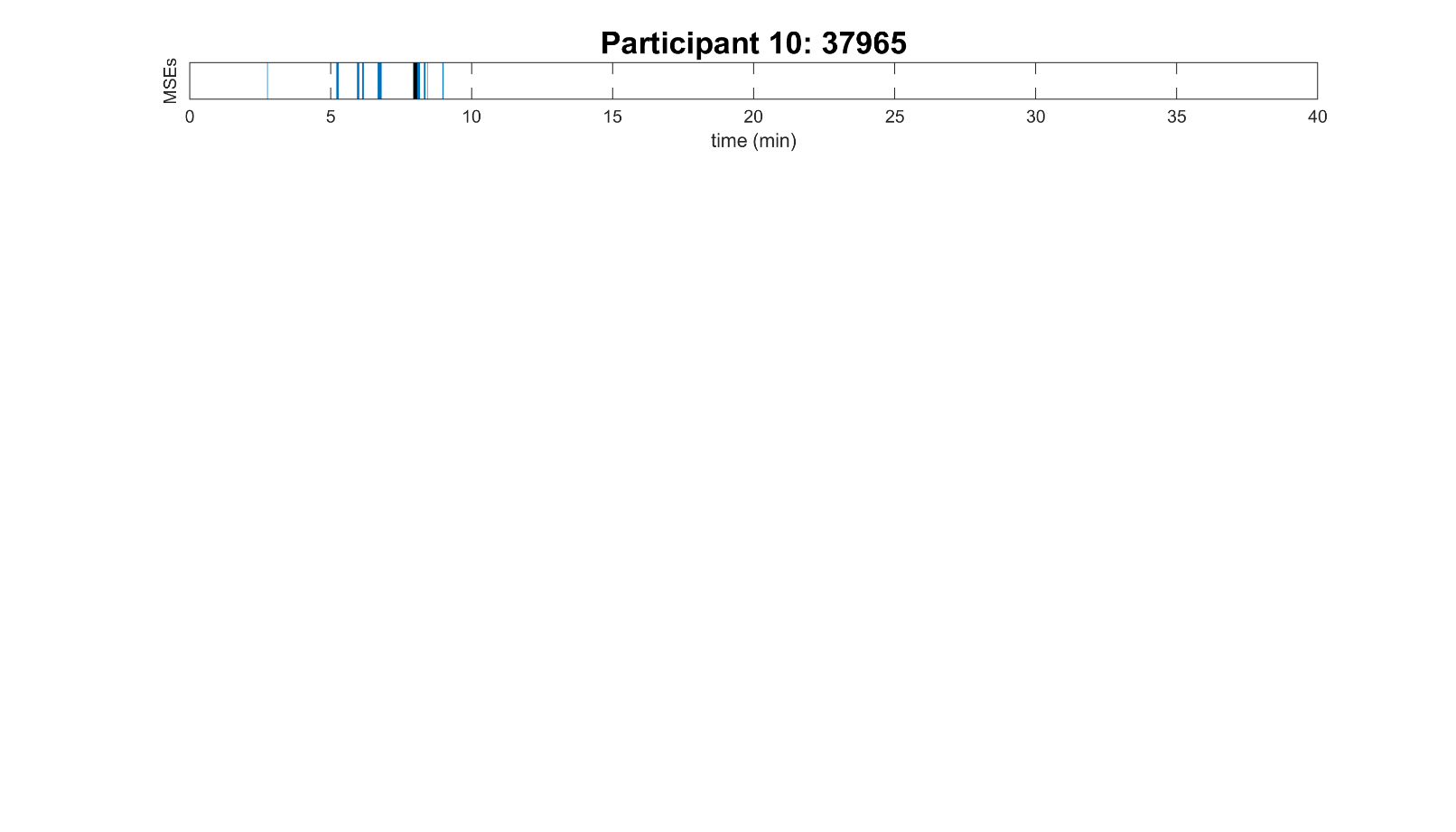

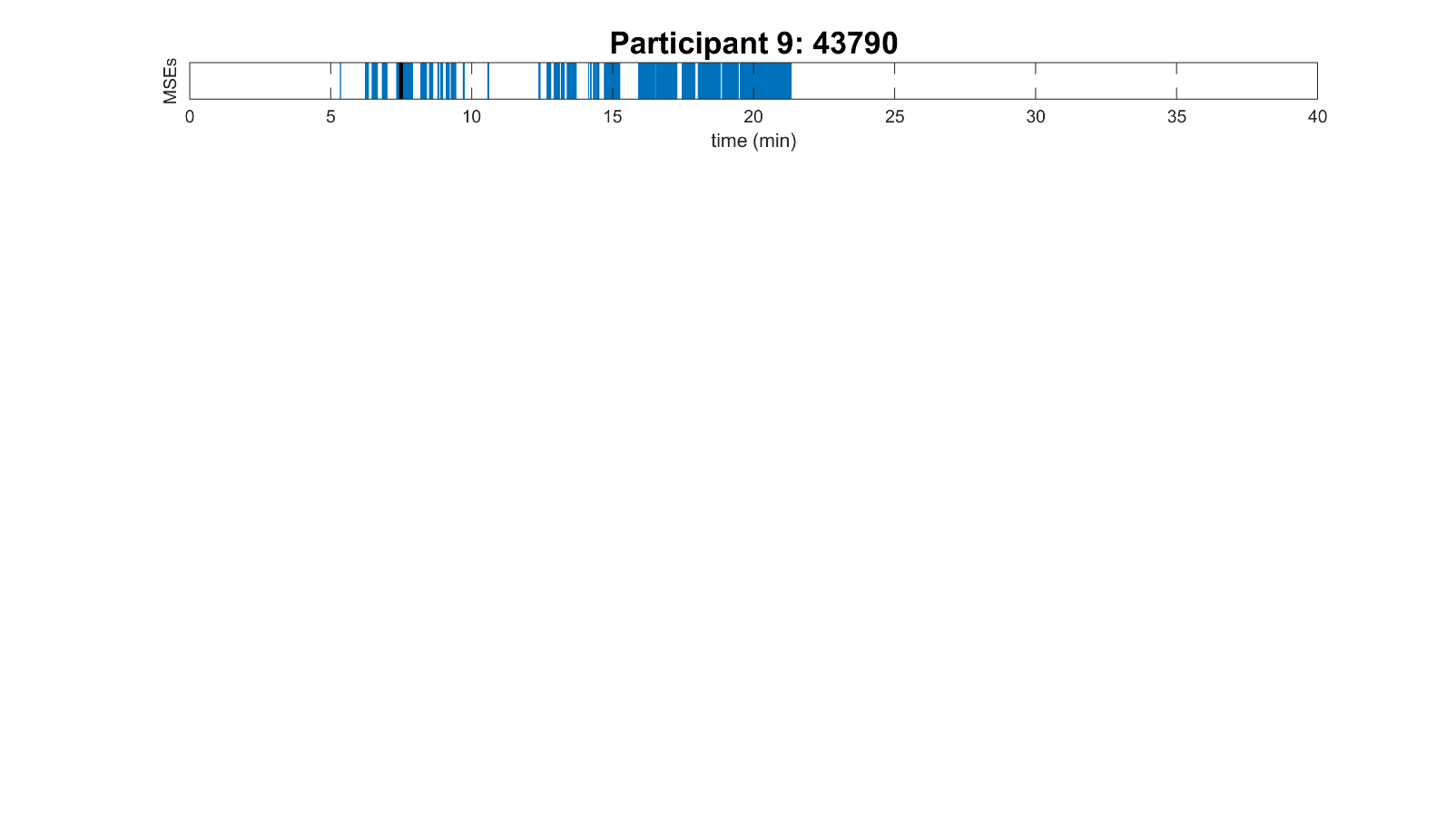


## **Figure S2**: Automatic detection of microsleep episodes in the driving simulator and driving performance of all 18 participants after sleep deprivation. For further details, see Figure 2A. Participants are numbered according to Figure 3A.


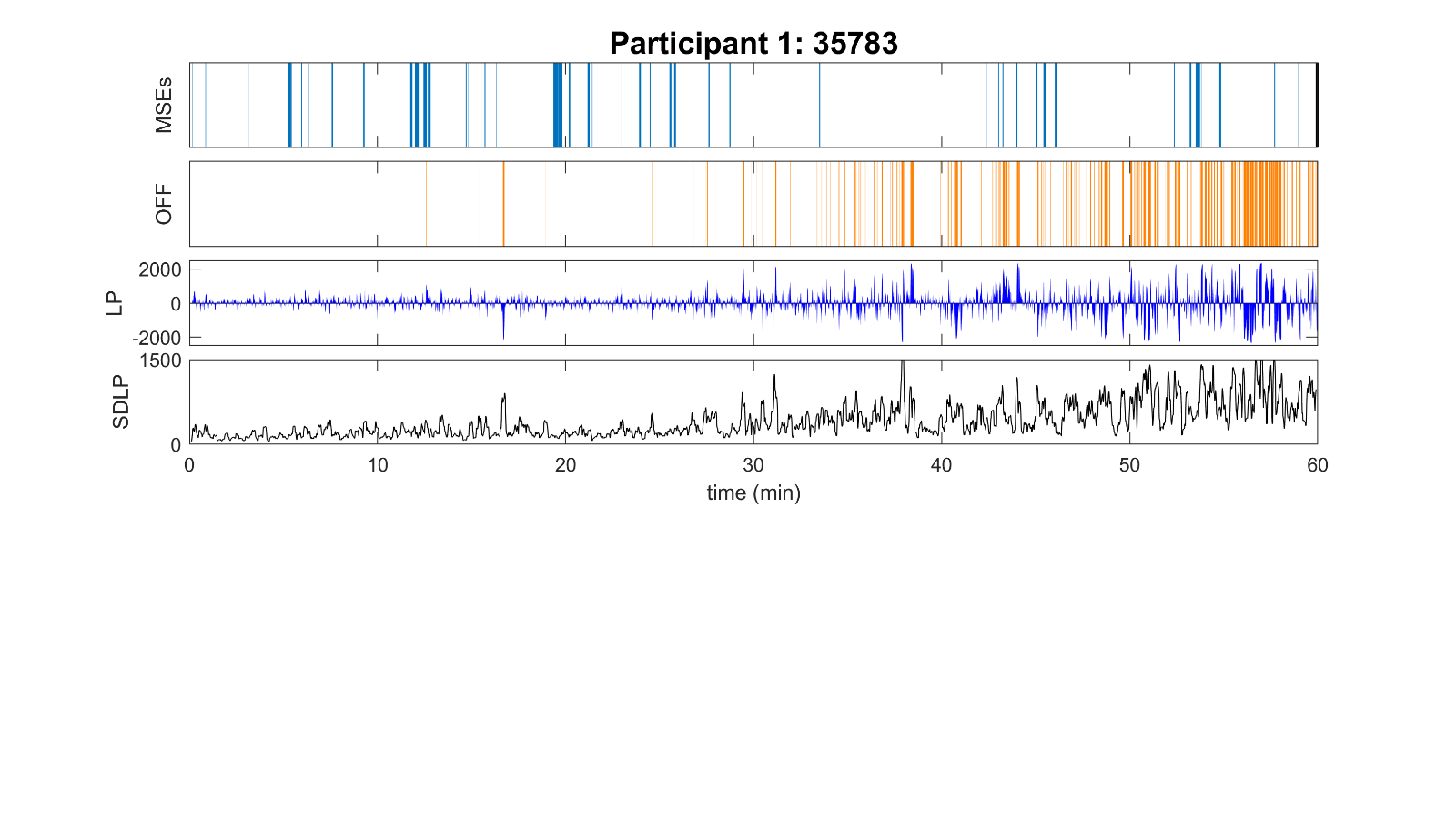


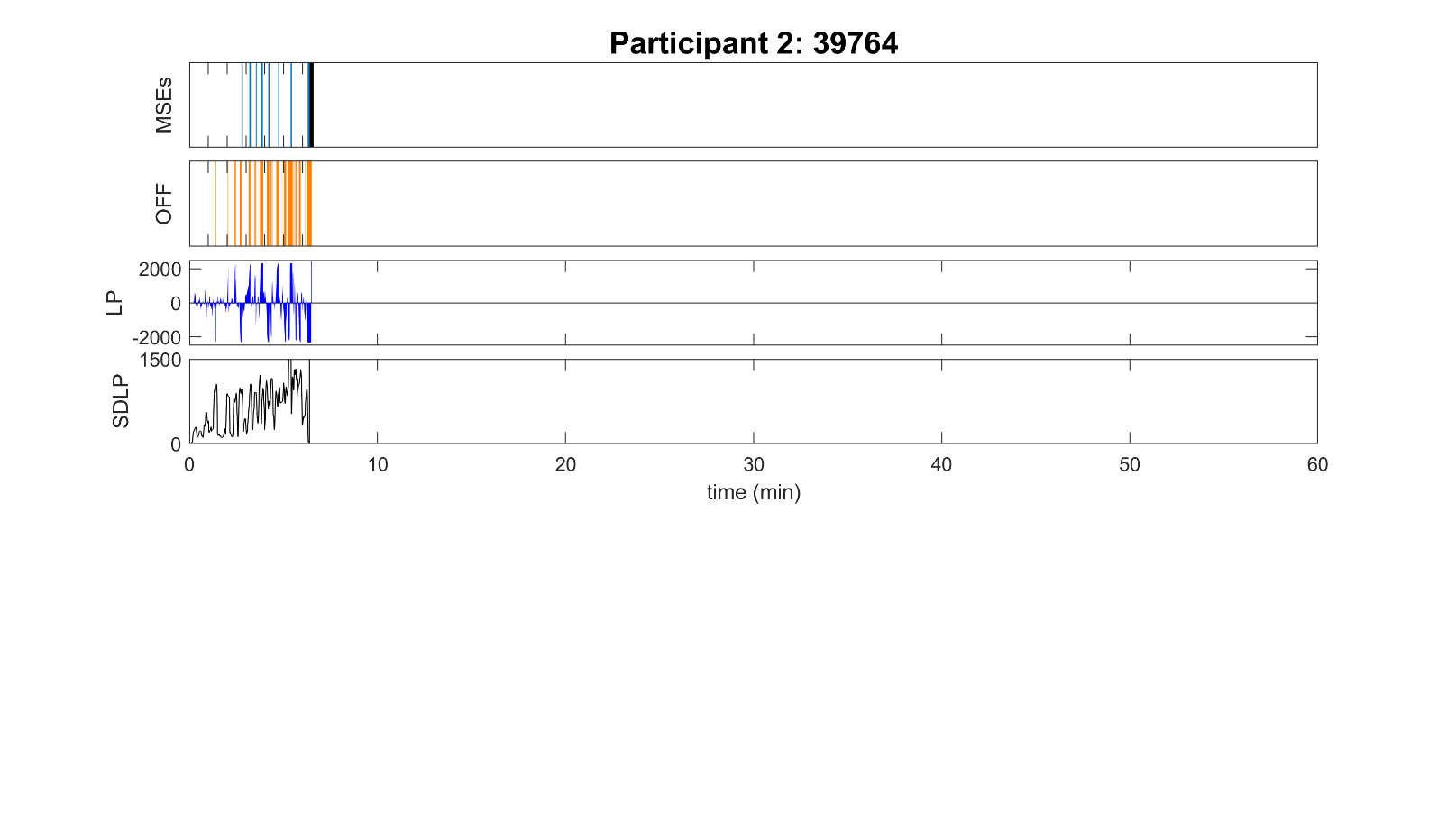


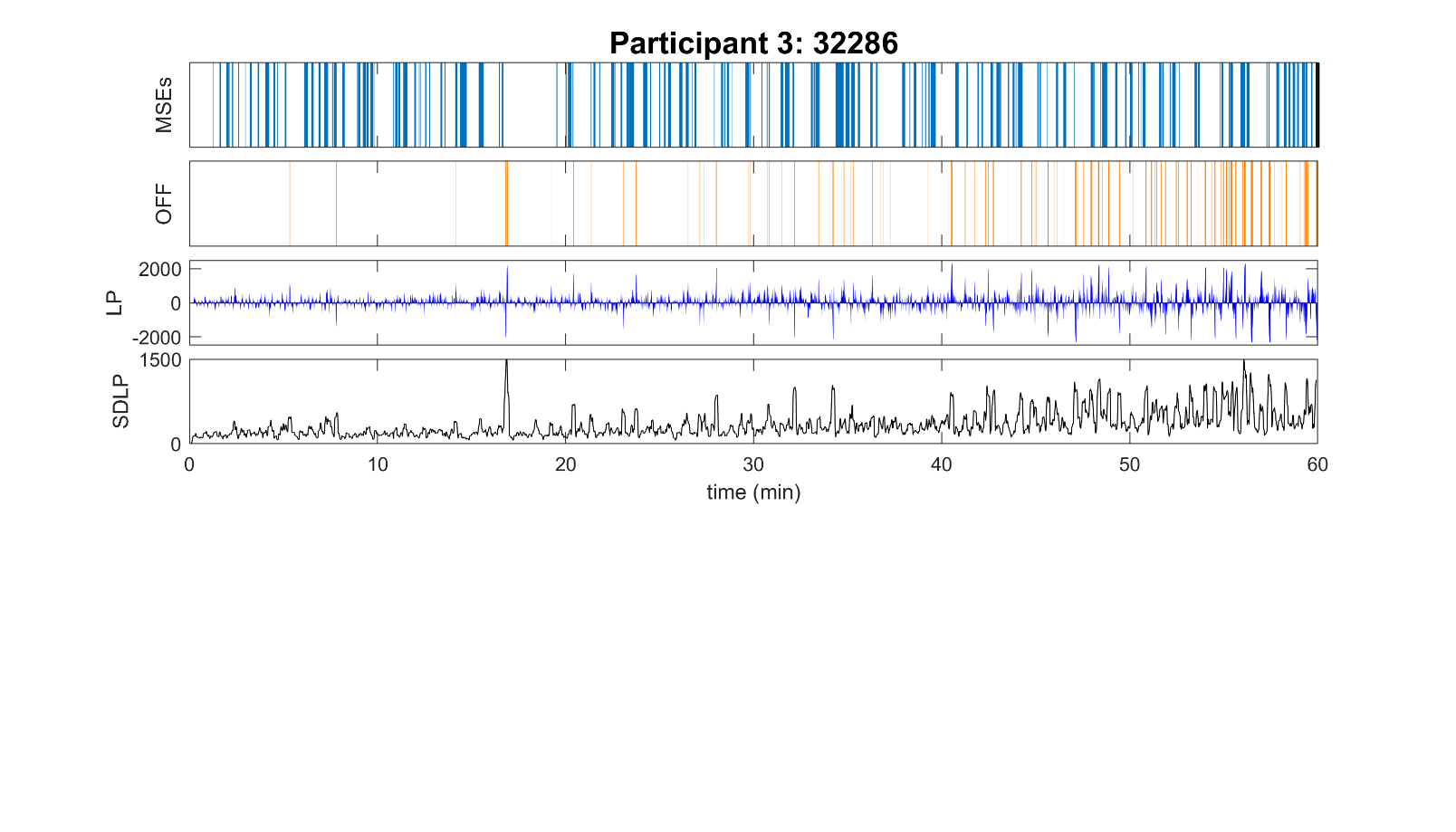


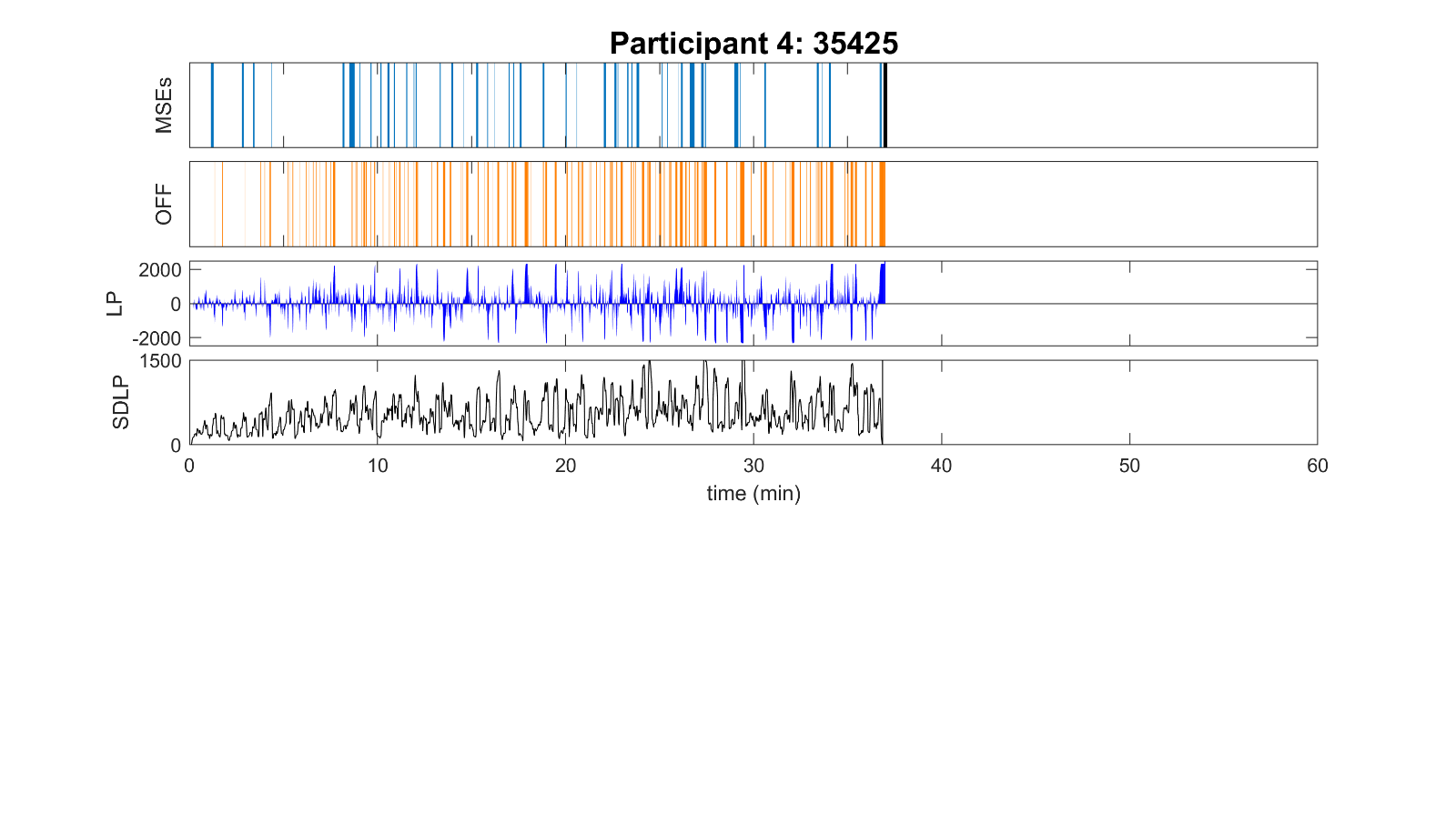


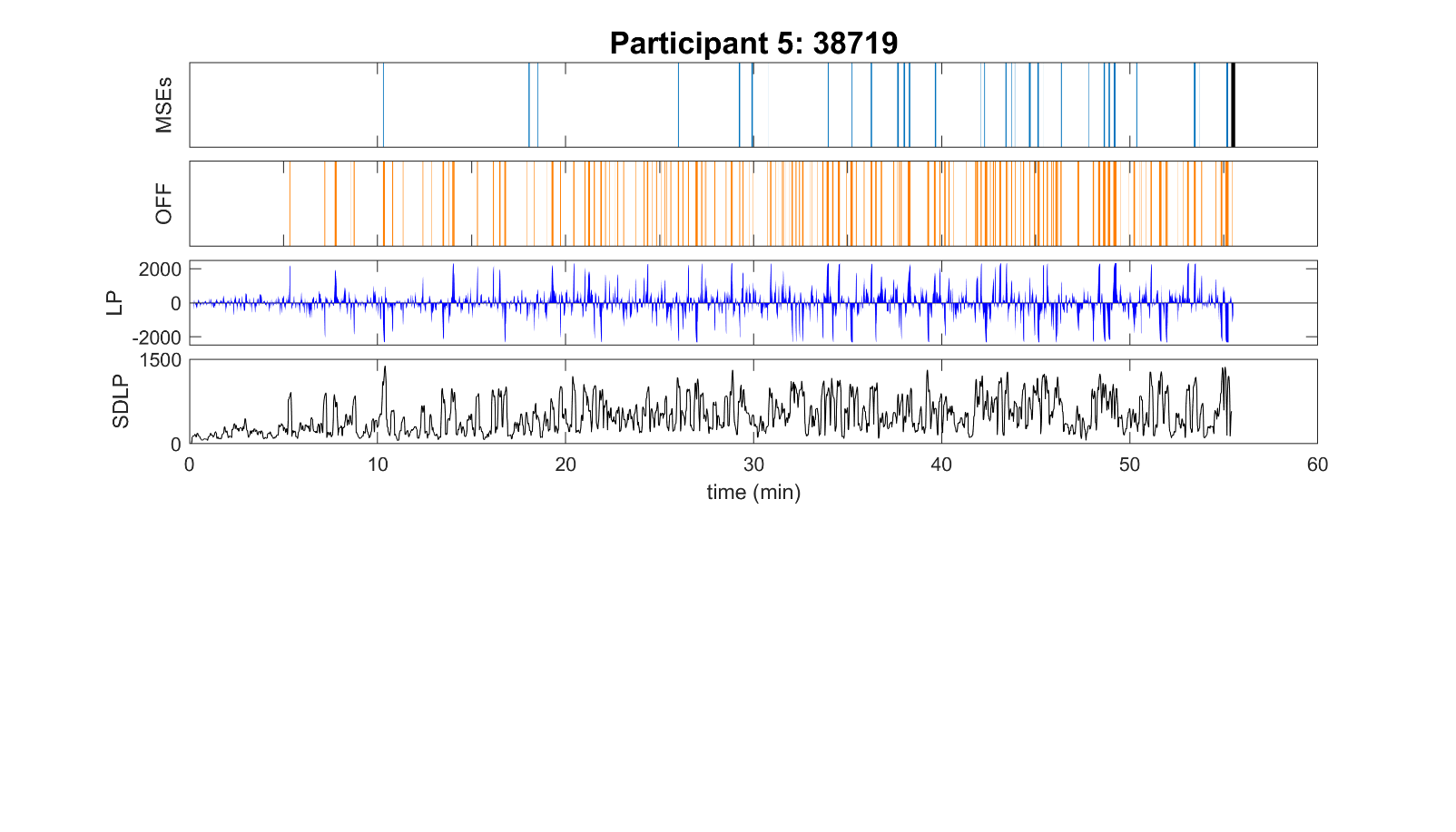


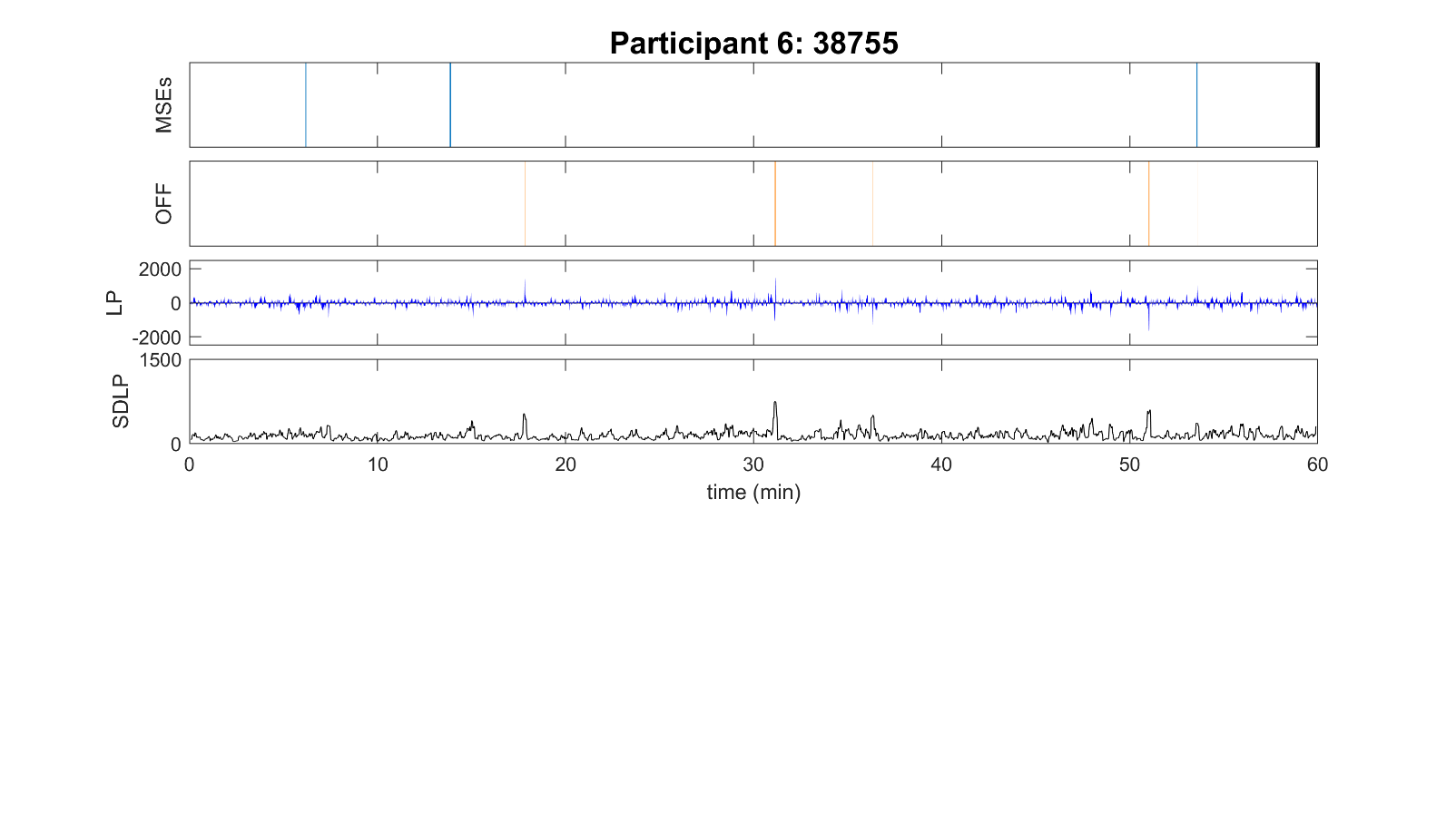


.
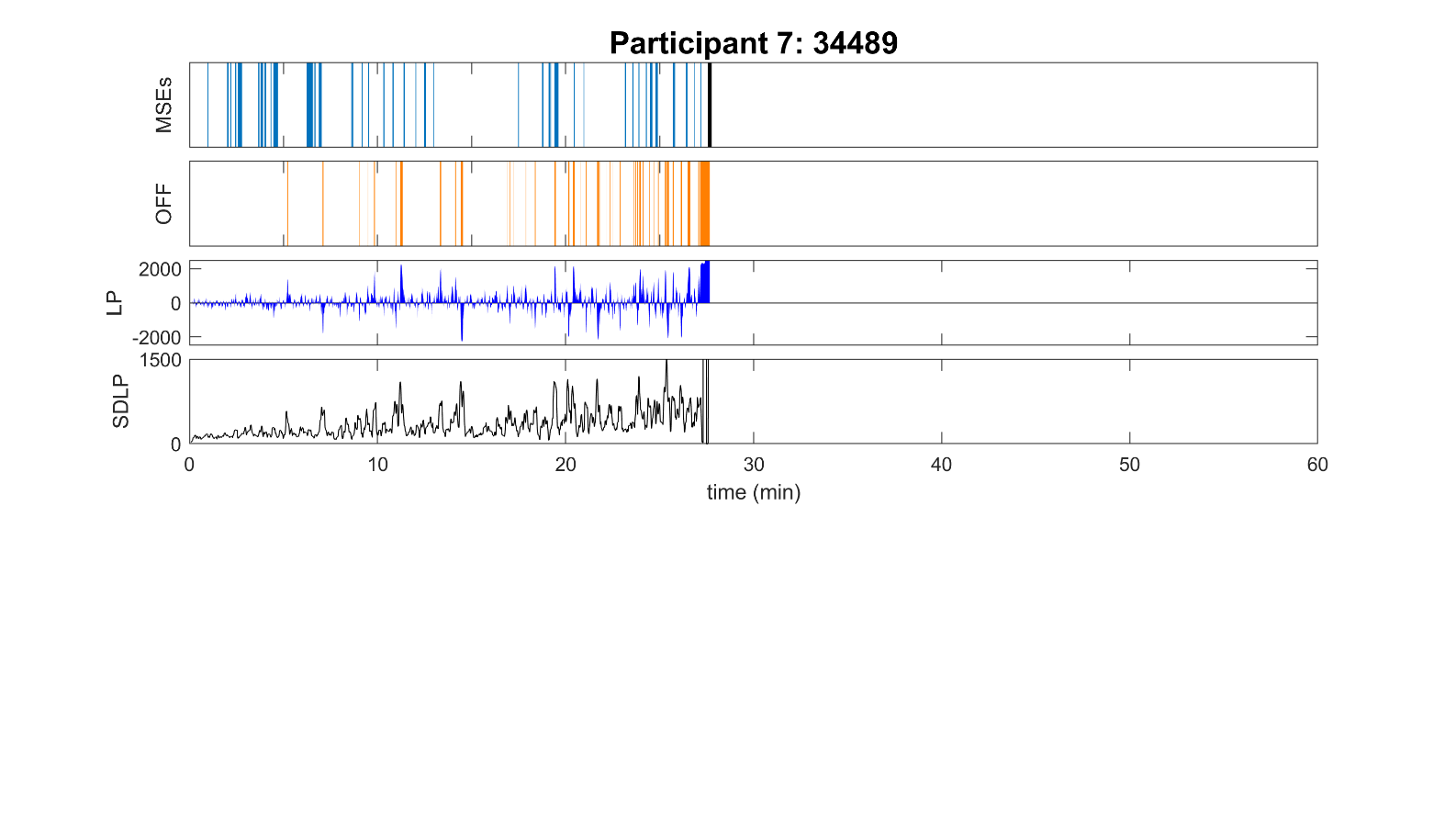


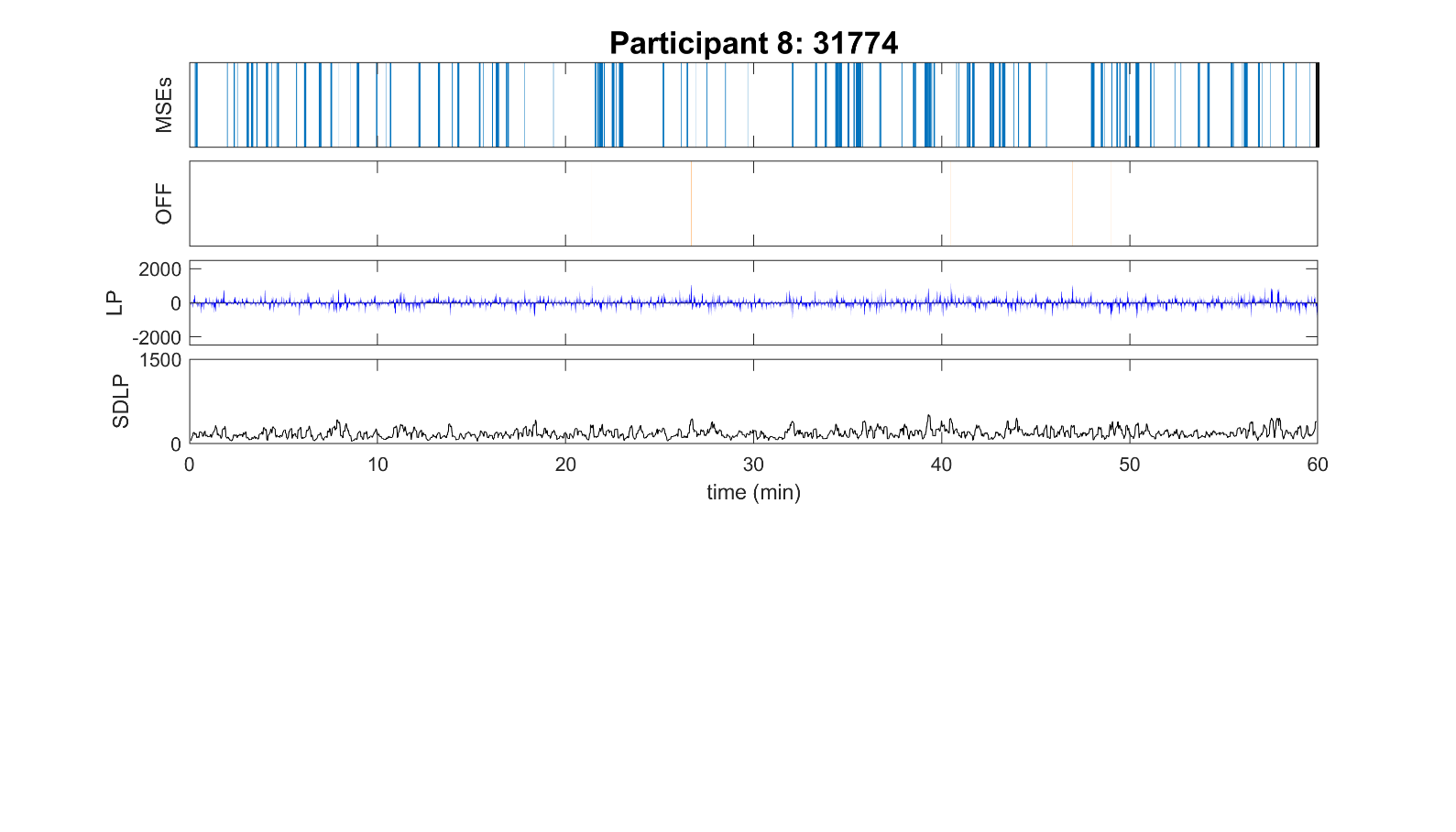


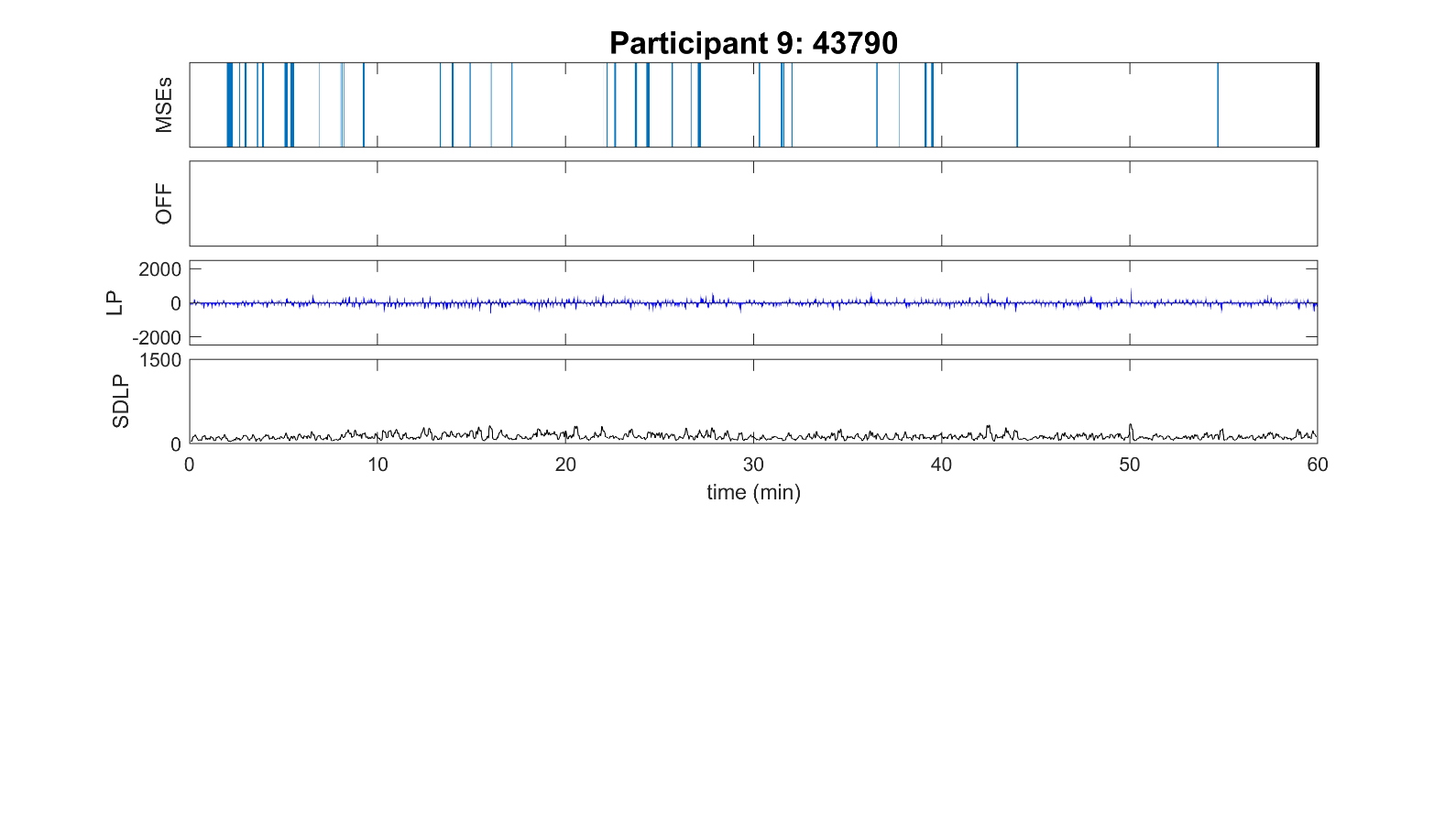


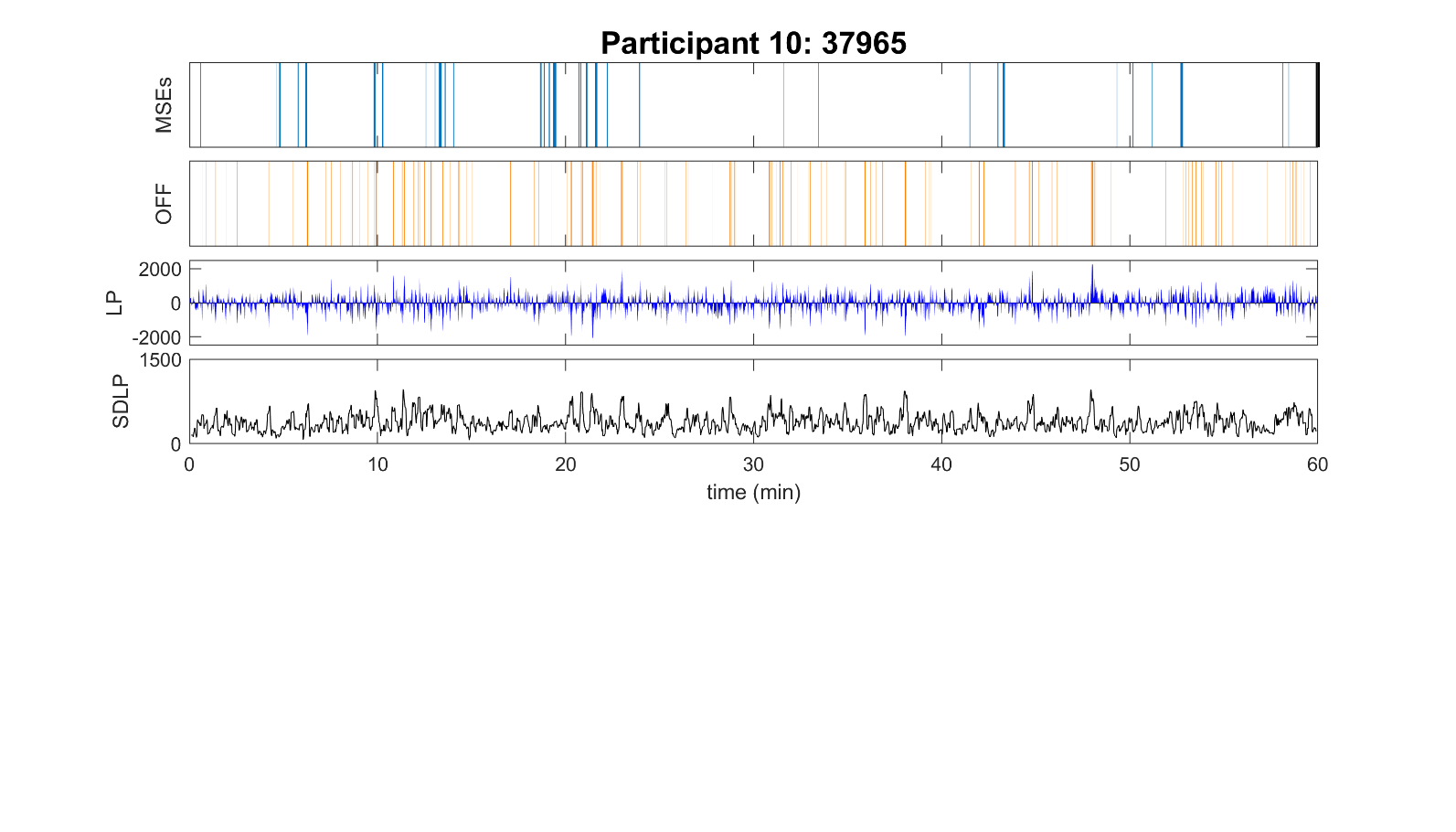


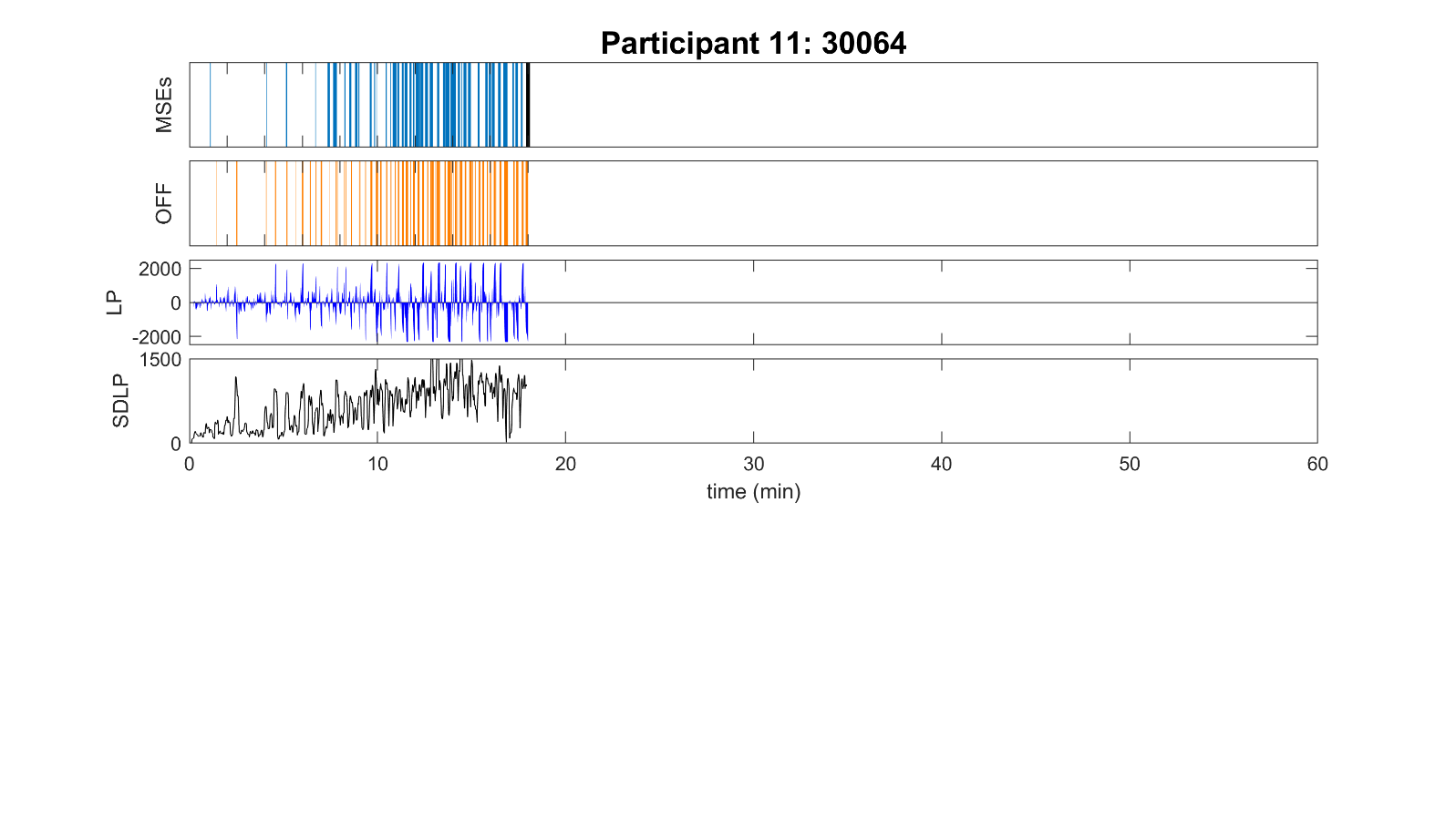


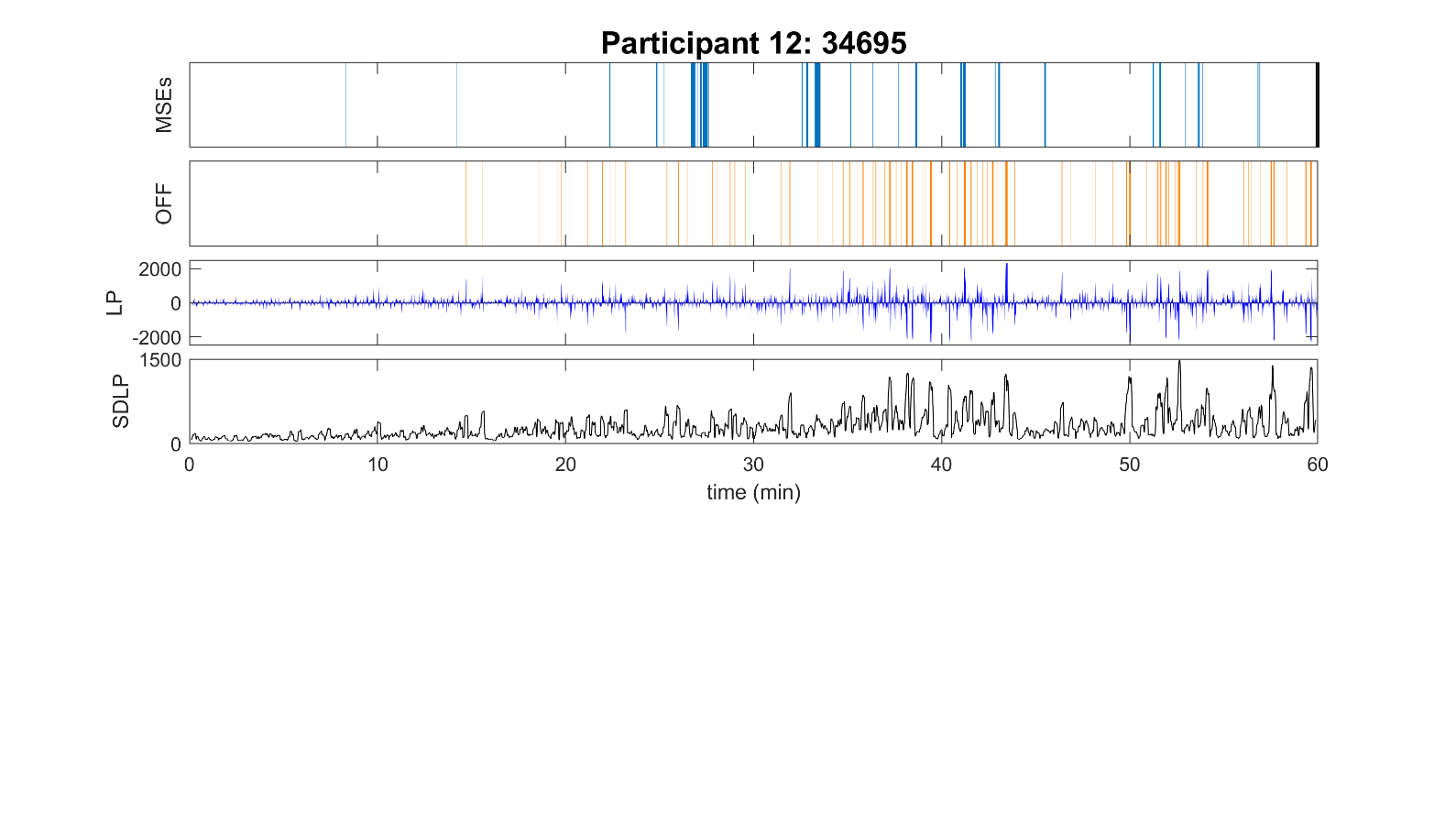


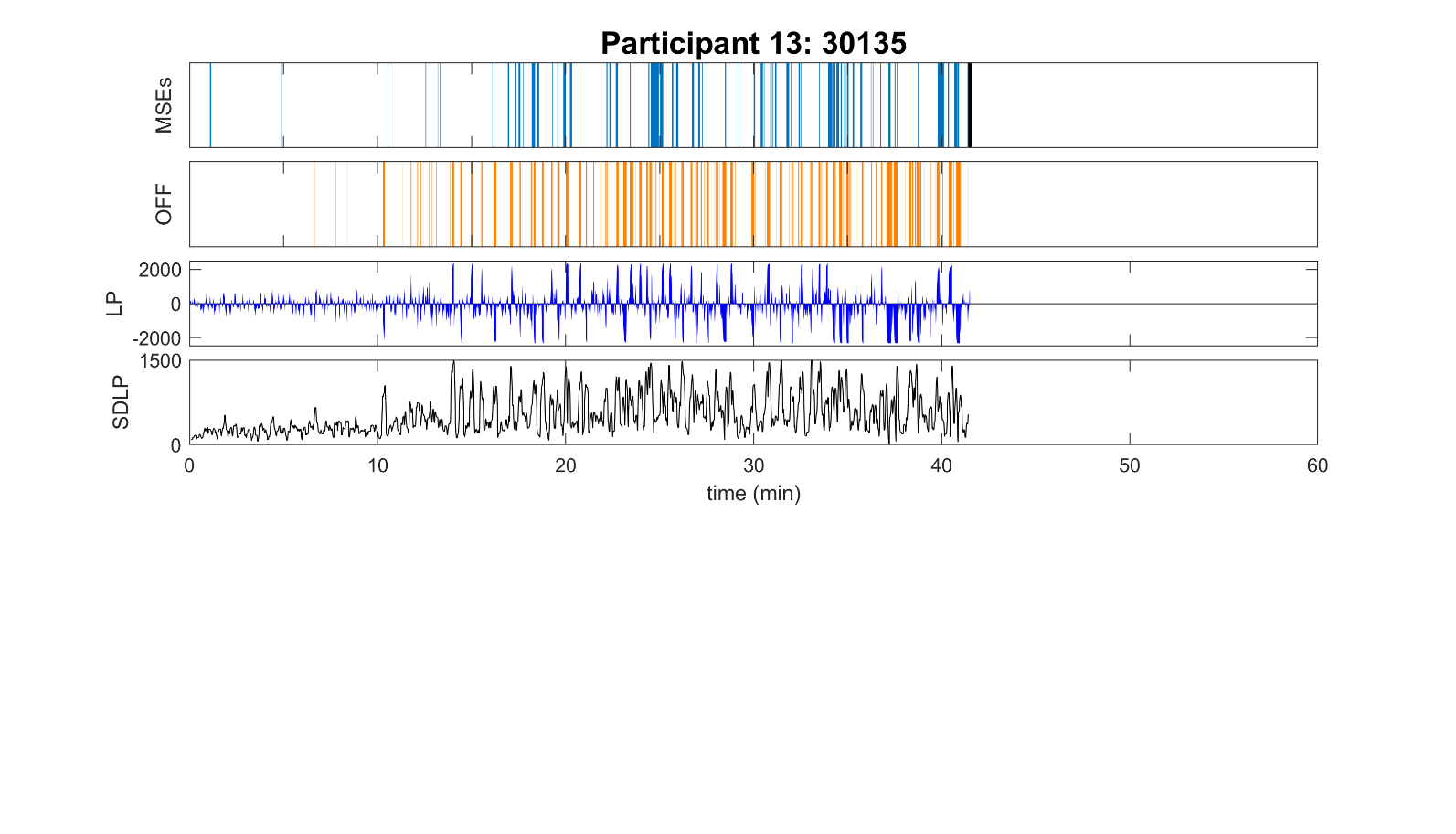


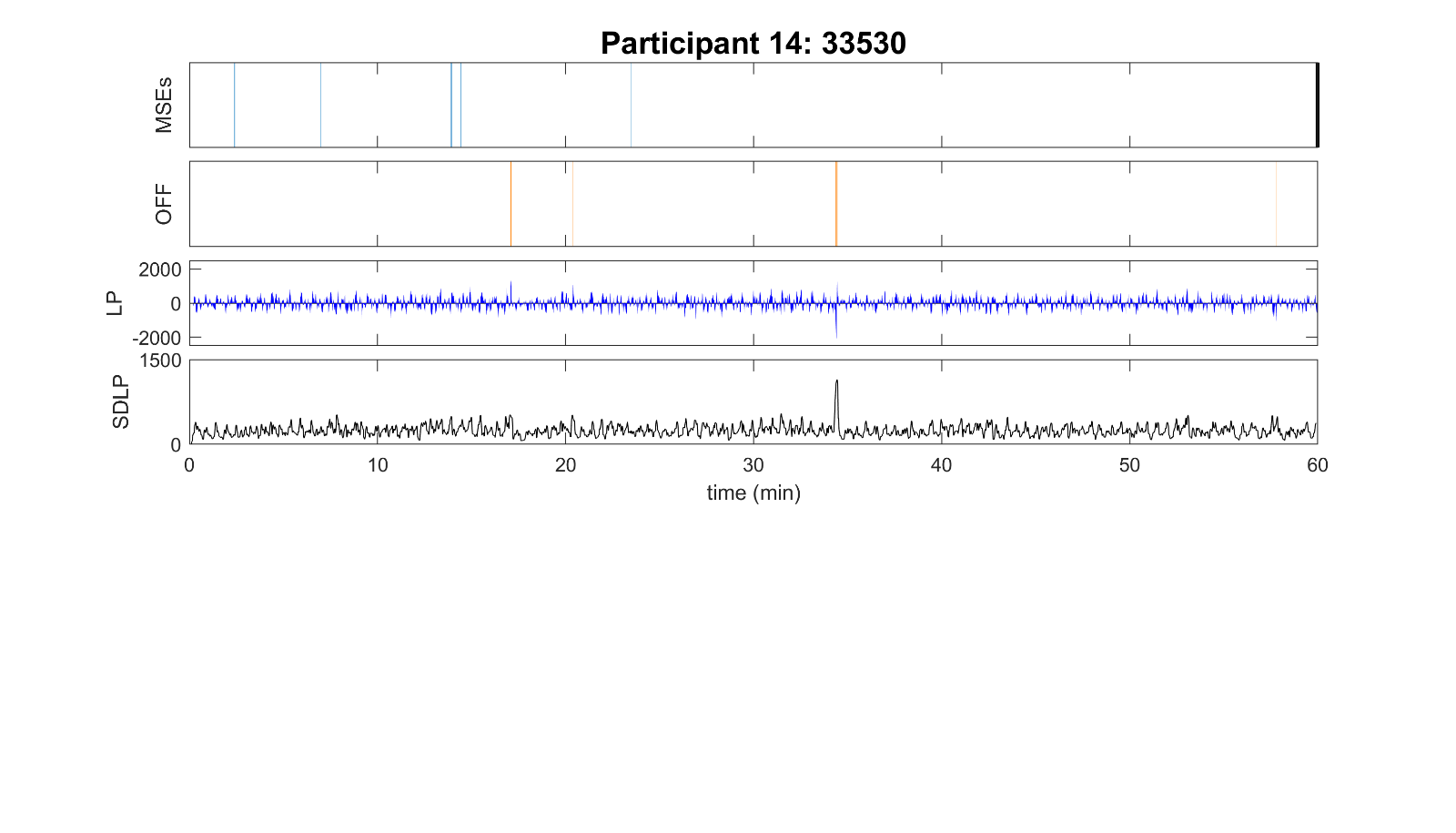


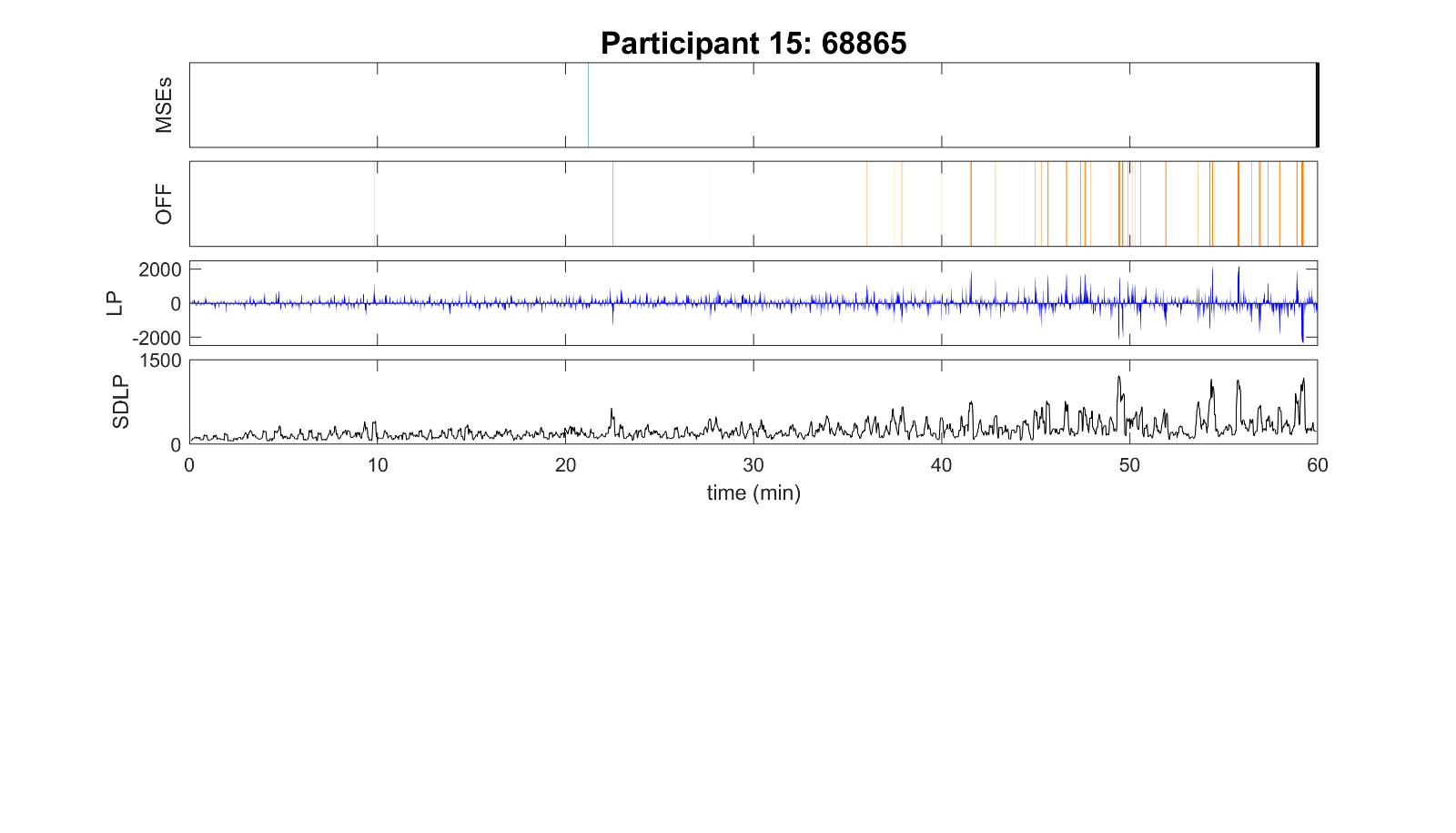


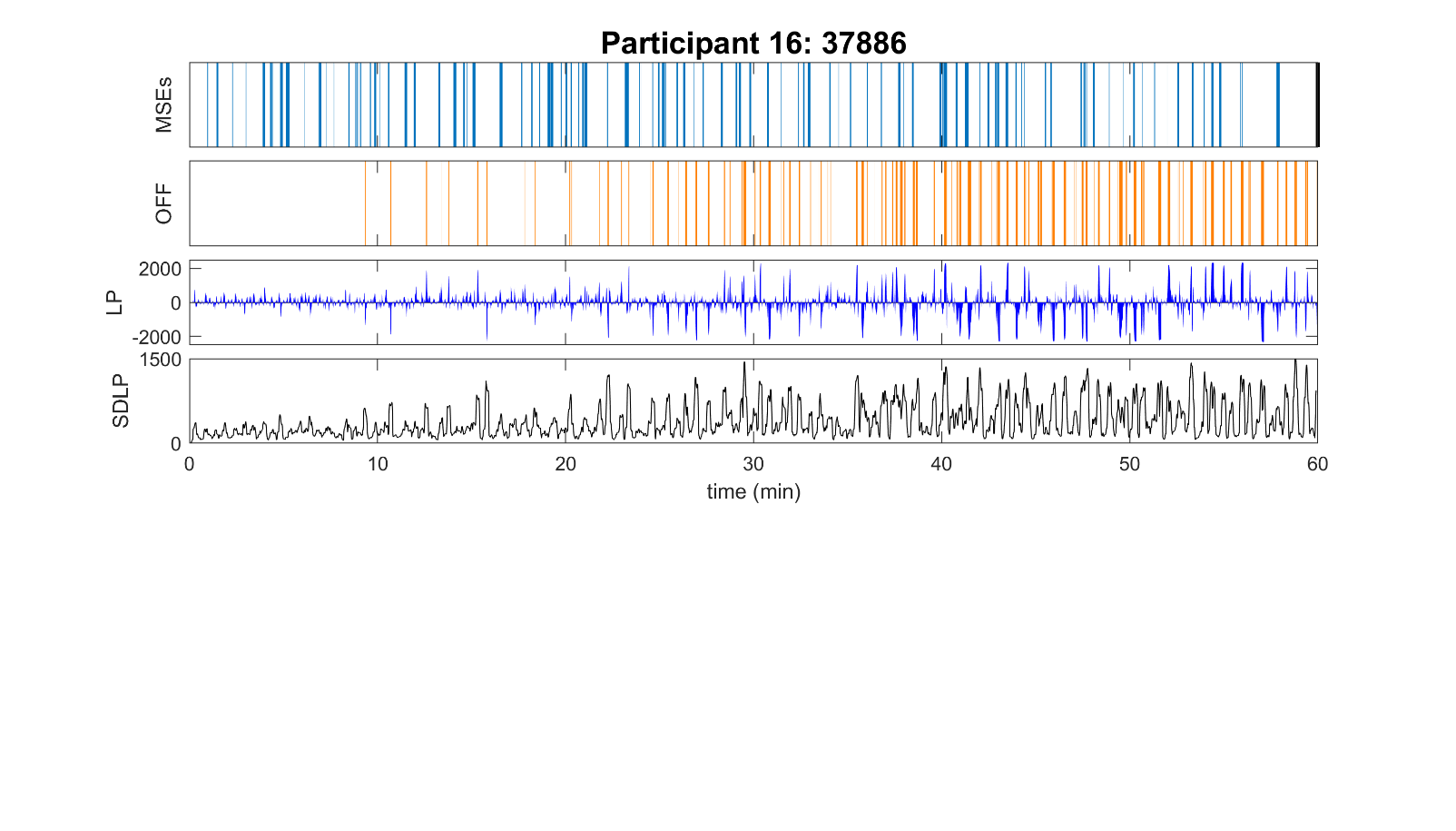


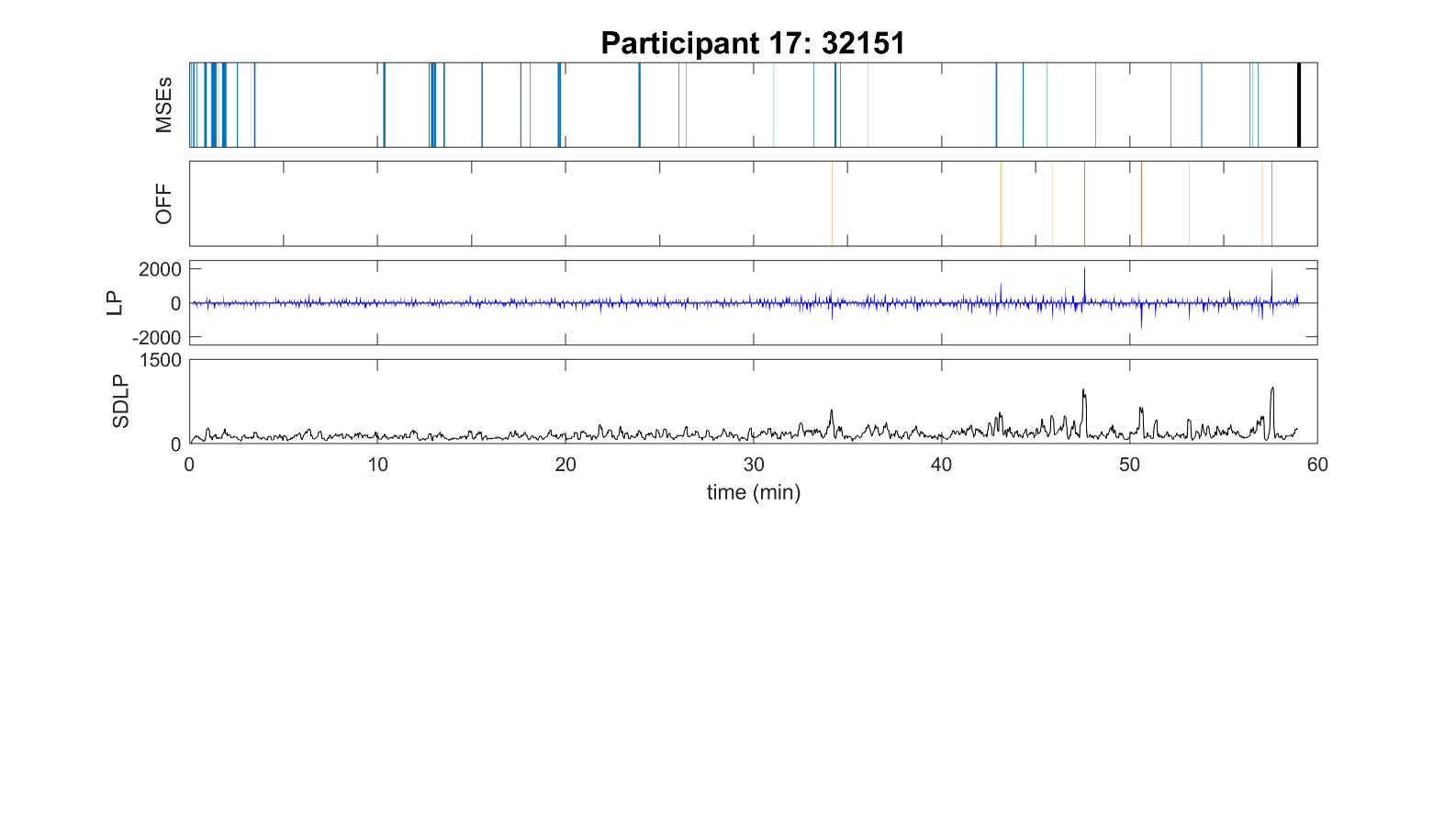


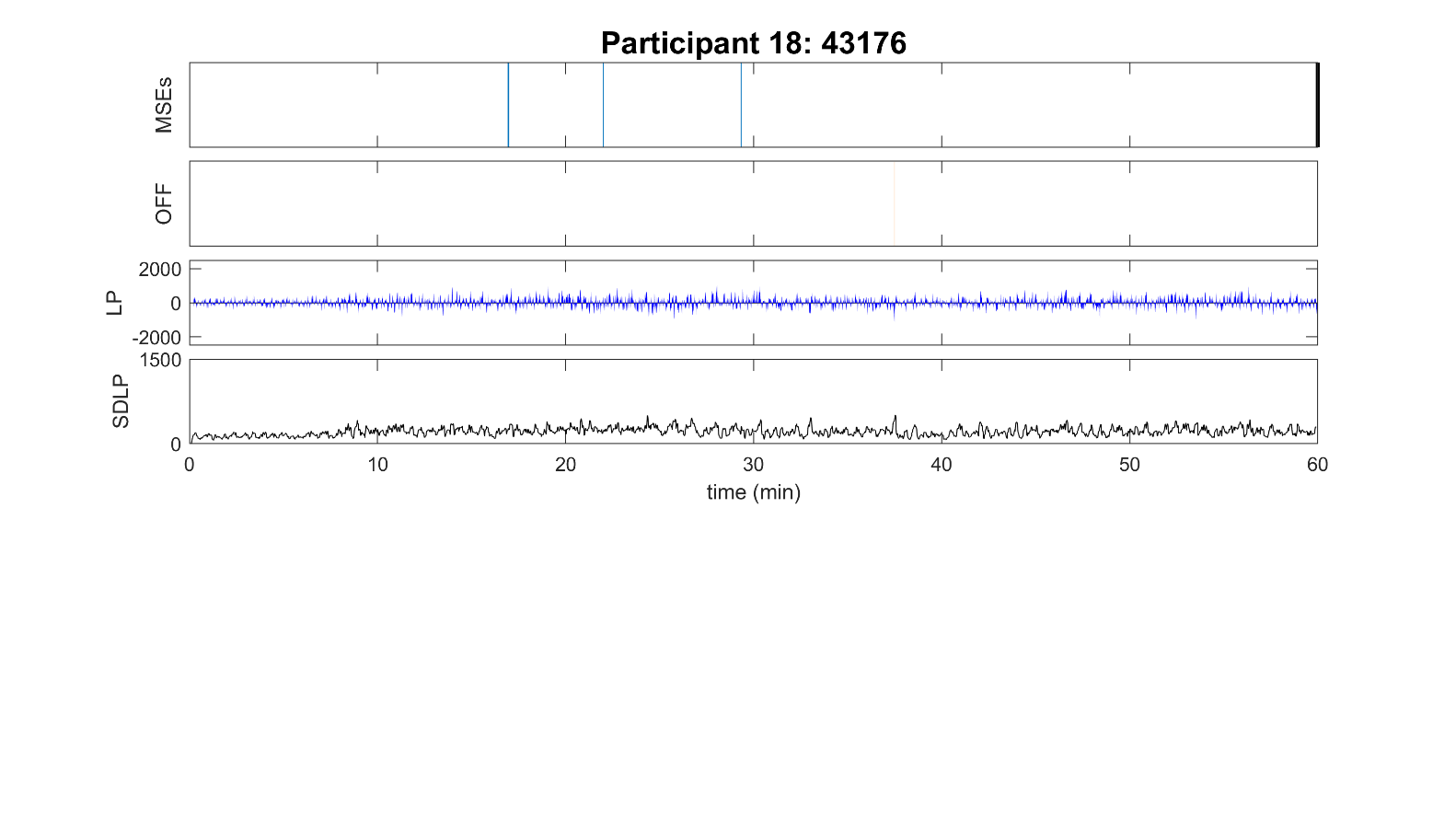


## **Figure S3:** Scatterplots of selected significant correlations between microsleep episodes (MSEs), latency to the first MSE (MSE-L), sleep onset (AASM-L) and off-road events (OFF) in the maintenance of wakefulness test (MWT; A-B), in the driving simulator (DSim; C-E), and between the MWT and the DSim (F-J). The Spearman correlation coefficient (Rs) is specified in each scatterplot. Data after sleep deprivation were analysed until AASM-L.


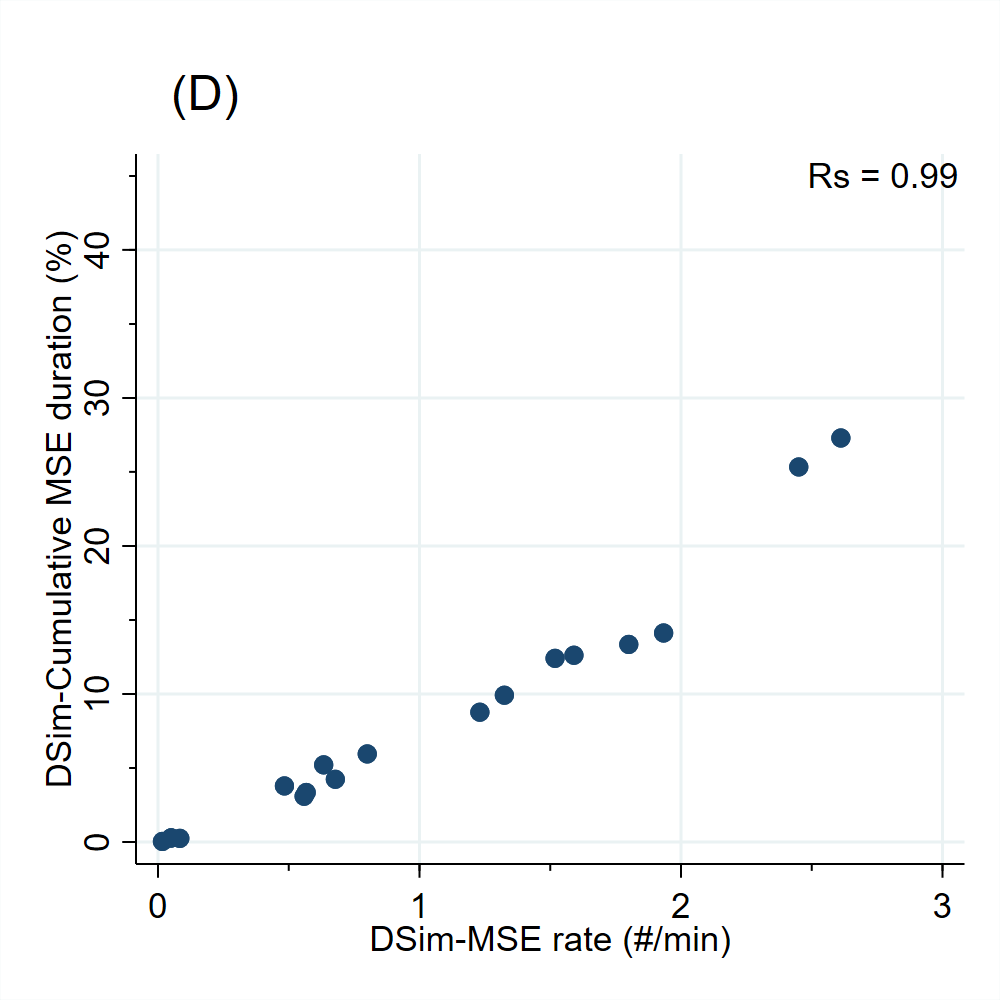

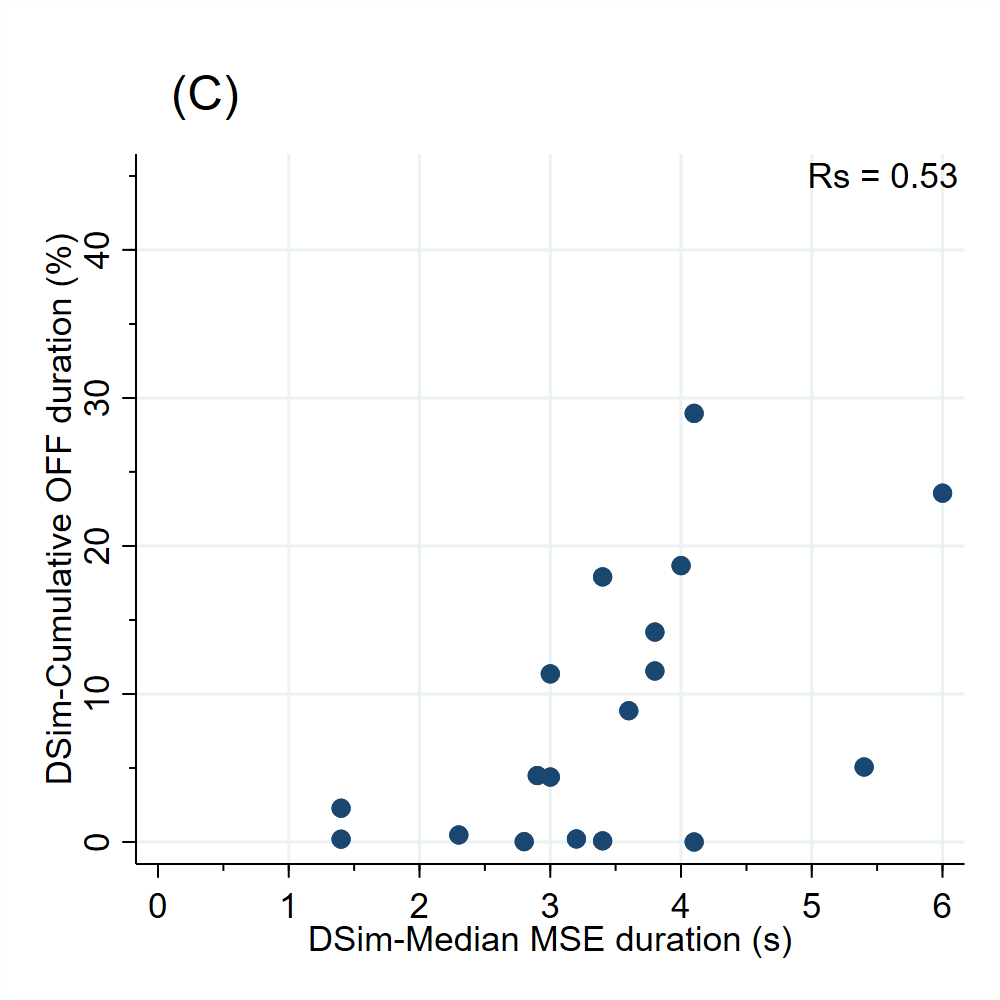

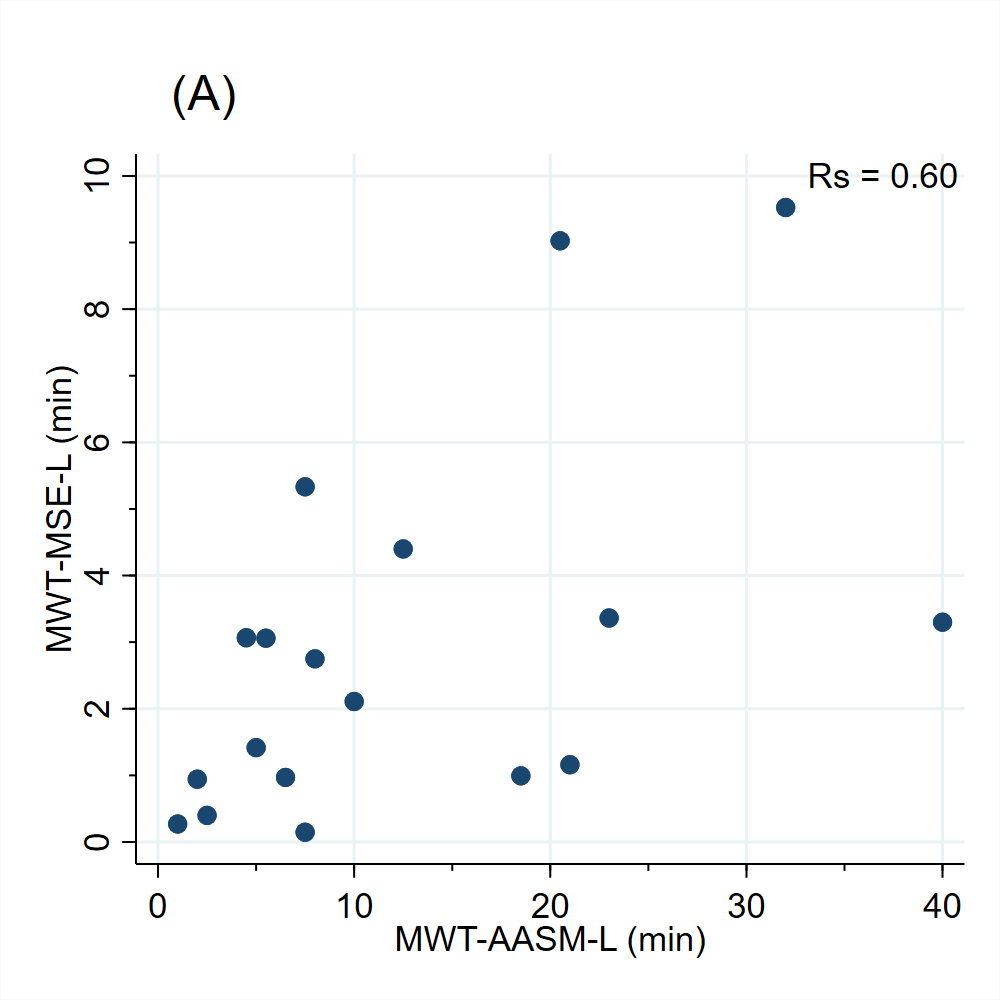

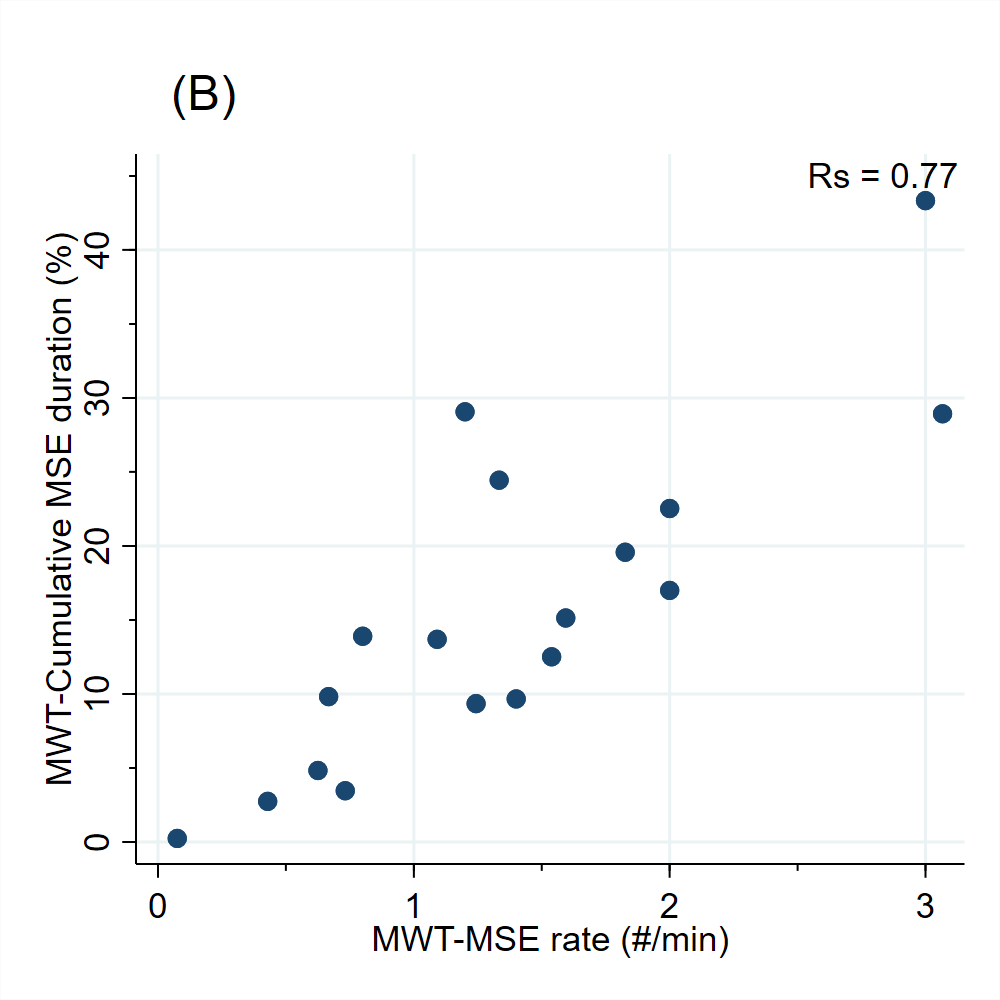


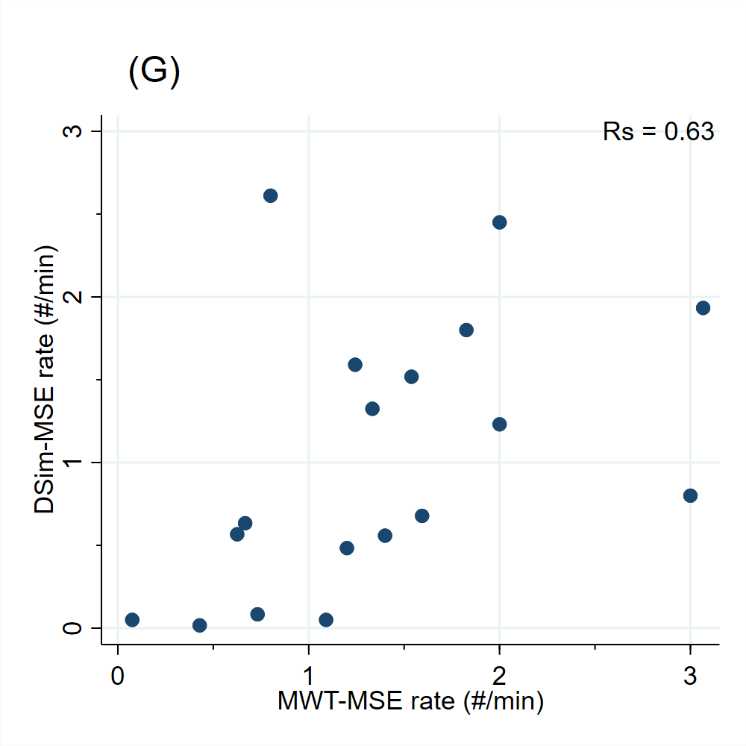

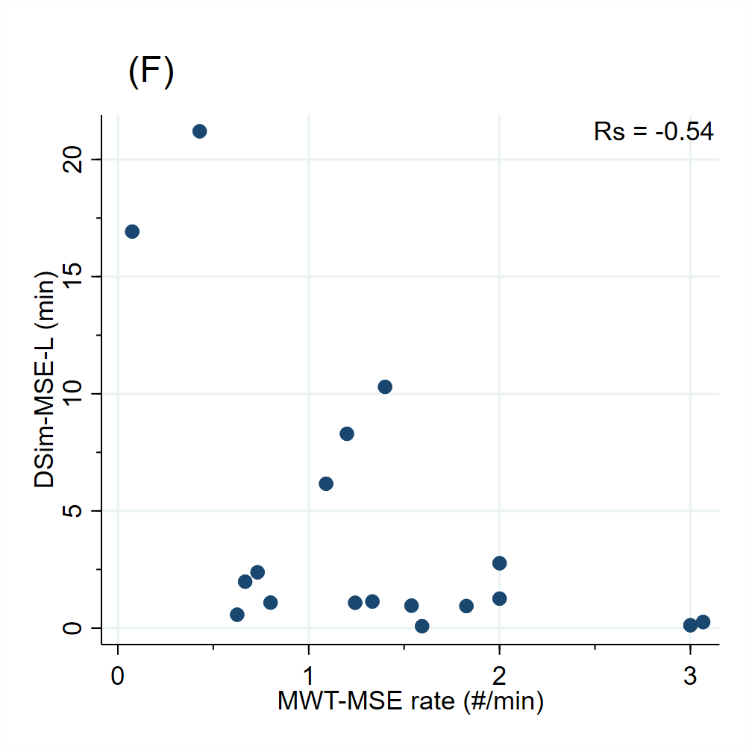

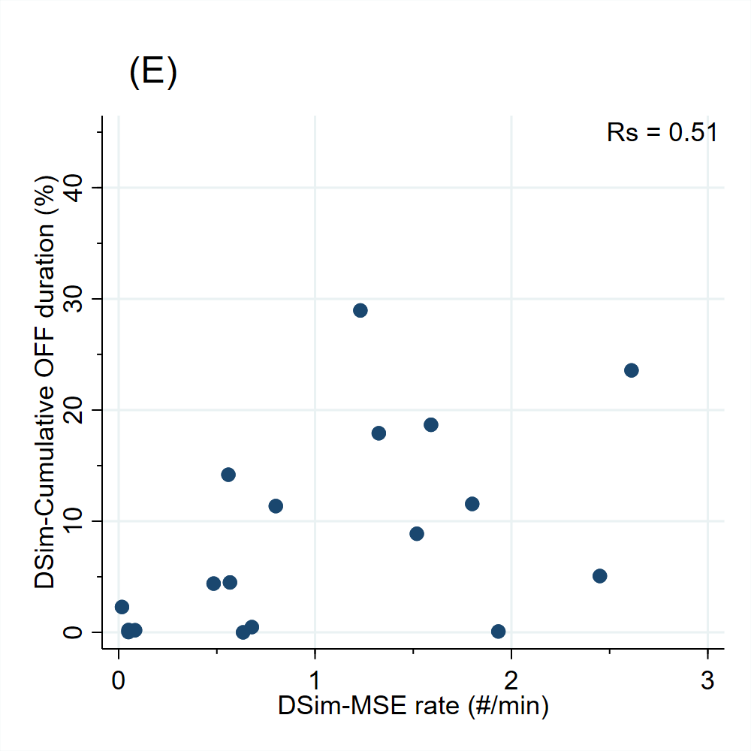


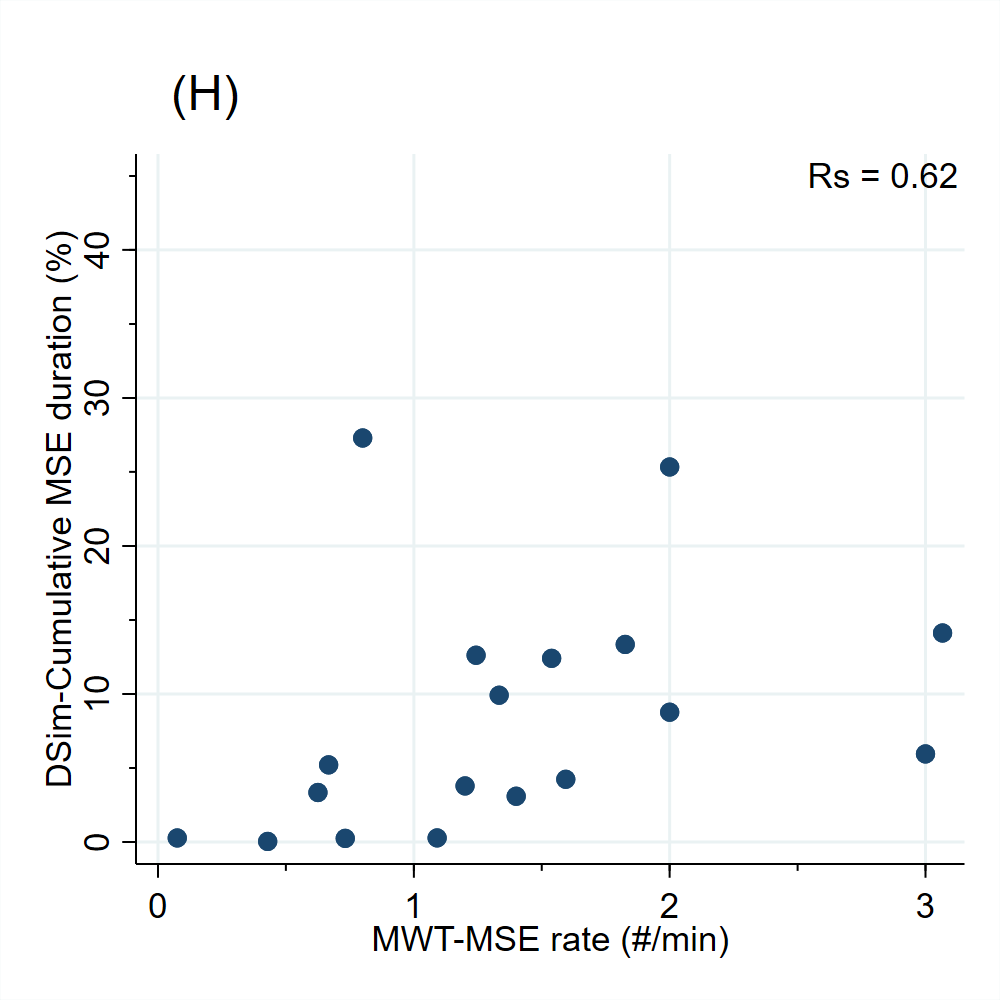


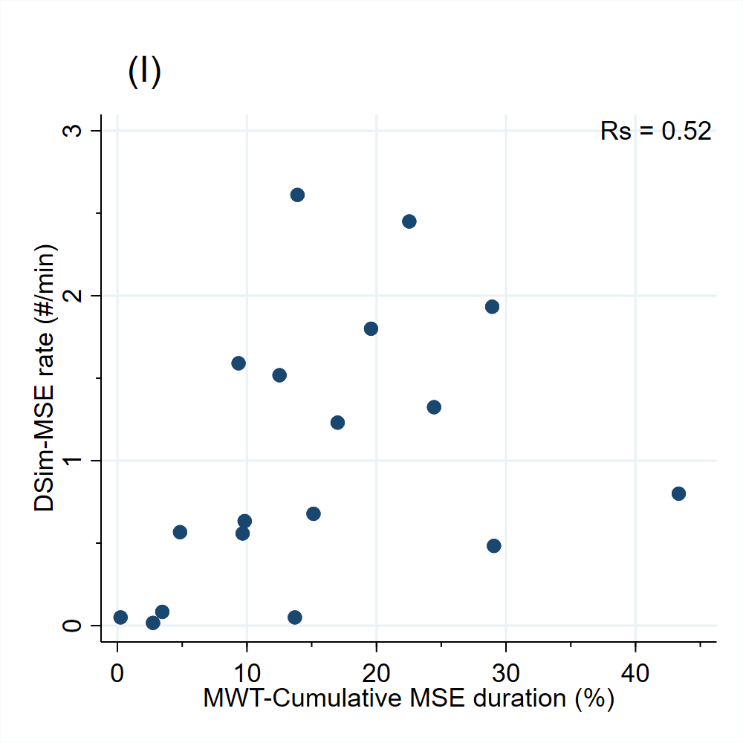

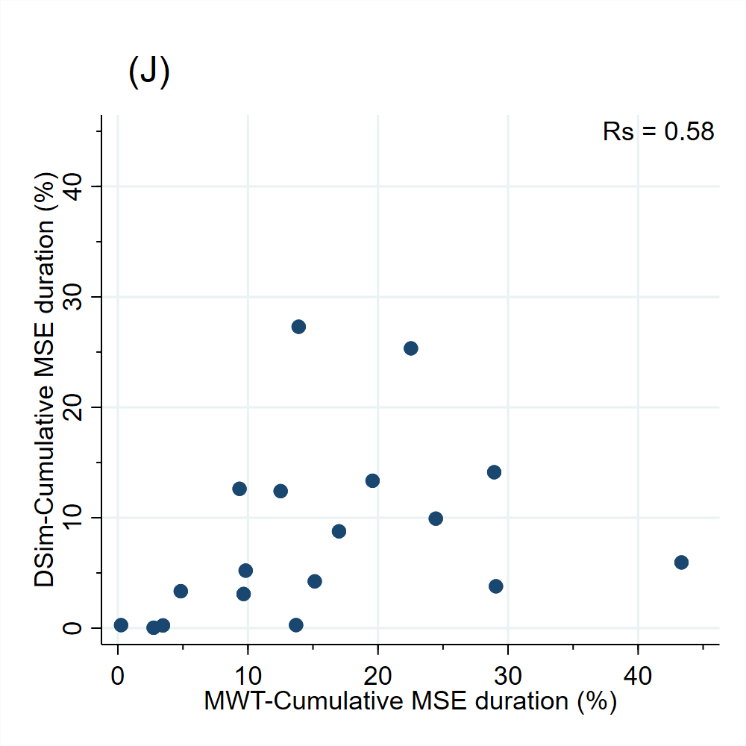


## **Figure S4:** (A, C) Rate (#/10 min) and (B, D) cumulative duration (s/10 min) of microsleep episodes (MSEs) and off-road events (OFF) during the maintenance of wakefulness test (MWT) and driving simulator (DSim) after sleep deprivation are illustrated for ‘very sleepy’ (A, B) and ‘less sleepy’ (C, D) participants. Data were analysed until AASM-defined sleep onset and displayed in 10-min bins (4 boxplots for the MWT and 6 for the DSim). Overall data are shown in Figure 4.


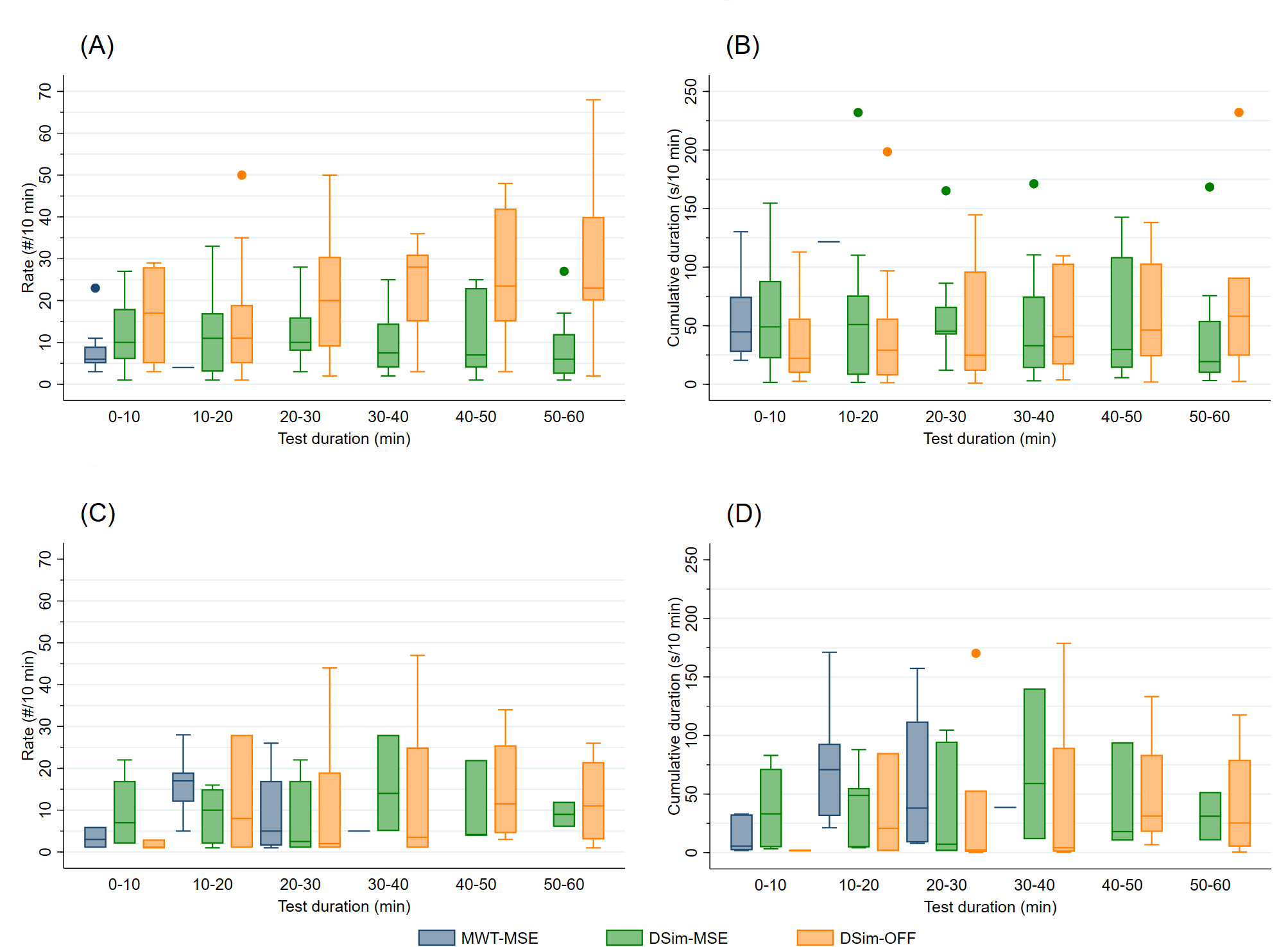


## **Figure S5:** Relation between microsleep episodes (MSEs) in the driving simulator and driving performance after sleep deprivation. The following data were analysed until AASM-defined sleep and displayed in 1-min bins: cumulative duration of microsleep episodes (MSEs, first panel, % of 1-min bin) and off-road events (OFF, second panel, % of 1-min bin), and standard deviation of lateral position (SDLP, third panel, a.u.). Participants are numbered according to Figure 3A. Three patterns were found: (A) simultaneous increasing MSE duration and deteriorating driving performance, (B) very few MSEs and good driving performance, and (C) large variation in the occurrence of MSEs without any visible impact on driving performance.

**
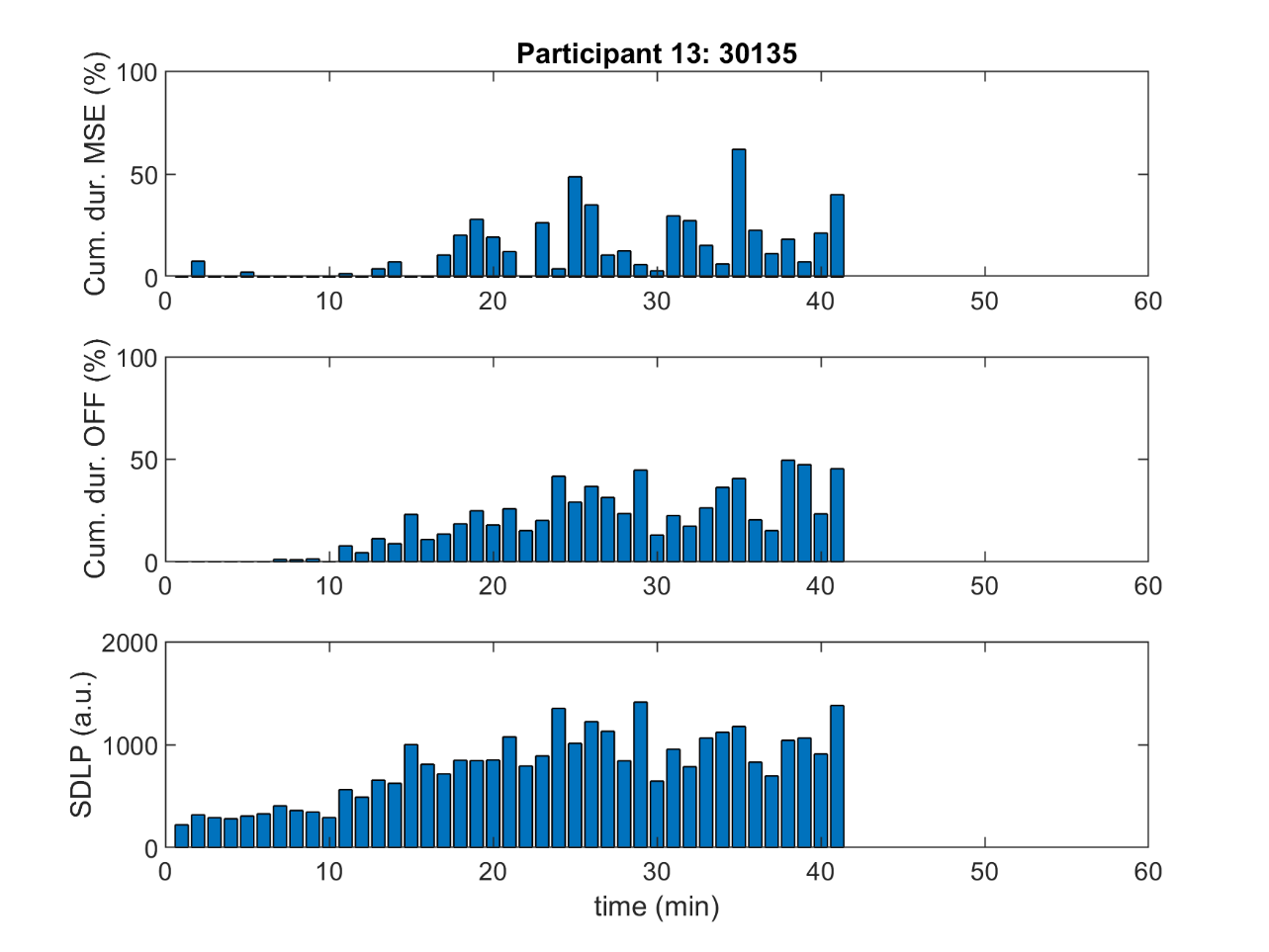
**

(A)


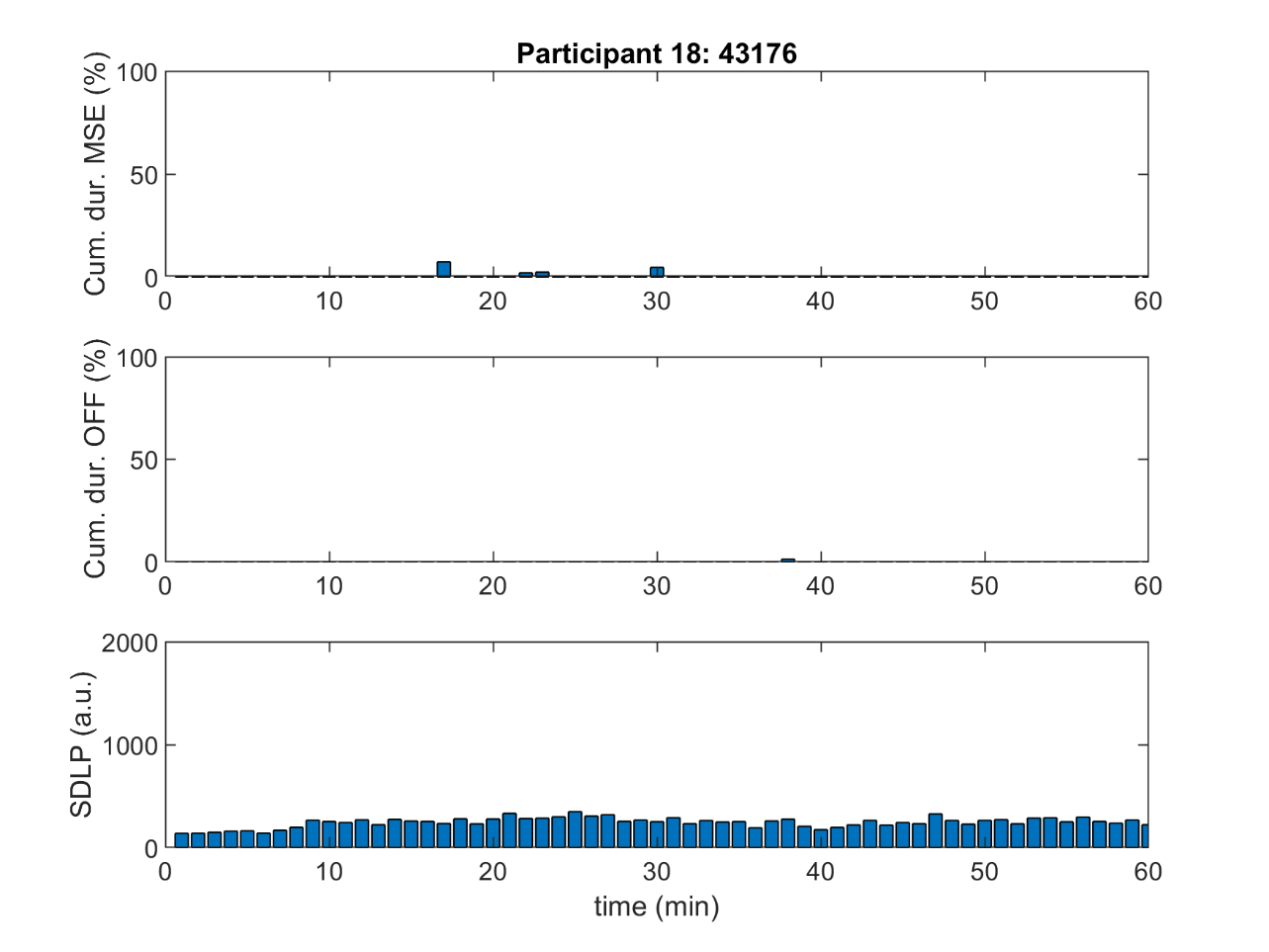


(B)


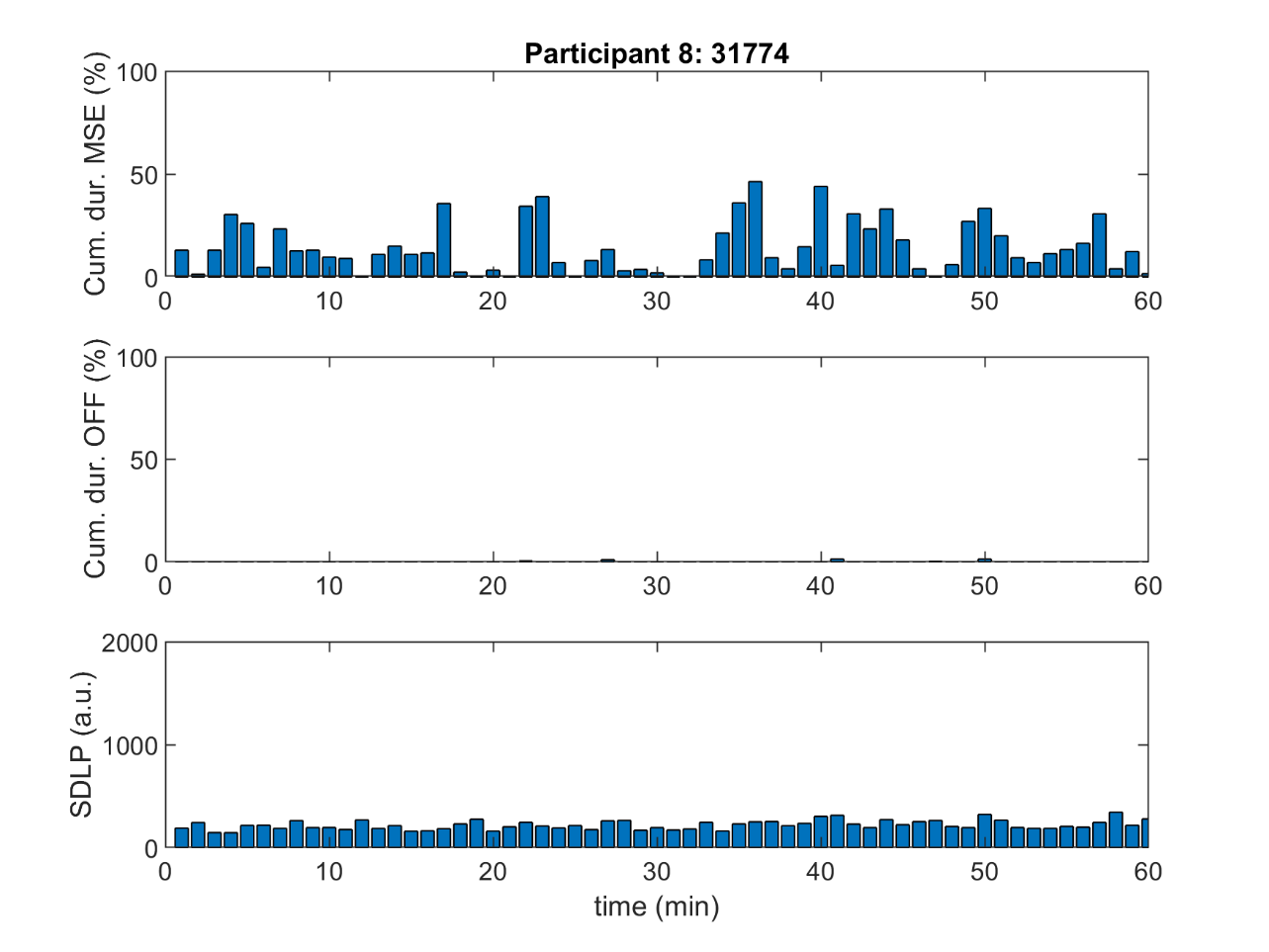


(C)
